# Tracking Nongenetic Evolution from Primary to Metastatic ccRCC: TRACERx Renal

**DOI:** 10.1158/2159-8290.CD-24-0499

**Published:** 2025-01-09

**Authors:** Ángel Fernández-Sanromán, Annika Fendler, Benjy J.Y. Tan, Anne-Laure Cattin, Charlotte Spencer, Rachael Thompson, Lewis Au, Irene Lobon, Husayn Ahmed Pallikonda, Alice Martin, Fiona Byrne, Antonia Franz, Anna Mikolajczak, Haseeb Rahman, Zayd Tippu, Scott T.C. Shepherd, Hugang Feng, Daqi Deng, Andrew Rowan, Lisa Pickering, Andrew J.S. Furness, Kate Young, David Nicol, Sarah Maria Rudman, Tim O’Brien, Kim Edmonds, Ashish Chandra, Steve Hazell, Kevin Litchfield, George Kassiotis, James Larkin, Samra Turajlic

**Affiliations:** 1 Cancer Dynamics Laboratory, The Francis Crick Institute, London, United Kingdom.; 2 Spanish National Cancer Research Center (CNIO), Madrid, Spain.; 3 Department of Urology, Charité – Universitätsmedizin Berlin, Corporate Member of Freie Universität Berlin and Humboldt Universität zu Berlin, Berlin, Germany.; 4 Skin and Renal Unit, Royal Marsden NHS Foundation Trust, London, United Kingdom.; 5 Retroviral Immunology, The Francis Crick Institute, London, United Kingdom.; 6 Department of Medical Oncology, Peter MacCallum Cancer -Centre, Melbourne, Australia.; 7 Sir Peter MacCallum Department of Oncology, The University of Melbourne, Victoria, Australia.; 8 Experimental Histopathology Unit, The Francis Crick Institute, London, United Kingdom.; 9 Cancer Evolution and Genome Instability Laboratory, The Francis Crick Institute, London, United Kingdom.; 10 Department of Urology, the Royal Marsden NHS Foundation Trust, London, United Kingdom.; 11 The Institute of Cancer Research, London, United Kingdom.; 12 Department of Oncology, Guy’s and St Thomas’ NHS Foundation Trust, London, United Kingdom.; 13 Urology Centre, Guy’s and St Thomas’ NHS Foundation Trust, London, United Kingdom.; 14 The Royal Marsden Hospital, London, United Kingdom.; 15 Cellular Pathology Department, St Thomas’ Hospital, London, United Kingdom.; 16 Department of Pathology, the Royal Marsden NHS Foundation Trust, London, United Kingdom.; 17 Tumour Immunogenomics and Immunosurveillance (TIGI) Lab, UCL Cancer Institute, London, United Kingdom.

## Abstract

Although the key aspects of genetic evolution and their clinical implications in clear-cell renal cell carcinoma (ccRCC) are well documented, how genetic features coevolve with the phenotype and tumor microenvironment (TME) remains elusive. Here, through joint genomic–transcriptomic analysis of 243 samples from 79 patients recruited to the TRACERx Renal study, we identify pervasive nongenetic intratumor heterogeneity, with over 40% not attributable to genetic alterations. By integrating tumor transcriptomes and phylogenetic structures, we observe convergent evolution to specific phenotypic traits, including cell proliferation, metabolic reprogramming, and overexpression of putative cGAS–STING repressors amid high aneuploidy. We also uncover a coevolution between the tumor and the T-cell repertoire, as well as a longitudinal shift in the TME from an antitumor to an immunosuppressive state, linked to the acquisition of recurrently late ccRCC drivers 9p loss and *SETD2* mutations. Our study reveals clinically relevant and hitherto underappreciated nongenetic evolution patterns in ccRCC.

**Significance:**

Using joint genomic–transcriptomic analysis of 243 samples, we reveal recurrent patterns of nongenetic evolution in ccRCC not exclusively governed by genetic factors, including T-cell depletion, tumor T-cell receptor coevolution, potential cGAS–STING repression, and increased cell proliferation. These patterns can aid clinical management and guide novel treatment approaches.

## Teaser

A genomic and transcriptomic analysis of patients with clear-cell renal cell carcinoma reveals clinically relevant patterns of nongenetic evolution, including progressive immune dysfunction and cGAS–STING suppression.

## Introduction

Clear-cell renal cell carcinomas (ccRCC) frequently exhibit genetic intratumor heterogeneity (ITH) that can fuel disease evolution (1). In the context of the ongoing TRACERx Renal study, we previously resolved the patterns of driver event co-occurrence, mutual exclusivity, and ordering, demonstrating that ccRCC can be categorized into seven different evolutionary subtypes (2). The patterns of clonal evolution in these trajectories range from punctuated, with early fixation of multiple driver events and aneuploidy that associate with rapid metastases, to highly branched, with extensive parallel evolution of distinct clones and attenuated metastatic progression (2, 3). These observations lay a strong motivation to further investigate the bases of clonal evolution in ccRCC.

The tumor genome traces past somatic events which serve to reconstruct an evolutionary history, but it does not record the concurrent evolution of the tumor cell phenotype and its microenvironment. Therefore, prior studies with an exclusive focus on genomics could not determine the impact of selected genetic events on the phenotype nor the corresponding evolution of the tumor microenvironment (TME), leaving a significant knowledge gap. For instance, the loss of chromosome arms 9p and 14q is necessary for metastatic progression (3), but the phenotype that underpins metastatic potential is unknown. Knowledge of the phenotypes under selection can unveil new therapeutic vulnerabilities, and understanding TME evolution could guide the selection and scheduling of the current therapies targeting angiogenesis and immune checkpoints.

Gene expression, as evaluated through RNA sequencing (RNA-seq), provides a snapshot of the tumor phenotype and the composition of the TME at the time of sampling. Consequently, paired multiregional DNA and RNA-seq of a tumor enables the investigation of the covariation of the tumor genome, transcriptome, and TME (4–6). In a recent study, significant transcriptional ITH was observed in advanced stage treated ccRCC, underscoring a prevalent sampling bias affecting expression-based biomarkers (7). However, this study did not comprehensively explore transcriptional evolution and its relationship with genetic changes in earlier disease stages or treatment-naive metastases.

In this study, we integrated multiregional RNA and DNA profiling and phylogenetic reconstruction to delineate the evolution of the TME and transcriptional changes from tumor initiation to metastases in 79 treatment-naive patients enrolled in the TRACERx Renal study (NCT03226886). Our findings reveal widespread nongenetic ITH not entirely determined by genetic evolution. This adds a layer of complexity to ccRCC evolution, with nongenetic evolution contributing to a substantial portion of functional ITH.

## Results

### TRACERx Renal RNA-seq Cohort

The TRACERx Renal cohort provides a distinct opportunity to evaluate the transcriptional patterns in clonally resolved tumors and link them to clinical outcomes. We performed full-length bulk RNA-seq on 243 samples from 79 patients enrolled in the TRACERx Renal study (NCT03226886). Our cohort includes 191 primary tumor regions, 22 matched metastatic regions, 18 matched tumor thrombus regions, and 12 tumor-adjacent normal samples. The cohort is reflective of the entire disease spectrum, with 18 stage I, 4 stage II, 25 stage III, and 27 stage IV tumors (Supplementary Fig. S1).

All samples under study were previously profiled for genetic alterations (2, 3), including somatic mutations in driver genes and driver somatic copy number alterations (SCNA). Tumors included in this cohort had varying degrees of genetic ITH (range: 0–13.5) and aneuploidy [weighted genome instability index (wGII; range: 0.01–0.93)], both important features of clonal evolution. Canonical ccRCC drivers were frequently subclonal in this cohort, including *PBRM1* (6/79 patients, 7.6%), *SETD2* (9/79 patients, 11.4%), 9p loss (25/79 patients, 31.6%), and 14q loss (29/79 patients, 36.7%). This provides a unique opportunity for within-patient comparison of tumor regions with and without a given somatic aberration, hence controlling for the impact of patient-specific factors on transcriptional changes (see Supplementary Note S1 for a more detailed description of how prior TRACERx Renal results are incorporated into this study).

### Pervasive Transcriptional Inter- and Intratumor Heterogeneity in ccRCC

**Figure 1.**
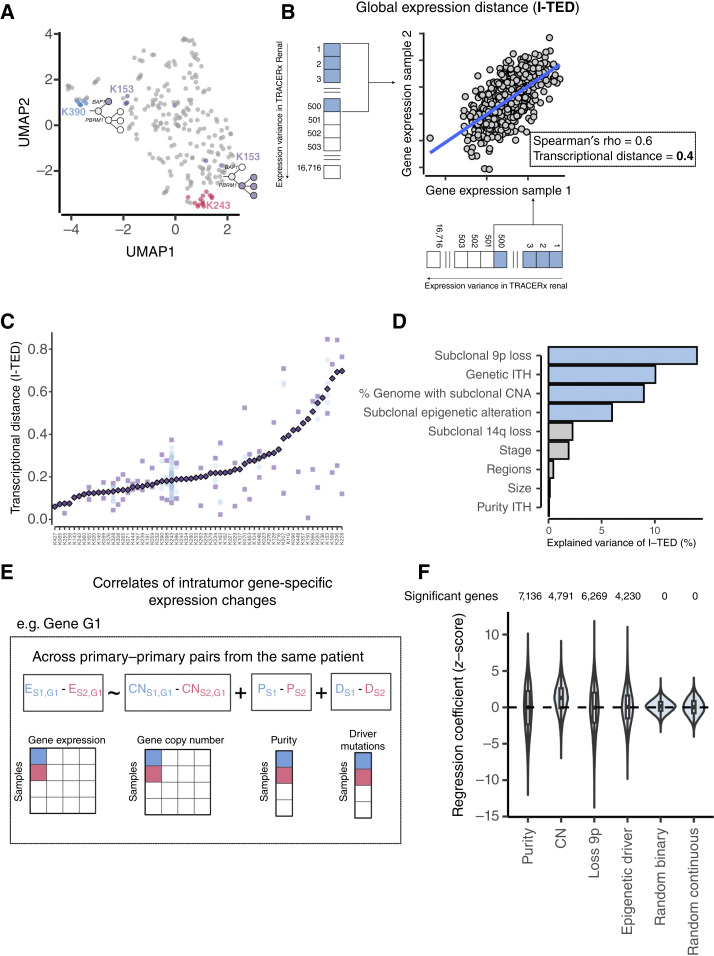
Transcriptional inter- and intratumor heterogeneity is pervasive in TRACERx Renal. **A,** UMAP visualizing the transcriptional variation across 231 tumor samples (gray points). Samples from patients K390, K243, and K153 are highlighted to illustrate the varied levels of transcriptional ITH in distinct patients. For patient K153, we highlight the phylogenetic tree branch containing the clones observed in the adjacent sample points. **B,** Schematic representation of the calculation of transcriptional distance between two samples from the same patient (see Supplementary Fig. S3A for generalization for multi-sample patient, I-TED). The correlation (*r*) between the expression of the top 500 most variable genes across the TRACERx Renal cohort in both samples informs on the degree of transcriptional similarity between both samples. The final estimate of transcriptional distance is defined as 1−r. I-TED is estimated by repeating this procedure across all different pairs of samples within a patient and averaging the observed transcriptional distances (see “Methods”; Supplementary Fig. S3A for details). **C,** Primary tumor I-TED values (purple diamonds) across 60 TRACERx Renal patients with at least two regions sampled. Pale purple squares represent pairs with minimum and maximum transcriptional distances; blue points represent the transcriptional distance between the rest of sample pairs, if available. **D,** Proportion of the variance in primary tumor I-TED scores in TRACERx Renal explained by nine selected genomic and clinical features (see “Methods”) on a multivariate linear model of I-TED values across 60 tumors with at least 2 sampled primary tumor regions. Variables in blue and gray bars display significant (FDR < 0.05) and nonsignificant associations, respectively. **E,** Schematic representation of a linear regression framework to identify correlates of within-patient expression changes in each of 14,120 genes, including *cis* effects of increased/decreased gene dosage and variation in purity. Changes in gene expression and copy number are gene and pair specific; changes in purity and driver mutation (or target variable) are pair specific and constant across different genes. For each gene, a linear mixed-effects model is fitted with the values obtained across all possible primary–primary pairs of patients, accounting for patient of origin and whole-genome doubled status, included as random covariates in the model (see “Methods” for details). **F,** Distribution of regression coefficients for linear mixed-effects models fit for each of 14,120 genes and each of the variables included in the model, with a total number of genes significantly (FDR < 0.05) associated with a variable highlighted at the top. To allow comparison of target variables with a background expectation, random binary and continuous covariates were inputted to the model in **E**. Boxes extend from the lower to the upper quartiles, with the line inside the box representing the median, with whiskers indicating data within 1.5 times the interquartile range (IQR) from the quartiles; violins represent the distribution of values per group.

To visualize transcriptional variation within the TRACERx Renal cohort, we applied uniform manifold approximation and projection (UMAP) dimensionality reduction to normalized gene expression counts across the 231 tumor samples (Fig. 1A). As anticipated, we could observe that samples from the same patient tended to cluster in UMAP space (patients K243 and K390 highlighted in Fig. 1A; Supplementary Fig. S2A and S2B). Accordingly, we observed *inter*-patient transcriptional heterogeneity to be 2.9-fold (95% CI, 2.7–3.0) higher than transcriptional ITH (Supplementary Fig. S2C). Nonetheless, significant transcriptional differences were observed among distinct samples from the same patient. For instance, in patient K153, two distinct clusters could be observed, consistent with their separate phylogenetic branches associated with *BAP1* and *PBRM1* mutations, respectively (Fig. 1A). This highlights the presence of considerable transcriptional ITH in ccRCC.

To quantify transcriptional ITH, we adapted a previously published methodology to condense the extent of transcriptional differences between tumor samples from the same patient into a single metric: intratumour expression distance. (I-TED, see “Methods”; Fig. 1B; Supplementary Fig. S3A and S3B). This metric is defined using the top 500 with highest expression variance in the TRACERx Renal cohort (Supplementary Table S1). I-TED scores across the TRACERx Renal cohort varied more than 10-fold (range: 0.06–0.7; Fig. 1C), reflecting a wide range of transcriptional heterogeneity in ccRCC, from largely homogeneous to highly heterogeneous tumors. Unlike genetic ITH (2), higher transcriptional ITH did not associate with poorer outcomes (Supplementary Fig. S4A and S4B).

Next, we investigated the factors associated with variance in I-TED scores through a multivariate analysis including nine clinical and genetic features (see “Methods”). We confirmed that neither the total number of profiled tumor regions per case nor variation in tumor purity confounded I-TED estimates (Fig. 1D). Instead, we observed that high I-TED values were significantly associated with both genetic ITH and the fraction of the genome affected by subclonal copy number alterations (i.e., copy number heterogeneity), together explaining 19% of the variation in I-TED scores (Fig. 1D). Among individual subclonal copy number drivers, subclonal 9p loss contributed 10.7% of I-TED variance (Fig. 1D), more than any other copy number driver alteration (Supplementary Fig. S5A). Additionally, subclonal mutations in chromatin modifier genes—*KDM5C*, *ARID1A*, *SETD2*, *PBRM1*, and *BAP1*, which plausibly can induce significant transcriptional change through downstream epigenetic remodeling—explained 6% of the variance.

To understand how the factors that impact I-TED are associated with changes in the expression of individual genes, we applied a gene-level linear regression framework (see “Methods”). Here we jointly account for variation in tumor purity and explore the *cis* and/or *trans* effects of the factors that were associated with I-TED variance (Fig. 1E). Changes in gene dosage significantly [false discovery rate (FDR) < 0.05] explained the expression of 4,791 (34.2%) genes (Fig. 1F), with a strong positive association between gene dosage and gene expression, as expected. In agreement with the analysis of I-TED variance, subclonal 9p loss and mutations in chromatin modifier genes were associated with a significant downregulation of 3,210 (22.7%) and 2,033 (14.4%) genes and upregulation of 3,059 (21.7%) and 2,197 (15.6%) genes (Fig. 1F), respectively. Approximately 5,924 of the 6,269 genes with significant changes in expression upon 9p loss were located on other chromosomes (Supplementary Fig. S5B), suggesting that 9p loss associates with genome-wide expression changes. Overall, these factors explained 49.9% of transcriptional variation at gene level (Supplementary Fig. S6), consistent with the 43.8% of variance explained in the multivariate regression of I-TED scores (Fig. 1D). Taken together, this suggests that transcriptional diversification is extensively, but nonexclusively, driven by genetic variation in ccRCC.

### ccRCC Transcriptional Evolution Mirrors Clonal Evolution

**Figure 2.**
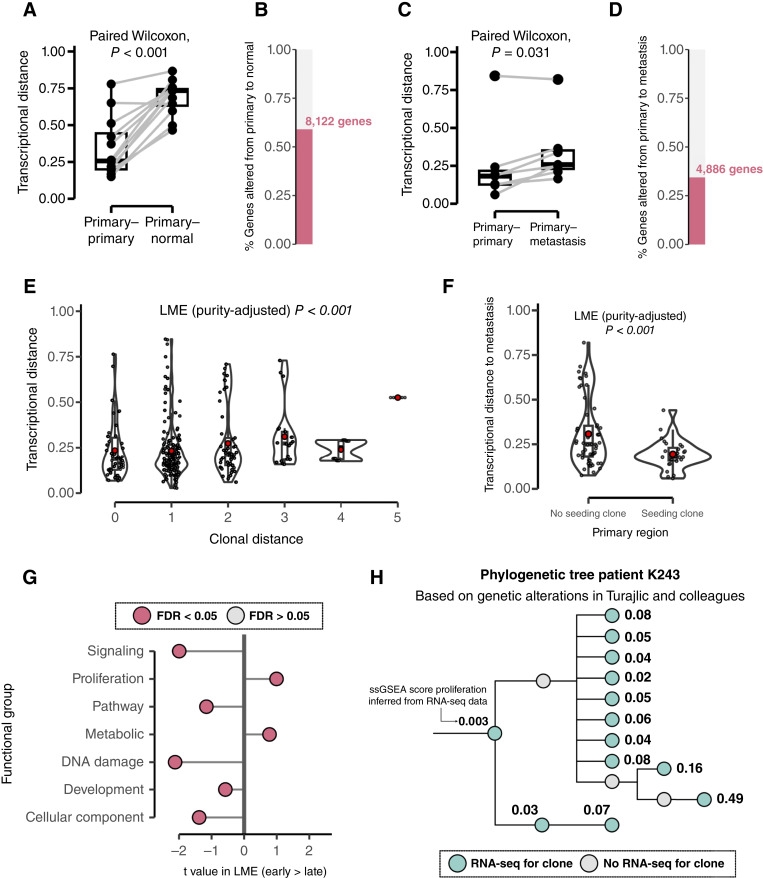
Transcriptional evolution mirrors clonal structure and follows recurrent trends. **A,** Transcriptional distance between primary tumor samples and other primary tumor samples or adjacent normal kidney samples (12 patients with both available primary–primary and primary–normal pairs). **B,** Proportion of genes significantly associated with changes in expression in normal–primary pairs using a gene-specific linear regression framework (see “Methods”). **C,** Transcriptional distance between primary tumor samples and other primary tumor samples or matched metastases (seven patients with both available primary–primary and primary–metastasis pairs) from the same patient. **D,** Proportion of genes significantly associated with changes in expression in primary–metastasis pairs using a gene-specific linear regression framework (see “Methods”). **E,** Transcriptional distances between 285 pairs of primary tumor samples with increasing clonal distances, defined as the distance between clones located in two different monoclonal regions. **F,** Transcriptional distance between metastatic samples and paired primary samples depending on whether the primary region contained a seeding clone (55 nonseeding primary–metastasis pairs; 27 seeding primary–metastasis pairs). **G,** Association of clone gene expression (see “Gene Expression and TME Assignment to Individual Tumor Clones” and Supplementary Fig. S11A and S11B) of the 50 hallmark gene signatures grouped into 7 functional groups [as defined by Martinez-Ruiz and colleagues (6)] and distance to the MRCA of the patient’s tumor. **H,** Phylogenetic tree for patient K243—previously constructed by Turajlic and colleagues (2) using identified somatic alterations—and ssGSEA scores for proliferation signature identified in bulk RNA-seq. Green nodes indicate clones with a gene expression profile assigned in this study (see “Gene Expression and TME Assignment to Individual Tumor Clones”), and gray clones only have genetic information obtained previously within above study (3), allowing full reconstruction of the phylogenetic tree. ssGSEA scores refer to cell proliferation inferred with the signature from Motzer and colleagues (45). Across all panels, LME (linear mixed-effects model) is used to control for inclusion of multiple samples from the same patient by including patient of origin as a random covariate. In (**E** and **F**), red points indicate mean values; boxes extend from the lower to the upper quartiles, with the line inside the box representing the median, with whiskers indicating data within 1.5 times the IQR from the quartiles; violins represents the distribution of values per group. In (**A** and **C**), for patients with more than one primary–primary, primary–normal, or primary–metastasis pair, the median transcriptional distance for each type of sample pair is calculated.

To understand the hierarchy of transcriptional diversification in ccRCC, we aimed to trace transcriptional changes throughout tumor development while controlling for patient-specific influences. First, we interrogated overall transcriptional changes at two pivotal stages of tumor evolution: malignant transformation and metastatic progression. To explore this, we calculated transcriptional distances between pairs of samples obtained from multiple tumor locations including tumor-adjacent normal tissue and spatially separate regions of the primary tumor and the associated metastases. Our analysis revealed significantly higher transcriptional distances between primary–normal and primary–metastasis pairs compared with primary–primary tumor pairs from the same patient, with the expression of 8,122 genes (3,753 downregulated and 4,369 upregulated) and 4,886 genes (2,489 downregulated and 2,397 upregulated) significantly changing from normal tissue to primary and from primary to metastatic samples, respectively (Fig. 2A and D). This supports that marked transcriptional differences occur during malignant transformation (8) and potentially during metastatic progression.

In particular, from adjacent normal kidney to primary tumor, we observed a significant increase in the expression of genes involved in proliferation, glycolysis, oxidative phosphorylation, and response to reactive oxygen species and a decrease in the expression of genes involved in mitotic spindle assembly and cell-to-cell junctions (Supplementary Table S2). The magnitude of these changes did not correlate with the distance to the most recent common ancestor (MRCA; Supplementary Fig. S7), perhaps suggesting a rapid transcriptional change upon malignant transformation. Meanwhile, going from primary tumor to metastasis, we identified increased expression of proliferation and metabolic pathways, including the overexpression of MYC or E2F targets and oxidative phosphorylation (Supplementary Table S3).

We next explored whether transcriptional diversification aligns with the clonal structure of the tumors in our cohort (2). To test this, for each primary tumor, we measured the pairwise transcriptional and clonal distances between distinct samples (Supplementary Fig. S8), in which the clonal distance is defined as the total number of (sub)clonal expansions spanning the divergence of the tumor clones identified in two distinct samples (see “Methods” for details; Supplementary Fig. S8). We observed that transcriptional distance monotonically increases with clonal distance (LME *P*-value < 0.001, corrected for differences in purity between biopsies; Fig. 2E), suggesting that, in spite of transcriptional plasticity, clonal evolution leads to progressive and stable changes in gene expression.

Given the marked transcriptional changes observed in metastases (Fig. 2C and D) and the potentially more plastic transcriptome of metastasis-seeding subclones, we next aimed to determine if at least a subset of the transcriptional changes acquired during primary tumor clonal evolution were maintained in established metastases. In this line, we first observed that the relationship between clonal and transcriptional distance is maintained when considering primary–metastasis pairs (LME *P*-value < 0.001, corrected for differences in purity between biopsies; Supplementary Fig. S9). Further, uniquely to the TRACERx Renal cohort, we can assign the clonal origin of metastasis to specific primary tumor regions (9). We observed that expression patterns in metastasis itself were closer to the metastasis-seeding than nonseeding regions in the matched primary tumor (Fig. 2F). This might imply that a significant portion of the transcriptional changes observed in established metastases were progressively acquired through heritable changes accrued during primary tumor evolution. Consequently, the primary sample with the least clonal distance to metastasis may best reflect the tumor-specific transcriptional signature of a metastatic sample (Supplementary Fig. S10) but not necessarily TME-related transcriptional signatures.

Finally, to understand recurrent trends in the transcriptional evolution from earlier to later clones within TRACERx Renal, we assigned a gene expression profile to individual clones (see “Gene Expression and TME Assignment to Individual Tumor Clones”; Supplementary Fig. S11A and S11B). Utilizing the gene expression profiles of each clone, we performed single-sample gene set enrichment analysis (ssGSEA) of 50 hallmark signatures, which we collapsed into seven functional groups (signaling, proliferation, pathway, metabolic, DNA damage, development, cellular component), as done in a previous study (Supplementary Table S4; ref. 6). Later-emerging clones were characterized by increased proliferative potential, activation of the FAS-pentose phosphate and omega oxidation metabolic pathways and unfolded protein response, and reduced DNA damage repair capacity and activation of signaling pathways such as PI3K/AKT/mTOR (Fig. 2G; Supplementary Table S5).

The increase in proliferation along the clonal structure suggests that later-emerging clones in ccRCC exhibit greater proliferative capacity than earlier ones. This might imply that at least some of the positive selection is driven by increased tumor’s proliferative potential, which would facilitate clonal expansion. This trend is exemplified in patient K243, in whom we observe a sequential increase in proliferation in later-emerging clones (Fig. 2H) and comparable proliferative potential of clones that expanded and were maintained in parallel.

### Somatic Copy Number Drivers of Metastatic Progression in ccRCC Induce Recurrent Transcriptional Changes

**Figure 3.**
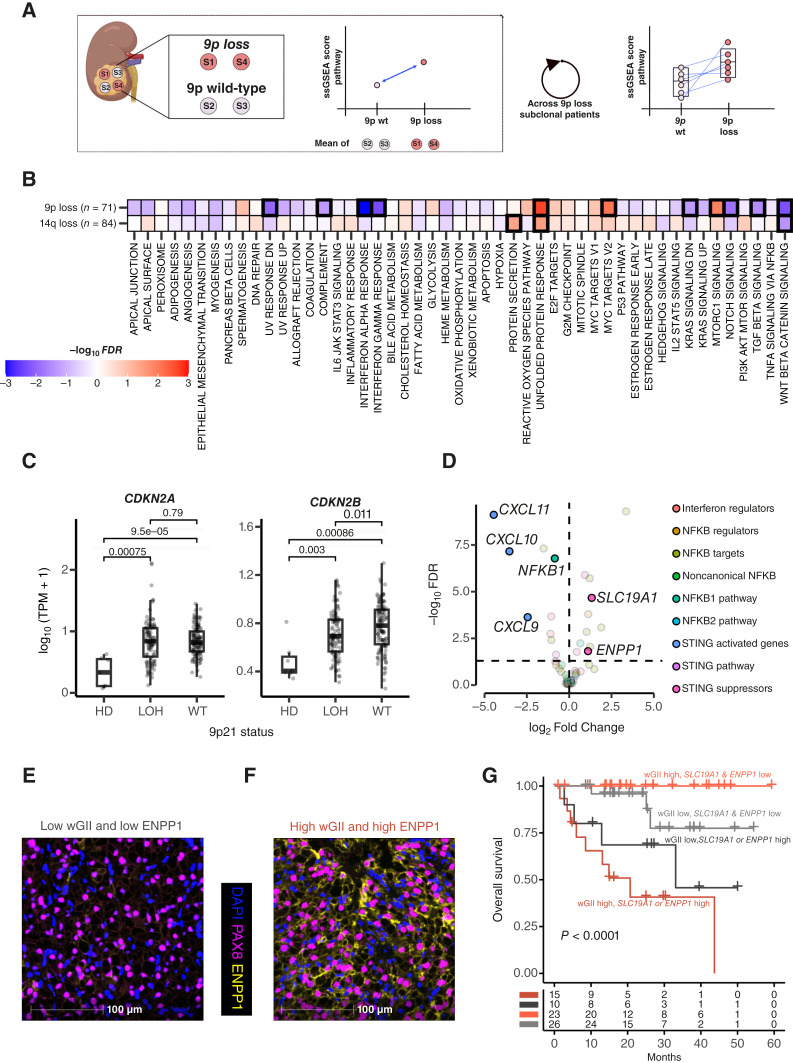
Canonical ccRCC subclonal drivers and aneuploidy burden drive specific changes to the tumor transcriptome. **A,** Illustration of the procedure to analyze the transcriptional association of a subclonal copy number alteration without the confounding effect of patient-specific factors followed in this study. **B,** Association of transcriptional changes in 50 different hallmark signatures with subclonal 9p and 14q loss in ccRCC. FDR was calculated by correcting *P*-values obtained via paired Wilcoxon tests with the Benjamini–Hochberg method between WT and mutant regions (subclonal 9p loss: 20 patients with 44 WT and 27 mutant samples; subclonal 14q loss: 26 patients with 45 WT and 41 mutant samples). Negative and positive associations are colored in blue and red, respectively. Highlighted squares indicate significant associations (FDR < 0.05). **C, ***CDKN2A* and *CDKN2B* expression (*y*-axis), quantified by log_10_ (TPM + 1), across 231 TRACERx Renal samples with different copy number status: homozygous deletions (HD, 7 samples from 4 patients), heterozygous deletion (LOH: 101 samples from 49 patients), or no loss (WT, 122 samples from 53 patients) at the 9p21 locus. *P*-values obtained via Wilcoxon test. Boxes extend from the lower to the upper quartiles, with the line inside the box representing the median, with whiskers indicating data within 1.5 times the IQR from the quartiles. **D,** Differential expression analysis of genes upstream, within, and downstream of the cGAS–STING pathway (Supplementary Table S6) between tumor samples with high and low wGII (weighted genome instability index, a measure of aneuploidy). **E** and **F,** Representative MIF images of TRACERx Renal tumor samples (**E**) with low wGII and (**F**) with high wGII. PAX8, a marker of ccRCC cells, is colored in magenta, ENPP1 is in yellow, and DAPI is in blue. **G,** Kaplan–Meier survival curves of overall survival stratified by high (top 75%) or low (bottom 75%) expression of SLC19A1 or ENPP1 and wGII (above and below median) in TRACERx Renal. For patients with more than one sample in TRACERx Renal, maximum wGII and SLC19A1 and ENPP1 expression values were considered. *P*-value is obtained by a log-rank test.

Loss of chromosomes 9p and 14q has been identified as a hallmark genomic driver of ccRCC metastasis, with the disruption of genes within 9p21 and 14q31 loci putatively mediating their phenotypic impact and selective advantage (3). However, the mechanisms by which they confer metastatic competence is unknown. Previous attempts to understand their transcriptional impact compared tumors from different patients, introducing potential patient-specific confounders (10, 11). To address this shortcoming, we compared samples from the same primary tumor with or without 9p loss and/or 14q loss (see “Methods”; Fig. 3A).

Both 9p and 14q loss correlated with changes in several transcriptional programs (Fig. 3B; Supplementary Fig. S12), albeit 9p loss correlated with more profound transcriptional dysregulation. In particular, samples with either 9p LOH or 9p21 homozygous deletion showed a marked reduction in the expression of interferon alpha and interferon gamma response genes (FDR < 0.01; Fig. 3B; Supplementary Fig. S13A–S13C). The *cis* downregulation of the 9p21 IFN I gene cluster upon copy number loss might partially underpin this association (Supplementary Fig. S13D), as mechanistically demonstrated in *in vivo* models (12). Further, 9p loss was strongly associated with increased proliferative capacity (Fig. 3B; Supplementary Fig. S12). Because 9p and 14q losses can co-occur in our cohort (Fisher’s test *P*-value < 0.001), we further interrogated the specificity of this association using single-cell RNA-seq data from 954,023 cells, curated from 8 previously published studies (see “Methods”; refs. 8, 13–19). By applying InferCNV, we detected individual cells with 9p and/or 14q loss, including 5 samples with subclonal loss of 9p and no 14q loss and 13 samples with subclonal 14q loss without 9p loss. Differential expression analysis demonstrated that 9p loss alone, and not 14q loss, consistently associates with increased proliferation (Supplementary Fig. S14A–S14F). Increased proliferation might be driven by the observed *cis* downregulation of *CDKN2B* (*P*-value = 0.01)—but not *CDKN2A* (*P*-value = 0.79)—upon 9p loss (Fig. 3C). Taken together, this might suggest a profound impact of 9p loss in the acquisition of proliferative potential and immune evasion that could rationalize its strong association with metastatic competence.

To understand how increased proliferation contributes to clinical outcomes in the context of 9p loss, we compared all 9p lost tumors with higher and lower proliferation levels, observing that those with a higher proliferation capacity had worse outcomes [overall survival HR = 3.08 (95% CI, 1.00–9.45); Supplementary Fig. S15A and S15B], whereas those with lower proliferation index had a more favorable outcome despite harboring 9p loss. We identified that 89% (16 out of 18) of these high proliferation cases bore either LOH (15/16) or homozygous deletion (1/16) of 9p. This suggests that, to acquire metastatic competence, 9p lost subclones might require the induction of specific phenotypic changes, such as increased cell proliferation, which are not uniquely driven by a second focal loss at 9p. This observation has implications for the utilization of 9p loss as a biomarker for risk stratification, suggesting its utility may be enhanced when integrated with phenotypic measurements.

Although 9p and 14q loss were the strongest individual SCNAs associated with metastasis in ccRCC (3), we previously described that, compared with those without metastatic competence, metastasizing subclones generally have higher aneuploidy burden, reflecting underlying chromosomal instability (CIN; ref. 3). Elevated CIN is expected to lead to the activation of the cGAS–STING pathway via cGAMP, which can trigger an antitumor immune response (20). It is unclear how ccRCC tumors circumvent these negative consequences of CIN.

To resolve this paradox in ccRCC, we assessed how the expression of genes related to the cGAS–STING response (Supplementary Table S6) changes with increasing aneuploidy. We controlled for patient-specific factors by comparing regions with high and low aneuploidy (measured by weighted genome instability index, wGII) within the same tumor. Unexpectedly, we noted a decrease in the expression of *CXCL11*, *CXCL10*, and *CXCL9*—chemokines usually triggered by cGAS–STING activation in healthy cells—in tumor regions with high aneuploidy (Fig. 3D), compared with those with lower aneuploidy, suggesting that canonical cGAS–STING activation is somehow bypassed. In the same differential expression analysis, we observed the overexpression of *SLC19A1* and *ENPP1* (Fig. 3D) in high aneuploidy regions and confirmed ENPP1 expression to be specific to tumor cells (expressing *PAX8*) by multiplex immunofluorescence (MIF; Fig. 3E and F; Supplementary Fig. S16A and S16B). ENPP1 is an ectonucleotidase which degrades cGAMP, a potent immune stimulator released following the detection of cytosolic DNA by cGAS. cGAMP is degraded into immune suppressor adenosine (21). SLC19A1 is a cGAMP importer, and conceivably its overexpression by ccRCC cells could lead to greater tumor reabsorption of cGAMP by tumor cells and reduction of extracellular cGAMP and its internalization by immune cells, limiting an effective antitumor response. Supporting this hypothesis, we observed that highly aneuploid tumor regions overexpressing *SLC19A1* and/or *ENPP1* show features of an immunosuppressive TME (Supplementary Fig. S17A–S17D) marked by a decrease in T effector function and an increase in myeloid inflammation.

Thus, we identified another important phenotype under selection during tumor progression and reasoned that activation of cGAS–STING suppressors in highly aneuploid ccRCC would impact clinical outcomes. Supporting this, tumors with high wGII and elevated *SLC19A1* and *ENPP1* expression showed the shortest time to progression in both TRACERx Renal and TCGA-kidney renal clear-cell carcinoma (KIRC) cohorts (Fig. 3G; Supplementary Fig. S18A–S18C). Notably, in TRACERx Renal, patients with high wGII but low *SLC19A1* and *ENPP1* expression had the most favorable prognosis, suggesting that increased CIN may not benefit ccRCC progression without effective suppression of immune activation triggered by CIN. High *SLC19A1* expression also correlated with poorer prognosis across various tumor types, complementing previously reported pan-cancer prognostic associations with *ENPP1* expression (Supplementary Fig. S19A and S19B; ref. 21).

### Evolution of the TME

Matched genetic and transcriptional data also offer the opportunity to explore the currently unclear interplay between TME and genetic evolution in ccRCC. To address this, we estimated the abundance of distinct immune cell subpopulations in the TRACERx cohort using an immune deconvolution tool (22) that was validated using histopathological estimates (Supplementary Fig. S20A and S20B).

**Figure 4.**
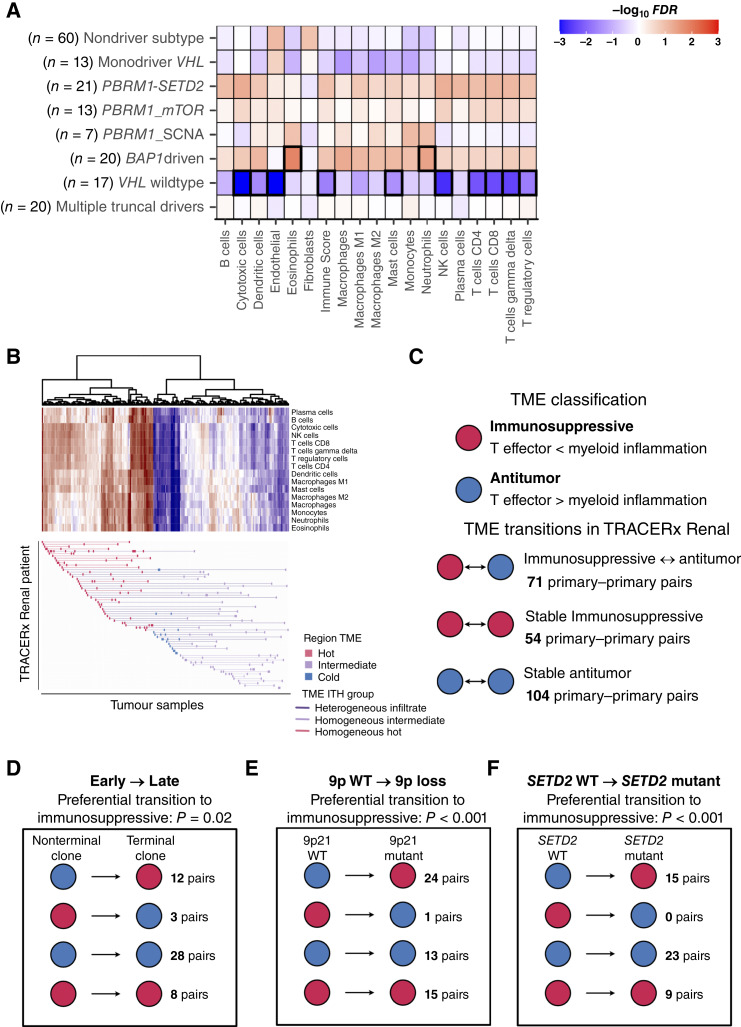
Heterogeneity of the TME in ccRCC. **A,** Association of TME cell abundance estimates from consensus TME and evolutionary trajectory of the tumor from which the sample is taken (in 171 primary tumor samples). Comparisons are one against all using a linear mixed-effects model to control for the inclusion of multiple samples from the same patient. *P*-values are corrected for multiple hypothesis testing by the Benjamini–Hochberg (FDR). Significant associations (FDR < 0.05) are highlighted with thicker borders. Negative and positive associations are shown in blue and red colors, respectively. **B,** Clustering of 213 TRACERx Renal tumor samples by their patterns of immune infiltration. In the heatmap, each row represents an immune population with its corresponding abundance determined by ConsensusTME (23); values are scaled across tumor samples. Each column represents a tumor sample, in both the heatmap and the annotation below. In the annotation, each row represents an individual TRACERx Renal patient, with their regions classified as having low, intermediate, and high levels of immune infiltration shown in blue, purple, and red, respectively. Lines connecting different regions from the same patient are colored red if all regions have high immune infiltration, dark purple if all regions have intermediate infiltration, and light purple if different regions have distinct levels of immune infiltration (no patient had more than one cold tumor region). **C,** Binary classification of the TME using the relative difference between *Z*-scores for a T effector and myeloid inflammation from Motzer and colleagues (Supplementary Fig. S22A; ref. 45). The difference between both *Z*-scores in the TRACERx Renal cohort across patients gives a normal distribution centered at 0 (Supplementary Fig. S22A); values lower than 0 (i.e., higher T effector) are classified as “antitumor TME” and “immunosuppressive” otherwise. Enrichment of TME transitions compared with background expectation (in **C**) and enriched directionality to immunosuppressive TME for (D) early → late primary–primary pairs (in which early is represented by a sample with a nonterminal clone and late by a sample containing a terminal clone), (**E**) *SETD2* WT → *SETD2*-mutant primary–primary pairs and (**F**) 9p WT → 9p loss primary–primary pairs. *P*-values for enrichment are calculated using a chi-squared test assuming an expected 0.5 probability of transition from an antitumor to an immunosuppressive TME and *vice versa*.

First, we sought to interrogate the relationship between TME composition to different evolutionary trajectories. In the context of the TRACERx Renal study, we previously described seven evolutionary subtypes—*BAP1-driven*, *PBRM1–SETD2*, *PBRM1–SCNA*, *PBRM1–mTOR*, *VHL* wild-type (WT), multiple truncal drivers, and nondriver subtype—characterized by different patterns of driver event ordering, co-occurrence, and mutual exclusivity at the clone level (see Supplementary Note S1 for a more comprehensive description of evolutionary subtypes; refs. 2, 3). Comparing the abundance of different immune cell subpopulations for samples in each evolutionary subgroup against all others (see “Methods”), we could observe that different evolutionary trajectories were characterized by distinct immune TME profiles (Fig. 4A). Tumors on a *VHL* WT trajectory exhibited a notable depletion of endothelial cells and all immune cells, particularly cytotoxic cells (e.g., NK cells and CD8^+^ T cells). By contrast, *BAP1-driven* tumors showed higher levels of immune infiltration, especially by myeloid cells, with low levels of endothelial cells. Tumors with a branched pattern of evolution, including *PBRM1–mTOR*, *PBRM1–SETD2*, and *PBRM1–SCNA* trajectories, displayed an overall increase in immune infiltration as well. However, the pattern of infiltration varied: *PBRM1–SCNA* tumors had a relatively higher abundance of myeloid cells, whereas *PBRM1–mTOR* and especially *PBRM1–SETD2* tumors had a higher relative abundance of cytotoxic cells, including CD8^+^ T cells. Taken together, this could suggest that either the TME at least partly shapes the evolutionary trajectory or traversing a certain trajectory and its somatic alterations ultimately remodels the TME.

Next, we interrogated intratumor TME heterogeneity across TRACERx Renal patients. Hierarchical clustering of RNA-seq deconvolution estimates identified three clusters of samples representing high, intermediate, and low levels of overall immune infiltration (Fig. 4B). For 32 (50%) patients, we observed variable immune infiltration patterns across primary tumor regions (Fig. 4B). Across the TRACERx Renal cohort, the range of TME ITH varied extensively (Supplementary Fig. S21A and S21B), with patients presenting higher TME ITH showing a nonsignificant trend to poorer outcomes (Supplementary Fig. S21C and S21D). This shows that TME ITH is pervasive in ccRCC.

To better understand the nature of this TME ITH, we classified the TME of individual primary tumor samples as antitumor or immunosuppressive (see “Methods”). Across all 229 pairs of samples from the same patient in the TRACERx Renal cohort, we detected 71 pairs with antitumor ↔ immunosuppressive switches, 54 pairs with conserved antitumor TME, and 104 pairs with conserved immunosuppressive TME (Fig. 4C). To identify whether these switches in the TME occur in a specific direction during tumor evolution, we tested their enrichment in 51 sample pairs in which one sample harbors a terminal clone—representing an end stage of tumor evolution at sampling—and the other contains a nonterminal clone. This analysis revealed 12 antitumor → immunosuppressive transitions (Fig. 4D), with concomitant depletion of cytotoxic infiltration (Supplementary Fig. S22A and S22B), supporting progressive immune dysfunction throughout evolution of individual ccRCCs.

In agreement with the pattern we observed of IFN downregulation in tumor regions with 9p loss, we identified 24 antitumor → immunosuppressive transitions between 40 samples pairs in which one sample had an intact 9p chromosome arm and the other sample harbored 9p loss (Fig. 4E). Finally, motivated by the observation of extensive parallel evolution of *SETD2* mutations (2) and the possibility that this is driven by a specific niche, we evaluated TME transitions co-occurring with *SETD2* mutation across 47 pairs of samples in which one sample was *SETD2* WT and the other showed a *SETD2* mutation. We observed 15 antitumor → immunosuppressive transitions from WT to mutant *SETD2* regions (Fig. 4F). This suggests that *SETD2*-mutant clones are located in specific immunosuppressive pockets of overall highly cytotoxic tumors following a *PBRM1–SETD2* trajectory (Fig. 4A). The enrichments of antitumor → immunosuppressive transitions co-occurring with 9p loss and *SETD2* alterations were corroborated by lower cytotoxic T-cell infiltration, which we did not identify for other recurrently subclonal drivers (Supplementary Fig. S22C and S22D, S23A and S23D).

### Evolution of Immune Adaptive Response

Recent studies have highlighted the utility of dissecting adaptive immune, especially T-cell, responses in ccRCC patients to identify those likely to have a favorable response to ICB (13, 23). To gain a deeper understanding of the evolution of T- and B-cell responses in treatment-naive ccRCC tumors, we employed MiXCR (24, 25) to extract T- and B-cell receptor (TCR and BCR, respectively) repertoires from bulk RNA-seq data within the TRACERx Renal cohort. This approach showed the potential to both identify major TCR clones and estimate TCR clonality in ADAPTeR (23), an independent cohort with paired bulk TCR and RNA-seq data (Supplementary Note S2). In the TRACERx Renal cohort, MiXCR identified a total of 16,582 unique TCR clonotypes (median: 55 TCR unique clonotypes per sample; Supplementary Table S7) and 159,801 unique BCR clonotypes (median: 464 unique clonotypes per sample; Supplementary Table S8) across 243 ccRCC and kidney-adjacent normal samples from 79 patients.

**Figure 5.**
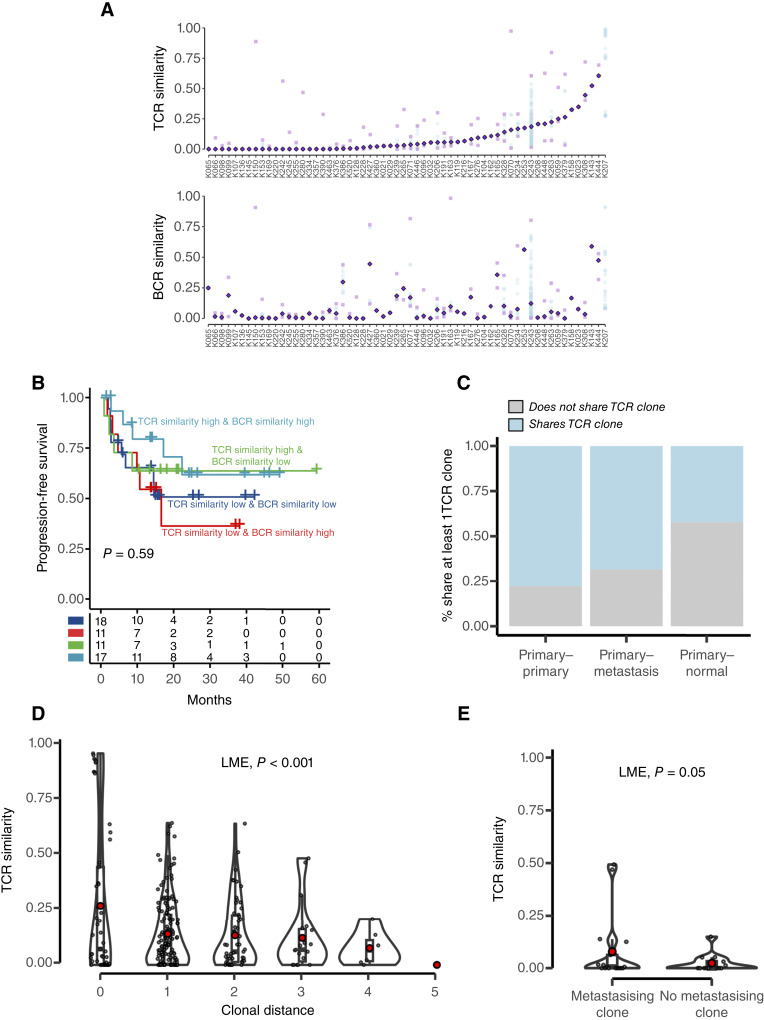
Spatial diversity of the TCR repertoire suggests heritable nature of the antigenic source in ccRCC. **A,** TCR and BCR similarity between pairs of samples across 60 TRACERx Renal patients with at least two regions sampled. Dark purple diamonds represent the median similarity (per-patient estimate of TCR/BCR similarity). Pale purple squares represent pairs with minimum and maximum similarities; blue points represent the similarity between the rest of sample pairs, when available. Patients in both plots are ordered by increasing TCR similarity. **B,** Kaplan–Meier progression-free survival curves stratified by high/low TCR and BCR similarity in TRACERx Renal. *P*-value is obtained by a log-rank test. **C,** Percentage of primary–primary, primary–metastasis, and primary–normal pairs from the same patient that share (blue) and do not share (gray) at least one TCR clone. **D,** TCR similarity between 285 pairs of primary tumor samples with increasing clonal distances, defined as the distance between the clones located in the samples of the pair (see “Methods”). **E,** TCR similarity between metastatic samples and paired primary samples depending on whether the primary region contained a metastasis-seeding clone. In (**D** and **E**), red points indicate mean values; boxes extend from the lower to the upper quartiles, with the line inside the box representing the median, with whiskers indicating data within 1.5 times the IQR from the quartiles; violins represents the distribution of values per group.

We first analyzed the similarity of the T- and B-cell repertoire across different tumor samples from the same patient. TCR and BCR repertoires varied significantly across different tumor regions, with a maximum median similarity of 60% and 61% in BCR and TCR repertoires, respectively (Fig. 5A). TCR and BCR repertoire similarity did not associate with patient prognosis individually (Supplementary Fig. S24A and S24B) or in combination (Fig. 5B).

Despite the intratumoral clonal diversity observed in T- and B-cell compartments, our findings indicate that a fraction of T- and B-cell clones present in normal and metastatic samples are shared with their corresponding primary tumor. Specifically, 68% and 83% of metastases contained at least a TCR and BCR clone also found in the primary tumor. In the adjacent normal kidney tissue, these percentages were 42% for primary tumor TCR clones and 87% for primary tumor BCR clones (Fig. 5C; Supplementary Fig. S25). Our results suggest that either identical bystander T- and B-cell clones are present in distant locations or that tumor-specific T and B cells migrate to metastatic sites, either from the primary tumor or circulation. Supporting the latter hypothesis, T-cell clones identified in primary tumor samples and matched metastatic or normal samples exhibited higher clonality within the primary tumor (Supplementary Fig. S26), consistent with their expansion during adaptive antitumor immune response.

We next sought to integrate the tumor phylogenetic structure to gain deeper insights into the drivers of T- and B-cell responses. We posited that if adaptive immune responses target heritable (neo)antigens, we would observe a progressive diversification of the TCR or BCR repertoire as the neoantigen repertoire changes throughout tumor evolution. To investigate this hypothesis, we correlated the clonal distance between pairs of tumor regions with the similarity of their TCR or BCR repertoire. We did not detect any association between clonal distance and BCR similarity (Supplementary Fig. S27), but we did find a strong association between TCR similarity and clonal distances (Fig. 5D) not fully reconciled by alternative metrics of intratumor genetic heterogeneity or burden of somatic alterations (Supplementary Fig. S28). This indicates a dynamic T-cell response propelled by heritable changes—genetic or epigenetic—accumulated during ccRCC evolution. Notably, we observe a greater TCR similarity between metastases and their clonal ancestor in the primary tumor (Fig. 5E) than the more distant clones, raising the possibility that TCR clones targeting antigens acquired during primary tumor evolution also infiltrate metastases, migrating either from the primary tumor or from the periphery.

### HERV Transcriptional Activity during ccRCC Evolution

Derepression of endogenous retroelements expression remains a potential, yet unresolved, source of immunogenicity in ccRCC (26) and additionally can contribute to tumorigenesis by different means—including tumor-promoting human endogenous retrovirus proteins, induction of changes to the host cellular gene expression, and contribution to insertional mutagenesis and/or chromosomal rearrangements (27, 28). The expression of human endogenous retroviruses (HERV) has been associated with immunotherapy responses in ccRCC (29, 30), although it is unclear whether this association is direct (23). HERV-E antigens are of particular interest, with clinical trials of adoptive transfer of chimeric antigen receptor (CAR) T cells targeting these elements underway in ccRCC patients (31). Despite this increasing interest in HERVs, it remains unknown how the expression of HERVs evolves in ccRCC and what subset of HERVs can drive antitumor responses.

To investigate transcriptional derepression of HERVs in the context of clonal evolution, we quantified HERV expression within the TRACERx Renal cohort using a previously described *de novo* assembled cancer transcriptome (32). This approach takes into account the structure of transcripts overlapping repeat elements, which allows more accurate quantification. In particular, we identified 615 transcripts that overlap annotated HERVs and other long terminal repeat (LTR) elements.

**Figure 6.**
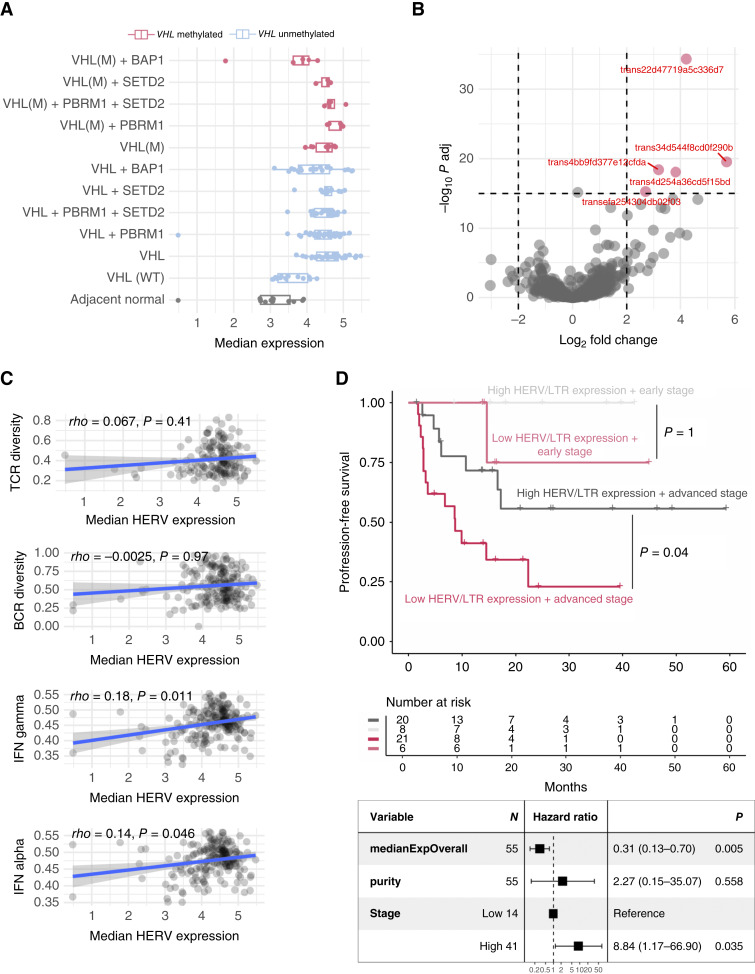
Greater overall HERV expression, strongly associated with VHL loss of function, correlates with longer progression-free survival in ccRCC. **A**, Distribution in TRACERx Renal (*n* = 243 samples) of the median expression across 615 transcripts overlapping annotated retroelements (HERVs and LTRs) by sample genotype. Boxes extend from the lower to the upper quartiles, with the line inside the box representing the median, with whiskers indicating data within 1.5 times the IQR from the quartiles. **B,** Differential expression analysis between tumor samples with VHL loss of function (methylation or mutation) and VHL WT tumor samples or adjacent normal tissue. Volcano plot jointly represents the log_2_ of fold change (*x*-axis, positive values indicate enrichment in VHL loss-of-function samples) and adjusted *P*-value (*y*-axis, using the Benjamini–Hochberg correction). **C,** Correlation (from top to bottom) between TCR diversity, BCR diversity, IFN alpha, and gamma ssGSEA scores (median per patient) and median HERV expression (median across HERVs and samples from the same patient). Each dot represents a multi-sampled patient (*n* = 60). **D,** (top) Kaplan–Meier survival curves of progression-free survival stratified by stage and high (above median) or low (below or equal to median) HERV/LTR expression. Dark pink, low HERV/LTR expression and advanced stage (stage III–IV); dark gray, high HERV/LTR expression and advanced stage (III–IV); soft pink, low HERV/LTR expression and early stage (stage I–II); soft gray, high HERV/LTR expression and early stage (stage I–II; bottom) Multivariate Cox proportional hazards model to determine the association of the median expression of HERV/LTR transcripts (median across HERVs and different samples from a patient) with progression-free survival, corrected by tumor purity and stage (high: III + IV; low: I + II)

UMAP dimensionality reduction on this set of transcripts revealed patterns of intra- and intertumor heterogeneity akin to those observed for the entire tumor transcriptome (Supplementary Fig. S29). Expression or exonization of HERV/LTRs is thought to be driven by epigenetic changes associated with cancer progression, and some HERVs are thought to be upregulated through *VHL* loss (33, 34). Given the potential effect of multiple ccRCC driver genes (*VHL*, *PBRM1*, *SETD2*, *BAP1*) on HERV/LTR expression, we tested the association of global expression of these elements with the mutation status of the said genes. Mutation or methylation of *VHL*, but not the genes mutated subsequently in ccRCC evolution, associated strongly with median HERV/LTR expression (Fig. 6A), suggesting that the induction of HERV/LTR expression is an early event in ccRCC evolution. Subsequently, HERV/LTR expression can also be modulated by copy number alterations of their genetic locus, with 6.1% showing a significant (FDR < 0.05) association with increased copy number (Supplementary Fig. S30). We further compared HERV/LTR expression in *VHL*-altered versus *VHL* WT tumors and adjacent normal tissue. Only five transcripts overlapping ERV1 and ERVL LTR elements were found significantly upregulated in *VHL* mutant or methylated tumor samples relative to WT *VHL* and normal samples (Fig. 6B; Supplementary Fig. S31A–S31E), suggesting global derepression of HERV/LTR elements rather than derepression of only a subset of retroelements upon *VHL* loss.

To investigate whether HERV expression is linked to alterations in the TME, we examined the correlation between the abundance of different cell populations and both the overall median expression of HERV/LTR overlapping transcripts and the expression of two specific HERV-E members (on Chr6q15 and Chr19q12) proposed to associate with antitumor T-cell responses in ccRCC (23, 30, 35, 36). The overall HERV/LTR expression demonstrated a positive association with increased tumor purity, confirming that a significant proportion of HERV/LTR transcripts are expressed within tumor cells. Overall HERV/LTR expression did not associate positively with any immune cell subtype and even exhibited a negative correlation with distinct myeloid cell subsets (eosinophils and neutrophils T cells and NK cells; Supplementary Fig. S32). Similarly, the expression of the two putatively immunogenic HERV-E members known to be upregulated in ccRCC was not correlated positively with any specific cell population in the TME, rather the expression of HERV-E Chr6q15 (also known as ERVE-4) was negatively correlated with cytotoxic NK cells (Supplementary Fig. S32). Moreover, overall HERV/LTR expression did not correlate with TCR or BCR diversity and only weakly correlated with the interferon (IFN) signature (Fig. 6C), suggesting that a potential contribution of HERV/LTR expression to tumor innate and adaptive immunogenicity cannot be easily inferred by joint quantification of the median expression of all ccRCC-specific HERVs.

Finally, we explored the correlation between the expression levels of HERV/LTR transcripts and clinical outcomes. Interestingly, despite its lack of association with immune metrics, elevated overall expression of HERV/LTR transcripts was associated with improved survival regardless of cancer stage (Fig. 6D). At the level of individual HERV/LTR transcripts, we did not observe any significantly associated (i.e., FDR < 0.05) with survival and only 59 and 13 associated positively and negatively, respectively, with improved outcomes with a nominal *P*-value <0.05 (Supplementary Table S9). For the top five individual HERV/LTR elements, we observed that they were embedded within or near protein-coding and noncoding genes (Supplementary Fig. S33A–S33E), instead of being independently transcribed, potentially immunogenic, HERVs. In that line, expression of immunogenic HERVs HERV-E Chr6q15 and HERV-E Chr19q12 was not significantly associated with survival (Supplementary Table S9). Taken together, this suggests that the association between higher median HERV/LTR expression and survival is not driven by any individual HERV/LTR element or by a detectable more active immune response co-occurring with global derepression of these elements. Instead, only a subset of specific HERVs might trigger immune responses, and the association between global HERV/LTR expression and survival might be attributed to tumor-intrinsic effects of dysregulated HERV/LTRs (27).

## Discussion

Through a paired genomic–transcriptomic analysis of 243 samples from 79 patients, we present the most comprehensive description to date of nongenetic evolution patterns in ccRCC. This study contextualizes a large body of prior research describing genetic ITH and evolution in this disease (1–3, 7, 9, 37).

Our findings reveal nongenetic sources of intratumor variation that a genomic-only approach would have missed. Specifically, we demonstrate that a significant portion of the transcriptional heterogeneity observed within patients cannot be accounted for by major ccRCC driver mutations or copy number alterations, consistent with recent studies in colorectal (5) and non–small cell lung cancer (6). The remaining contributors to such variation warrant further investigation, likely including noncoding genetic variation, epigenetic alterations, and phenotypic plasticity (38). The latter two, in particular, offer the potential for rapid cell state transitions, and it remains unknown how much they contribute to tumor adaptation to therapy (39) and other selective pressures in ccRCC.

Moreover, we uncovered recurrent patterns of transcriptional evolution in ccRCC, which emerge during primary clonal evolution and are, at least in part, preserved following metastatic spread. These findings suggest a convergent evolution toward specific phenotypic traits. In particular, we noted increased cell proliferation, metabolic reprogramming, and elevated expression of potential cGAS–STING repressors, *SLC19A1* and *ENPP1*, amid high aneuploidy from earlier to later clones. These dynamics might offer new potential therapeutic targets, such as ENPP1 inhibition in the context of high aneuploidy (21) or the use of CDK4/CDK6 inhibitors (40) in tumors with high proliferation and co-occurring *CDKN2B* and *CDKN2A* loss (i.e., 9p loss). Additionally, we demonstrate how transcriptional patterns can complement and refine existing genetic biomarkers in ccRCC [e.g., 9p loss, aneuploidy burden (3)] by providing context and insight into their phenotypic effects.

Our study also highlights the association between evolutionary trajectories and TME composition, suggesting a bidirectional relationship in which genetic drivers of an evolutionary path influence TME composition or the TME itself exerts selective pressures that shape driver acquisition. Supporting the first hypothesis, we observed that *VHL* WT tumors exhibited a markedly different TME, underscoring the early influence of this driver on shaping TME composition. For the second hypothesis, we found that tumors on a *PBRM1–SETD2* trajectory displayed an antitumor TME, yet *SETD2*-mutant clones tended to emerge within immunosuppressive pockets. This may indicate *SETD2* immuno-editing by an antitumor TME, aligning with our previous observations of geographically confined parallel evolution of *SETD2* mutations (2). Notably, immune infiltration patterns in *PBRM1–SETD2* tumors differed from other *PBRM1*-associated trajectories (e.g., *PBRM1–mTOR*, *PBRM1–SCNA*), suggesting that the conflicting reports regarding *PBRM1* as a biomarker for ICB response in ccRCC (41–44) may depend on its evolutionary context.

In addition, we describe the longitudinal evolution of the TME through joint analysis of tumor phylogenetic structure and TME composition. We observed a recurrent trend of progressive immune dysfunction across tumor evolution, strongly linked to frequent and recurrently late ccRCC drivers 9p loss and *SETD2* mutations. This pattern mirrors previously described immune dysfunction through clinical disease progression and raises questions on the optimal timing of ICB therapy (14). Because we described the TME using signatures of T effector function predicting ICB response (45), our analyses imply that ccRCCs would offer an improved treatment response early in their evolution, supporting the neoadjuvant paradigm. Additionally, we observed that clonal dynamics of the T-cell compartment mirrored tumor clonal structure, indicating heritability of the antigenic source in ccRCC. Such interlink persisted upon metastatic dissemination, possibly suggesting that at least a fraction of tumor-specific T-cell clones target (neo)antigens acquired during primary tumor growth and travel to distant metastases, either from the primary tumor or from blood.

One of the potential neoantigenic sources in ccRCC is HERVs. However, their overall expression did not associate neither with cytotoxic immune infiltration nor with T- and B-cell clonal structure. This suggests only a few specific HERVs are antigenic, requiring dedicated identification and further validation. However, the overall transcriptional activity of ccRCC-specific HERV/LTR overlapping transcripts was found here to associate with longer progression-free survival. Given the lack of association between median HERV/LTR expression and TME composition and interferon response, we speculate that this survival benefit might stem from tumor-intrinsic effects of dysregulated HERV/LTRs that modulate tumor growth without significantly altering immunogenicity (27).

This study has some limitations. We did not address all forms of transcriptional variation, such as alternative splicing and RNA editing. Additionally, despite the unprecedented size of our cohort, we were underpowered to investigate the transcriptional impacts of less frequent subclonal drivers. Finally, the absence of matched epigenetic data limited our ability to explore how much of the unexplained transcriptional variation is driven by epigenetic changes. Nonetheless, our findings underscore nongenetic evolution as a significant contributor to functional ITH in ccRCC, highlighting the necessity of multiomic approaches to fully understand and contextualize ccRCC evolution.

## Methods

### Data Generation and Processing

#### TRACERx Renal Cohort

The TRACERx Renal study (NCT03226886) is an ethically approved prospective cohort study (National Health Service Research Ethics Committee approval 11/LO/1996) sponsored by the Royal Marsden NHS Foundation Trust and coordinated by its Renal Unit. The study recruits patients at the following sites: Royal Marsden NHS Foundation Trust, Guy’s and St Thomas’ Hospital NHS Foundation Trust, Royal Free Hospital NHS Foundation Trust, and Western General Hospital (NHL Lothian). An extension cohort of primary and metastatic pairs was accessed under the approval of the Basque Country Research Ethics Committee, Hospital Universitario Cruces (Ref CEIC-Euskadi PI2015101).

Inclusion criteria for the study were (i) age greater or equal than 18 years, (ii) patients with histologically confirmed ccRCC, or suspected ccRCC, proceeding to surgery (nephrectomy or metastasectomy), (iii) medical and/or surgical management in accordance with national and/or local guidelines, and (iv) written informed consent to permit fresh tissue sampling, blood collection, and access to archived diagnostic material and anonymized clinical data.

Exclusion criteria for the study were (i) any concomitant medical or psychiatric problems which the investigator deemed would prevent completion of treatment or follow-up and (ii) lack of adequate tissue.

For the purpose of this study, additional eligibility criteria were applied as in a previous publication (2), including (i) confirmed histologic diagnosis of ccRCC, (ii) no familiar history of ccRCC, and (iii) no identified germline ccRCC predisposition syndrome.

Every participant in the study was assigned a study identity number (ID), with a prefix (“K”) followed by a string of three numbers. All human samples were associated with the study ID to allow integration of multiple datasets while keeping patient identity anonymous on a centralized database.

The cohort in this study is representative of patients eligible for curative or cytoreductive nephrectomy and spans multiple disease stages. In total, we sequenced 243 samples (191 primary tumor, 22 metastatic, 18 tumor thrombus, and 12 tumor-adjacent normal samples) from 79 patients (18 at stage I, 4 at stage II, 25 at stage III, and 27 at stage IV). For 14 patients, only 1 tumor sample underwent RNA-seq; the remaining 65 patients had corresponding RNA-seq data for at least 1 tumor sample (median: three samples per patient, range: 3–13). Matched full demographic, clinical, genomic, and evolutionary data for the samples under study are available in a previous TRACERx Renal publication (2, 3). Both male and female subjects were recruited to the TRACERx Renal study. Approximately 70% of patients in the subset analyzed in this study were male, consistent with the reported twice as high incidence of clear-cell renal cell carcinoma in males. Sex was not used as a covariate in any statistical analysis in this study. Patient weight was not recorded in the TRACERx Renal and hence is not included as a covariate in this study. Patient age was recorded and followed a normal distribution in this study, with a median participant age of 64 years old. A more comprehensive description of how prior results are inputted in this study is provided in Supplementary Note S1.

#### Sample Collection

Sample collection was described in previous TRACERx Renal publications (2, 3). In brief, all surgically resected samples were reviewed macroscopically by a pathologist to guide multiregional sampling. Tumors were dissected along the longest axis, and regions were sampled from the tumor slice using a 6-mm punch biopsy. Each biopsy was split along the longest axis for snap-freezing in liquid nitrogen and formalin fixation. Frozen samples were stored at −80°C.

#### RNA Extraction

DNA and RNA were co-extracted from samples in previous TRACERx Renal publications (2, 3). DNA and RNA were purified using the AllPrep DNA/RNA Mini Kit (Qiagen). Briefly, a 2-mm^3^ piece was added to a 900-µL lysis buffer and homogenized using a TissueRuptor (Qiagen) or TissueLyser (Qiagen) followed by a QIAshredder (Qiagen). Purification was performed according to the manufacturer’s recommendation either manually or on a QIAcube (Qiagen). RNA quality and yield were measured using the TapeStation and Qubit Fluorometric Quantification (Thermo Fisher Scientific).

#### Library Preparation and Sequencing

Tumor samples with sufficient RNA quality (RNA integrity score ≥5) were submitted to Oxford Genomics Centre or processed by the Advanced Sequencing Facility at the Francis Crick Institute. RNA samples were normalized to 100 ng, and libraries were constructed using the TruSeq Stranded Total RNA Library Prep Gold kit (Illumina) according to the manufacturer’s protocol. Samples were specifically depleted for both cytoplasmic (5S, 5.8S, 18S, and 28S) and mitochondrial (12S and 16S) rRNA species.

Libraries were indexed with unique dual indexes (IDT for Illumina TruSeq RNA UD Indexes, 20022371) and PCR amplified using 15 cycles. Library quality and fragment size distributions were controlled on a TapeStation 4200 instrument (Agilent). Samples were pooled before paired-end sequencing on a HiSeq 4000 or NovaSeq 6000 instrument (Illumina) with a target coverage of 50 million reads and 100-bp paired-end read length.

#### RNA-seq Alignment and Gene Expression

The quality of the RNA-seq reads was estimated using FastQC (RRID:SCR_014583, v.0.11.2). Next, fastq files were aligned to the UCSC hg19 human reference genome using STAR (RRID:SCR_004463; v.2.7.4a; ref. 46) with default parameters, yielding a BAM per tumor sample. Following this, gene expression was measured using RSEM (RRID:SCR_000262; v.1.3.3; ref. 47), which estimated count and transcript per million (TPM) expression values. Subsequently, we applied an expression filter to only keep genes with 1 TPM in at least 20% of the samples, removing a total of 6,583 genes from the original 23,299 genes, hence resulting in a final total of 16,716 genes.

Finally, we normalized raw gene counts estimated by RSEM using the variance-stabilizing transformation (VST) method from the DESeq2 package (RRID:SCR_015687; v.1.40.2; ref. 48). This method assumes a negative binomial distribution to fit a dispersion-mean distribution to the input count data, and it outputs homoscedastic—i.e., with constant variance across increasingly high mean gene count values—and library-size normalized count values. These normalized counts were used in all downstream applications, unless otherwise specified.

#### Immunohistochemistry Quantification of Macrophages and T Cells

For the TRACERx Renal cohort, we prepared tissue sections from 376 tumor samples across 24 patient cases, chosen to represent different tumor stages. Each sample was sectioned to a thickness of 4 µm and mounted on slides. We then performed single chromogen immunohistochemistry (IHC) staining for CD3 (RRID:AB_563541) and CD68 (RRID:AB_2892734) on these sections, with the specifications presented in Supplementary Table S10.

Cell quantification was performed in QuPath (RRID:SCR_018257; ref. 49). Manual segmentation of viable tumor tissue within the IHC-stained image and automated positive cell counts were performed. Cell counts are reported as the number of positive cells per µm^2^.

#### *ENPP1* MIF and Quantification

Prior to staining, two 3-µm tissue sections of tumor samples from 10 patients of the TRACERx Renal cohort identified as either high wGII high *ENPP1* (*n* = 5) or low wGII low *ENPP1* (*n* = 5) were cut. The first slides were designated for H&E staining, the following for MIF.

For the MIF panel, each primary antibody used was optimized in single-plex IHC, and all antibodies were then combined to create a six-marker multiplex panel using the Bond autostainer system (Leica Bond Rx). Briefly, sections were subjected to six sequential rounds of staining with each primary antibody followed by a secondary HRP-conjugated polymer (Leica; Novolink Polymer Detection System including antimouse postprimary and antirabbit polymer—ref RE7260-CE). Signal amplification was achieved with TSA-Opal fluorophores (Akoya; OPAL diluent ref: FP1609; Supplementary Table S11). Before the first round and between each round of staining, a heat-induced epitope retrieval step was performed (BOND epitope retrieval solution 2 ref AR9640). After the final round of antibody staining, slides were counterstained with DAPI (Thermo Fisher Scientific; ref 62248; 1/2500) and mounted with ProLong Diamond antifade mounting medium (Thermo Fisher Scientific; ref P36965).

Whole-slide scans were captured using the Akoya PhenoImager HT multispectral imaging system (former Vectra Polaris) and Vectra Polaris 1.0.13 scanning software (Akoya Biosciences) at 20× magnification (0.5 µm/pixel). Tissue regions were annotated using the Phenochart 1.1.0 software (Akoya Biosciences), and spectral unmixing was then performed using Inform 2.6.0. Delmutiplexed images were exported as 32-bit component TIFF files. Resultant image tiles for each scan were then stitched together within QuPath version 0.4.4 using a script available on the QuPath GitHub to produce a whole-slide multichannel, pyramidal OME.TIFF image for downstream digital image analysis. Tissue annotation and nuclear segmentation were achieved using the HALO AI module version 3.6.4134 and cell phenotyping using the HALO image analysis platform version 3.6.4134 (Indica Labs, Inc.). For each sample, the percentage of PAX8-positive tumor cells expressing ENPP1 was quantified.

#### Pathway Score Quantification

To estimate pathway expression in TRACERx Renal samples, we calculated ssGSEA scores (50). We used the R package GSVA (v.1.48.3; ref. 51) with TPMs as input and default parameters. The pathways analyzed in this study encompassed the 50 hallmark signatures from the Molecular Signatures Database (MSigDB) and 9 signatures that define molecular subsets of ccRCC, as previously described by Motzer and colleagues (45).

#### TME Cell-Type Estimation

To estimate the abundance of 18 nontumor cell types, along with a global measure of immune infiltration, within the TME of TRACERx Renal samples, we performed bulk RNA-seq deconvolution through ConsensusTME (22). We used the public R package (v.0.0.1.9000; ref. 22), setting “statMethod” to “ssGSEA” and “cancer” to “KIRC.”

The accuracy of ConsensusTME in estimating the abundance of specific cell types was validated by comparing its outputs to the abundance of T cells and macrophages as determined by CD3 and CD68 staining, respectively, in IHC (see “Immunohistochemistry Quantification of Macrophages and T Cells”). To assess the relationship between the two sets of estimates, we calculated Spearman’s rank correlation test for 39 samples that had both ConsensusTME estimates for T-cell abundance and CD3 IHC quantification and for another 47 samples that had ConsensusTME macrophage abundance estimates paired with CD68 IHC quantification.

#### TCR and BCR Deconvolution

TCR and BCR assembly and quantification from bulk RNA-seq were performed using MiXCR (v3.0.13; refs. 24, 25) on the pair-end RNA-seq fastq files using the following command: “mixcr analyze shotgun –species hs –starting-material rna –only-productive ${fastq1_path} ${fastq2_path} ${out_dir}.” The identified TCRɑ and TCRβ clonotypes and BCRκ and BCRλ clonotypes jointly defined TCR and BCR clones in this study. Clonotypes identified by MiXCR were imported with the repLoad function from the immunarch (RRID:SCR_023089) R package (v.0.9.0) for downstream analyses. A comprehensive benchmarking of the capacity of MiXCR to accurately identify TCR clonality and major clones is provided in Supplementary Note S2.

#### HERV/LTR Detection and Quantification

HERV and LTR overlapping transcripts specifically expressed in ccRCC were previously described (32). To overcome the limitations of available genomic HERV/LTR annotations, we herein aligned RNA-seq to a *de novo* assembled transcriptome, which enables accurate alignment and hence quantification of HERV/LTR elements (23, 32). Both total counts and TPM were quantified for each HERV/LTR, as previously described (23, 32). In total, upon this procedure, we quantified the expression of 615 ccRCC-specific transcripts overlapping known and annotated HERV/LTR elements.

#### scRNA-seq Data Integration and Harmonization

We downloaded single-cell RNA-seq data from eight different studies (8, 13–19). The raw or processed expression matrix files were downloaded according to the original publications. Seurat objects were generated with the expression matrix using the Seurat (RRID:SCR_016341; ref. 52) R package (v.5.1.0) with available sample metadata.

To ensure the exclusion of low-quality cells and doublets, we removed cells in which mitochondrial reads constituted more than 20% of the total reads. Cells with fewer than 200 RNA features or those exceeding the mean RNA feature count by more than 2.5 times the standard deviation for their respective study were also excluded. Additionally, we removed genes associated with mitochondria, ribosomes, and MALAT1.

The Seurat objects from different studies, encompassing a total of 954,023 cells, were integrated with Harmony. We then performed principal component analysis (PCA) and UMAP for dimensional reduction (using dimensions 1–30) via RunPCA and RunUMAP, respectively. *PTPRC* gene expression was used to identify the immune cell clusters. Subsequently, the nonimmune cluster was subset and re-clustered with the steps above. We identified the cluster of normal proximal tubular cells by their expression of the marker genes *CUBN* and *PDZK1IP1*.

Finally, to identify ccRCC tumor cells, marked by the pathognomic loss of 3p, we applied InferCNV (RRID:SCR_021140; v.1.3.3) with default setting on the nonimmune clusters, using the normal proximal tubular cells as reference. We calculated the mean residual expression value for genes in the region chr3:8,100,001 to 11,600,000 for each cell within the InferCNV object. Based on the distribution of these values, we set a threshold of less than −0.05 (indicating loss of chr3p21) to classify cells as tumor cells. In total, across all the studies, we identified a total of 50,711 tumor cells following this procedure.

#### TCGA RNA-seq Data

Raw RNA-seq counts from 538 TCGA-KIRC primary tumors were downloaded using the TCGAbiolinks (RRID:SCR_017683) package (v.2.20.0; ref. 53), with the following parameters: data.category = “Transcriptome Profiling,” data.type = “Gene Expression Quantification,” experimental.strategy = “RNA-Seq,” and workflow.type = “STAR - Counts.” Downloaded gene expression counts were normalized, as in the TRACERx Renal RNA-seq, using VST. The associated clinical information was obtained from the Supplementary Data provided by Liu and colleagues (54).

We followed the same procedure to download and process clinical and RNA-seq data from 17 additional tumor types where Li and colleagues (21) previously tested the association of ENPP1 expression with clinical outcome: BLCA, bladder urothelial carcinoma; BRCA, breast cancer; CESC, cervical squamous cell carcinoma and endocervical adenocarcinoma; COAD, colon adenocarcinoma; ESCA, esophageal carcinoma; HSCN, head and neck squamous cell carcinoma; KIRC, kidney renal clear-cell carcinoma; LUAD, lung adenocarcinoma; LUSC, lung squamous cell carcinoma; OVCA, ovarian serous cystadenocarcinoma; PAAD, pancreatic adenocarcinoma; SARC, sarcoma; SKCM, skin cutaneous melanoma; STAD, stomach adenocarcinoma; THCA, thyroid carcinoma; THYM, thymoma; UCEC, uterine corpus endometrial carcinoma.

### Analysis

#### UMAP

To visualize transcriptional variation in the TRACERx Renal cohort, UMAP analysis was performed on all the 231 tumor samples in the TRACERx Renal cohort. UMAP was performed on VST counts using the umap (v.0.2.10) package in R with default parameters.

#### Quantification of Transcriptional and TME Distance

We estimated transcriptional distance between two tumor regions from the same patient using the approach described by Martínez-Ruiz and colleagues (6). Briefly, we first calculated the top 500 most variable genes in the entire TRACERx Renal cohort by ranking the genes according to the variance of VST counts across the cohort. Next, we used these 500 genes to calculate Pearson’s correlation between VST counts of these genes in a pair of samples, using the function dcor() from the energy (v.1.7-11) R package. This correlation (*r*) reflects the similarity in the transcriptional pattern of both samples. To transform this similarity to a distance (*d*), we defined the distance as *d* = 1−*r*, where r is the correlation previously quantified. We followed this procedure to estimate the transcriptional distances between all the pairs of samples from the same patient in our cohort. To assess the impact of restricting the calculation of transcriptional distance to the top 500 genes, we repeated this procedure using progressively larger sets of genes (top 1,000, 2,500, 5,000, 10,000, 12,500, and 15,000 most variable genes). We then correlated the scores from each of these sets with those derived from the top 500 most variable genes (Supplementary Fig. S3B).

We quantified TME distance following a similar approach. In this case, the input to these calculations was the abundance of all nontumor cell populations previously quantified using ConsensusTME (22). Here, the similarity between the TME of two samples was estimated with the cosine similarity (*c*) between the TME abundance vectors of two samples. Cosine similarity (*c*) was estimated using the function cosine() from the lsa (v.0.73.3) R package. The final distance (*d*) between a pair of samples was defined as *d* = 1−*c*, where c is the cosine similarity between the TME of both samples of the pair.

#### Comparison of Transcriptional Intratumor and Intertumor Heterogeneity

To compare the relative difference in the magnitude of transcriptional intertumor heterogeneity relative to ITH, we calculated transcriptional distance between (i) pairs of samples from the same patient (representing transcriptional ITH) and (ii) pairs of samples from distinct patients (representing transcriptional intertumor heterogeneity). To estimate the mean and 95% confidence interval of the coefficient between transcriptional intertumor heterogeneity and ITH, we performed bootstrapping with 1,000 iterations by (i) sampling with replacement from the pairs from the same and from different patients, (ii) estimating transcriptional intratumor and intertumor heterogeneity as the mean distance between samples from pairs of the same and different patients, respectively, and (iii) dividing both estimates to obtain the distribution of the desired coefficient. The limits of the 95% confidence interval were next defined as the 2.5% and 97.5% quantiles of the 1,000 estimates of the coefficient.

#### Transcriptional and TME I-TED

To obtain a final estimate of global transcriptional ITH per patient, we again emulated the approach previously followed by Martínez-Ruiz and colleagues (6). This metric was chosen because of its ability to provide an estimation of global transcriptional ITH that is independent of the number of samples available for each patient. This is crucial in our cohort given variations in the total number of samples per patient.

For each patient with more than one primary tumor RNA-seq sample available in the study, we calculated all possible transcriptional distances as described in the previous section (see “Quantification of Transcriptional and TME Distance”). Next we calculated for each patient the median of the transcriptional distances between all their primary–primary sample pairs. This median value is defined as I-TED and reflects the global transcriptional ITH within a patient’s tumor.

We obtained a per-patient estimate of TME ITH (TME I-TED) from pairwise TME distances—calculated as above described (see “Quantification of Transcriptional and TME Distance”)—in the same manner.

#### Correlates of Transcriptional I-TED

We performed a multivariable linear regression to identify the proportion of the variance in transcriptional I-TED scores in TRACERx Renal explained by nine previously published clinical and genetic variables. The variables included in this analysis, and their rationale for inclusion, were as follows:

#### Potential Confounders

- *Purity ITH*: Differences in purity across tumor samples could introduce changes in the expression profile given the different admixture of the tumor and nontumor compartment—with different expression profiles—in distinct samples.
- *Tumor size*: Tumors with higher size might harbor a greater diversity of tumor cell subpopulations and/or microenvironments that, upon sampling, could ultimately result in high variation in the transcriptional profile between different samples from the same patient.
- *Number of regions sampled*: Alternative estimates of transcriptional ITH, including median variance in expression across genes in distinct samples from the same patient, are influenced by the total number of samples collected from each tumor. I-TED has the advantage of being robust to the total number of samples available for each patient and was specifically selected for this advantage, which avoids an artificial overestimation of ITH in patients with more available samples. This variable is included in the model to ensure I-TED properly controls this potential confounder in the TRACERx Renal cohort.

#### Suspected Biological Determinants of Transcriptional Variation

- *Overall degree of mutation ITH* (genetic ITH): Mutations that differ across regions of a tumor could imprint a different phenotype and/or transcriptome to the regions in which they are exclusively located. We therefore reasoned that genetic ITH could correlate with transcriptional ITH or I-TED.
- *Copy number heterogeneity*: Variations in gene dosage are known to underpin changes in gene expression (55). Therefore, we hypothesized that copy number heterogeneity across different samples from a patient—summarized per patient as the fraction of the genome subjected to subclonal copy number alterations—should correlate with transcriptional ITH.
- *Clinical stage*: Genetic ITH has been recurrently associated with disease aggressiveness and clinical outcomes, and intratumor diversification occurs as a result of tumor evolution. We reasoned that transcriptional ITH (and hence I-TED) could be representing functional ITH, which could be associated with patient outcomes and disease stage at sampling.

#### Suspected Correlates of Greater Phenotypic Heterogeneity

- *Subclonal mutations in any ccRCC epigenetic driver*: Epigenetic regulation is a fundamental layer of gene expression control. We reasoned that the frequent epigenetic drivers observed in ccRCC (*ARID1A*, *KDM5C*, *SETD2*, *BAP1*, and *PBRM1*) in TRACERx Renal (2) could cause profound changes in the epigenome, which would result in broad differences in the transcriptome. Therefore, patients with subclonal variation in the mutational status in any of these drivers might display greater transcriptional ITH.
- *9p loss and 14q loss*: Losses in 9p and 14q were nominated as drivers of metastatic competence in the prior TRACERx Renal (3) and were recurrently found as subclonal alterations. We hypothesized that the acquisition of metastatic competence could imply a remarkable change in the tumor phenotype and/or transcriptome. Therefore, we hypothesized that these events, as genetic drivers of metastatic competence, could cause and/or correlate with profound transcriptional changes.

In the first step, a multivariable linear regression analysis—using lm function from base R—was conducted to assess the relationship between these variables and I-TED. Subsequently, the significance of each variable’s contribution to explaining the variance in scores was evaluated using the anova function from base R.

Finally, to provide further context to the association of subclonal loss of 9p with increased transcriptional I-TED, we fitted the same multivariable linear regression model iteratively including only 1 of the 14 previously described driver CNAs in the TRACERx Renal study (i.e., 9p loss, 14q loss, 3p loss, 1q gain, 7q gain, 6q loss, 12p loss, 2q gain, 8q gain, 5q gain, 1p loss, 4q loss, 20q gain, and 8p loss).

#### Gene-Specific Linear Regression Framework

To investigate the correlates of gene-specific changes in expression between samples from the same patient, we implemented a gene-specific linear regression framework. For each primary–primary pair of samples in the cohort, we calculated the following: (i) the difference in variance-stabilizing transofmations (VST) counts and (ii) the difference in copy number for each of 14,120 genes (genes passing expression filtering criteria, mapped to autosomes and having available copy number information in TRACERx Renal), (iii) the difference in number of mutations in epigenetic drivers *SETD2*, *PBRM1*, *ARID1A*, *KDM5C*, and *BAP1* (positive for higher number of mutated epigenetic drivers in the second sample, negative otherwise), (iv) the difference in 9p loss status (+1 for loss in the second sample and not the first, 0 for same status, −1 for loss in the first sample and not the second), and (v) the difference in WGD status (1 if the second sample was WGD and the first was not, 0 if the same status, −1 if the first sample was WGD and the second was not).

Upon calculation of these values, we fitted a linear mixed-effects model to differences in gene expression across 14,120 in TRACERx Renal primary–primary pairs, using as covariates (i) the difference in gene CN, (ii) the difference in number of mutations in epigenetic drivers, and (iii) the difference in 9p loss status. Additionally, binary and continuous values were randomly drawn and inputted as covariates in the model to obtain a background expectation. The patient ID for each primary–primary pair and WGD status were used as random covariates in the model to correct for the inclusion of several pairs from the same patient and for the potentially different association of expression and gene absolute copy number depending on background WGD status. To avoid collinearity between difference in 9p loss status and gene-specific copy number difference for genes located in the 9p locus, we did not include the difference in copy number when the gene resided in the chromosome arm 9p.

To evaluate the changes in gene expression associated with the sample type (normal or metastasis), we fitted the same linear regression model but included a new variable describing the type of pair. To evaluate the changes in gene expression from normal to primary, we performed the same analysis including primary–primary and primary–normal pairs, and this variable took a value of 1 if the second sample was normal and the first was not, 0 if both samples were of the same type, and −1 if the first sample was normal and the second was primary. To evaluate the changes in gene expression from primary to metastasis, we included primary–primary, primary–metastasis, and metastasis–metastasis pairs, and this variable was defined as 1 if the second sample was metastasis and the first was primary, 0 if both samples were of the same type, and −1 if the first sample was a metastasis and the second was primary.

#### Quantification of TCR and BCR Similarity

To assess the similarities between the TCR and BCR repertoires between pairs of samples in the TRACERx Renal cohort, the Morisita–Horn index was used, which is a measure of community similarity that accounts for both the abundance and the diversity of species (or in this case, TCR or BCR clonotypes). The Morisita–Horn index was calculated using the repOverlap from the immunarch (RRID:SCR_023089; v.0.9.0) R package, with the method parameter set to “morisita.”

#### Quantification of Clonal Distance

The separation between two clones in a tumor phylogenetic tree (defined as clonal distance in this study) informs about the extent to which they have evolved independently; clones very far apart in a phylogenetic tree are likely to have independently acquired genetic and epigenetic changes for a longer time than two clones proximally located in the phylogenetic tree. We define the “clonal distance” between two tumor samples as the distance in the phylogenetic tree between the tumor clones they harbor.

In this study, we operationalize “clonal distance” as the separation between two tumor samples in the phylogenetic tree, reflecting the evolutionary divergence between the tumor clones they harbor. To accomplish this, we leveraged the phylogenetic tree reconstruction from a prior TRACERx Renal study (2). Initially, we identified the tumor clones present in each corresponding tumor sample. Subsequently, we quantified the distance in the phylogenetic tree between the tumor clone in one sample and its counterpart in the other. This distance was determined in practice as the number of edges required to traverse from one clone to the other. Therefore, tumor regions harboring the same tumor clone are a special instance in which the clonal distance is zero. For regions hosting multiple tumor clones, we computed the distance individually for each clone in one sample to all clones in the other. The clonal distance between the two samples was then summarized as the minimum observed distance.

#### Associations of Clonal Distance with Matched Transcriptional or Microenvironmental Distance

To understand the coevolution of the tumor transcriptome or TME with the tumor genome, we first used the definitions provided in prior sections to estimate the (i) clonal distance (see “Quantification of Clonal Distance”), (ii) transcriptional distance (see “Quantification of Transcriptional and TME Distance”), (iii) TME distance (see “Quantification of Transcriptional and TME Distance”), and (iv) TCR and BCR similarity (see “Quantification of TCR and BCR Similarity”) between all the possible pairs of samples from the same patient in TRACERx Renal. The different types of distances were all tested for an association with clonal distance. We used linear mixed-effects models to test for such an association while controlling for the inclusion of multiple pairs of samples from the same patient in the analysis. Differences in purity between samples were included as a covariate in the model.

#### Comparison of Transcriptional Distances between Primary–Normal, Primary–Primary, and Primary–Metastasis Pairs

We compared the transcriptional distances between different types of pairs of samples: primary–normal, primary–primary, and primary–metastasis. Transcriptional distances were calculated as described above (see “Quantification of Transcriptional and TME Distance”). For the comparison between primary–primary and primary–normal transcriptional distances, only patients with at least one primary–primary and one primary–normal pair of samples were included. When multiple types of pairs were available for one patient, we summarized the transcriptional distance between samples of each type of pair to the observed maximum. After that, the changes in the transcriptional distances between primary–primary and primary–normal pairs of samples were compared using a paired Wilcoxon test. The same approach was used to compare primary–primary and primary–metastasis pairs, again including only patients with at least one primary–primary and one primary–metastasis pair of samples.

#### Analysis of Expression Changes from Matched Normal to Primary and Primary to Metastasis

For each patient, we compared ssGSEA scores for 50 hallmark signatures from the MSigDB between matched normal–primary or primary–metastasis pairs. Only patients with normal–primary and primary–metastasis pairs were included in these analyses, respectively. For patients with more than one normal or primary or metastasis sample, we summarized ssGSEA scores for each pathway and the type of sample by taking the average across all the samples of the same type. Paired normal–primary and primary–metastasis ssGSEA scores were then compared using a paired Wilcoxon two-sided test.

#### Comparison of Transcriptional and Microenvironmental Distances between Metastases and Matched Seeding and Nonseeding Primary Regions

To investigate whether transcriptional and/or microenvironmental patterns acquired during genetic coevolution in the primary tumor persist after metastatic dissemination, we examined whether the similarity between matched primary and metastatic samples was higher when the primary region harbored a clone that disseminated to the metastatic site. To this end, across patients with RNA-sequenced primary and metastasis samples, we calculated the distance in previously derived phylogenetic trees in TRACERx Renal from the latest (sub)clone(s) in the primary tumor sample to the MRCA of the matched metastasis. Primary tumor samples containing the tumor (sub)clone spreading to the metastasis were defined as “seeding” and otherwise as “nonseeding.”

Utilizing this classification, we compared the transcriptional distance and TCR similarity between primary seeding metastasis pairs and nonseeding primary–metastasis pairs. Both comparisons were conducted using linear mixed-effects models to adjust for the inclusion of multiple pairs of samples from the same patient. Differences in purity between pairs of samples were included as a covariate in the model.

#### Gene Expression and TME Assignment to Individual Tumor Clones

Transcriptional data offer sample-level gene expression profiles and deconvoluted TME composition. To translate this information at the tumor sample to the clone level, we implemented the following approach:

1) Exclude polyclonal tumor regions: Tumor regions harboring multiple tumor clones were excluded from further analysis to ensure that each remaining region contains only a single clone.
2) Assign gene expression profile and TME composition to each clone:
  - For clones present in only one monoclonal tumor sample, their gene expression profile and TME composition were set according to the observed values in the respective tumor sample in which the clone is located.
  - For clones present in multiple monoclonal tumor samples, the gene expression profile and TME composition were determined as the mean across all tumor samples in which the clone is present.

#### Association of Pathway Expression with Distance to the MRCA

To investigate recurrent gene expression changes occurring from earlier to later clones, we compared the expression of different pathways assigned to a given clone with a metric approximating its degree of clonal evolution. Pathway expression was quantified using ssGSEA scores, which were transformed from per-region estimates to per-clone estimates following the procedure outlined in “Gene Expression and TME Assignment to Individual Tumor Clones.”

The degree of clonal evolution was approximated by the distance of a clone to the MRCA of the tumor population, calculated as the number of edges separating a clone from the MRCA. This comparison allowed us to assess the transcriptional profile differences between more recent clones (distant from the MRCA) and ancient clones (closer to the MRCA).

To evaluate the association between ssGSEA scores and clonal age across the 50 MSigDB hallmark gene sets, we utilized linear mixed-effects models. This statistical approach allowed us to control for the inclusion of multiple samples from the same patient, thereby minimizing potential confounding effects. The purity of the samples containing a tumor subclone (average purity if multiple samples contained the same subclone) was included as a covariate in the model.

Subsequently, *t*-values and *P*-values resulting from the association analysis were aggregated by MSigDB functional group, as defined by Martínez-Ruiz and colleagues (6). *P*-values were then adjusted for multiple hypothesis testing using the Benjamini–Hochberg method.

#### Association between Transcriptional Distance to Matched Tumor-Adjacent Normal Samples and Distance to MRCA

To analyze potentially increased transcriptional distance from a tumor sample to matched normal kidney tissue putatively driven by clonal evolution, we compared the transcriptional distance between a primary tumor sample and a matched tumor-adjacent normal sample with the clonal distance from the tumor to its MRCA, calculated as described in “Association of Pathway Expression with Distance to the MRCA.” Resulting transcriptional distances and distances to the tumor MRCA were compared using a linear mixed-effects model to control for the inclusion of multiple samples from the same patient in the analysis.

#### Association of Subclonal Driver Alterations and Changes in Gene Expression

To identify the recurrent changes in gene expression co-occurring with the acquisition of subclonal copy number alterations associated with metastasis (9p and 14q loss) while controlling for inter-patient variation, we used the procedure described in Fig. 4A. First, we identified tumors with subclonal alterations for a given gene copy number alteration. Next, we obtained an estimate of the expression of a given pathway in samples (i) with and (ii) without the driver alteration by taking the average ssGSEA score of given pathway in both groups of samples. Statistical significance of the within-patient differences in the scores between WT and mutant regions observed in the TRACERx Renal cohort was obtained using a paired Wilcoxon test. This analysis was performed for the 50 hallmark signatures from the MSigDB. The resulting *P*-values were corrected for multiple hypothesis testing using the Benjamini–Hochberg method. The logarithm in base 10 of the resulting FDR values was plotted, using different colors for positive (red) and negative (blue) associations.

#### Differential Expression Analysis of 9p Loss and 14q Loss at Single Cells against WT Cells

To evaluate the transcriptional impact of 9p loss and 14q loss upon recent acquisition at the single-cell level, we downloaded and processed single-cell RNA-seq data from eight different studies (8, 13–19), followed by the identification of ccRCC tumor cells (marked by pathognomic 3p loss; see “scRNA-Seq Data Integration and Harmonization”).

To stratify the tumor cells with different 9p and 14q copy number alteration status, we used InferCNV (RRID:SCR_021140; v.1.3.3). The mean residual expression value of genes across the chr9:19,900,001 to 25,600,000 and chr14:19,900,001 to 33,200,000 for each tumor cell from the InferCNV object was calculated. Based on these distributions, thresholds of −0.05 and 0.05 were determined to identify copy number loss and gain, respectively. Cells with a value between −0.05 and 0.05 were classified as copy number neutral for the locus.

Upon identification of cells with 9p and 14q loss, we performed pseudobulk differential expression analysis using the function FindMarkers from the Seurat (RRID:SCR_016341) R package, with “test.use” set to DESeq2. Because the aim of the analysis was to identify the immediate transcriptional impact of the loss of these chromosomal regions, we limited the analysis to only cells from patients with between 10% and 90% of cells and at least 10 total cells with the copy number loss of the locus of interest. Obtained per-gene estimates of differential expression associated with both 9p and 14q loss were used as input to a gene set enrichment analysis of the 50 hallmark signatures from the MSigDB, performed with fgsea R package (v.1.26.0).

#### Differential Expression Analysis of cGAS–STING Genes by Aneuploidy

To evaluate the changes in expression of genes involved in the cGAS–STING response (Supplementary Table S6) associated with increased aneuploidy, we performed differential expression analysis by weighted genome instability index of a tumor sample (wGII). We applied DESeq2 (v.1.40.2) to raw, unnormalized counts using wGII (as a continuous numerical variable) as the variable under study and purity as a covariate, to limit its effect in the observed transcriptional output.

#### Evaluation of Patterns of TME ITH

To interrogate patterns of TME ITH, we performed hierarchical clustering of the abundance of 16 different nontumor cell populations estimated by ConsensusTME (22). Abundance scores for all the nontumor cell populations were scaled across all samples included in the analysis. Hierarchical clustering was performed using the function heatmap() from the R package ComplexHeatmap (RRID:SCR_017270; v.2.16.0), using “manhattan” as the clustering distance of the column and “complete” as the clustering method for rows and columns. Upon visual inspection, we identified three different groups and hence set “column_split” to 3.

#### Comparison of TME Composition between Different Evolutionary Trajectories

We investigated the potential correlation between the abundance of specific cell types within the TME and the evolutionary trajectory in which they are situated.

Our analysis leveraged cell abundance estimates obtained through ConsensusTME (22) for each sample, along with patient-level evolutionary trajectory annotations as previously delineated (2).

To quantify this association, we employed a linear mixed-effects model. In this model, we compared the cell abundance in samples following one trajectory against the cell abundance in samples classified within any of the other possible trajectories. Additionally, we controlled for the inclusion of multiple samples from the same patient to mitigate potential confounding effects.

The resulting *P*-values from this analysis were log transformed (base 10) and plotted, with positive associations depicted in red and negative associations in blue.

#### Per-Patient Changes in the TME

To uncover shifts in the TME composition between different primary tumor regions within a patient, we first labeled the nature of the TME into two categories: immunosuppressive and antitumor TME. Immunosuppressive TME was defined as higher mean-scaled ssGSEA scores (*Z*-scores) for the myeloid inflammation signature described by Motzer and colleagues (45), compared with mean-scaled scores (*Z*-scores) for the T-cell effector signature described by the same authors; otherwise the TME of the sample was classified as antitumor.

Second, we described the number of pairs of samples from the same patient in which the nature of the TME either remained stable (stable immunosuppressive or stable antitumor) or shifted from one category to the other (antitumor ↔ immunosuppressive).

Finally, we investigated how frequently the TME shifted during (i) progression through the phylogenetic tree (*nonterminal clones* vs. *terminal clones*), (ii) acquisition of mutations in *SETD2*, and (iii) loss of 9p. *Nonterminal clones* were defined as clones located internally in the patient phylogenetic tree; *terminal clones* were the leaves of the phylogenetic tree. TME assignment to these clones was performed as described in “Gene Expression and TME Assignment to Individual Tumor Clones.” For these comparisons, the enrichment for antitumor → immunosuppressive transitions was evaluated by comparing the number of antitumor → immunosuppressive and immunosuppressive → antitumor transitions to a background expectation of 50% chance for each type of transition using a chi-squared test.

To ensure that the identification of these transitions was not vulnerable to the specific classification of the nature of the TME, we performed paired comparisons of ssGSEA scores for the T-cell effector signature from Motzer and colleagues (45). For these analyses, only patients with at least one sample categorized into each of the compared groups were included. If multiple samples were available for one patient, the mean ssGSEA was taken as the per-patient score.

#### Patterns of Variation of HERV Expression, Link to TME, and Adaptive Immune Response

To estimate the patterns of intratumor and intertumor HERV transcriptional heterogeneity, UMAP analysis was performed on 216 tumor samples in the TRACERx Renal cohort, limiting the analysis to the 615 transcripts identified earlier (see “HERV/LTR Detection and Quantification”). UMAP was performed using the umap (v.0.2.10) package in R with default parameters. VST counts were obtained by applying DESeq2 (v.1.40.2) to raw, unnormalized HERV/LTR counts.

To estimate the differences in expression between VHL-mutated or methylated tumor samples and WT VHL tumor samples or adjacent normal tissue, we applied DESeq2 (v.1.40.2) to raw, unnormalized HERV/LTR counts.

To estimate the association between HERV/LTR and the composition of the TME, we applied Spearman’s rank correlation test between paired ConsensusTME microenvironment estimates and HERV expression using the function rcorr from the Hmisc R package (v.5.1-1), plotted using the corrplot R package (v.0.92). We applied the same procedure to correlate HERV/LTR expression to BCR and TCR diversity, estimated through Gini’s coefficient.

#### Association between Copy Number Alterations and Changes in HERV Expression

To evaluate whether the expression of HERVs depends on the genomic copy number status of its locus, we performed an expression quantitative trait loci (eQTL) analysis, in which we could associate the expression of each HERV to the CN of its locus. Because the association with CN becomes preferentially detectable when HERVs are consistently expressed across tumor samples, we filtered out in this analysis 207 of 615 ccRCC-specific HERVs (32) that were expressed in less than 70% of tumor samples. We extracted the copy number status of each sample in the genomic coordinates of a given HERV and normalized HERV counts using a VST (as done in the results presented in Fig. 6). We then used a linear mixed-effects model to correlate the expression of a HERV by three fixed covariates—(i) copy number of the genomic locus it resides in, (ii) the purity of a tumor sample, and (iii) the *VHL* (WT or mutated/methylated) status of a sample—and patient of origin of the sample as a random covariate. Purity and *VHL* mutational/methylation status were included as we or others previously described their impact on HERV expression.

#### Survival Analyses

Kaplan–Meier curves were used to illustrate the differences in PFS between different groups. *P*-values indicated in Kaplan–Meier curves were calculated using a log-rank test. Estimation and representation of Kaplan–Meier curves were performed using functions survfit and ggsurvplot from the survival (v.3.5-7) and survminer (v.0.4.9) R packages. To correct for covariates in different analyses, we applied Cox proportional hazards regression models using the function coxph from the survival R package (v.3.5-7).

#### Statistical Analysis

For all the statistical tests, the significance level was fixed at 0.05 and *P*-values were corrected by *post hoc* Benjamini–Hochberg (FDR) correction when necessary. Sample sizes (*n*) are specified either by showing all individual points or by an indication in figure legends. No power calculations were run to predetermine the sample size in any of the experiments. All genetic and molecular information derived from sequencing of TRACERx Renal samples were obtained after clinical data collection. Hence, clinical collection, but not data analysis, was blindly performed.

### Data Availability

Multiregional DNA sequencing data on TRACERx Renal 101 patient cohort were published in a previous report, and corresponding raw data are available in the European Genome-phenome Archive (EGA) under the accession number EGAS00001002793. Data availability for the single-cell RNA-seq data used in this study is detailed in the corresponding source publications (8, 13–19). Processed RNA-seq data are provided in the GitHub repository associated with this publication at https://github.com/sanroman-24/tx100_rna_2024. Access to the raw RNA-seq data is controlled by the TRACERx Renal Data Access Committee. All other data supporting the findings of this study are publicly available without restrictions. The source code and data for reproducing analyses and figures are available at https://github.com/sanroman-24/tx100_rna_2024

## Supplementary Material

Supplementary Note 1 Inputting information from previous TRACERx Renal studies

Supplementary Note 2 Evaluation of the potential to reconstruct TCR repertiore from bulk RNA-Sequencing data

Supplementary Table 1 Supplementary Table 1. Variance in gene expression for 16,716 genes passing expression filtering criteria in the TRACERx Renal study

Supplementary Table 2 Supplementary Table 2. Changes in ssGSEA scores from normal kidney tissue to primary ccRCC tumor in normal-primary pairs.

Supplementary Table 3 Supplementary Table 3. Changes in ssGSEA scores from primary to metastasis in primary-metastasis pairs.

Supplementary Table 4 Supplementary Table 4. 50 MSigDB hallmark signatures and corresponding categories.

Supplementary Table 5 Supplementary Table 5. Changes in ssGSEA scores from early to late clones.

Supplementary Table 6 Supplementary Table 6. Differential expression analysis by wGII of genes involved in cGAS-STING pathway.

Supplementary Table 7 Supplementary Table 7. TCR clones identified applying miXCR to bulk RNA-Sequencing data across 243 ccRCC and kidney-adjacent normal samples in TRACERx Renal.

Supplementary Table 8 Supplementary Table 8. BCR clones identified applying miXCR to bulk RNA-Sequencing data across 243 ccRCC and kidney-adjacent normal samples in TRACERx Renal.

Supplementary Table 9 Supplementary Table 9. Univariate Cox regression results for progression-free survival in the TRACERx Renal cohort based on the expression of each of 613 HERV/LTR elements.

Supplementary Table 10 Supplementary Table 10. Antibody and incubation procedure for immunohistochemistry (IHC) of CD3 and CD68.

Supplementary Table 11 Supplementary Table 11. Antibody / Opal combinations, dilutions and positions used for 6 biomarker immunofluorescence panel staining. RTU = Ready to Use.

Supplementary Figures 1-33 All Supplementary Figures are provided in PDF format with the corresponding legend after each figure. Supplementary Figure 1. Genetic and clinical composition of the TRACERx Renal cohort Supplementary Figure 2. Comparison between transcriptional inter and intratumour heterogeneity Supplementary Figure 3. Representation of I-TED to measure transcriptional intratumour heterogeneity and robustness analysis Supplementary Figure 4. Transcriptional ITH is not associated with poorer outcomes in ccRCC Supplementary Figure 5. Subclonal 9p loss is the subclonal somatic copy-number alteration with the greatest association with transcriptional ITH Supplementary Figure 6. Variance in transcriptional intratumour heterogeneity explained by major clinico-genomic covariates Supplementary Figure 7. Association betweeen transcriptional distance between matched tumour-normal pairs of samples and distance from the tumour sample to the most-recent common ancestor (MRCA) Supplementary Figure 8. Schematic representation of the transcriptional and clonal distance calculation in this study. Supplementary Figure 9. Association between transcriptional and clonal distance between primary-metastasis pairs of samples Supplementary Figure 10. Similarity between primary and matched metastases for different transcriptional signatures depending on the detection of a seeding clone in the primary tumour region Supplementary Figure 11. Schematic representation of the assignment of gene expression to clones Supplementary Figure 12. Heatmap with the results of differential expression between 9p or 14q loss samples and matched 9p and 14q wild-type samples Supplementary Figure 13. Association between loss of 9p and expression of IFN I cluster genes. Supplementary Figure 14. Evaluation of changes in gene expression between 9p wild-type and 9p loss clones in published single-cell RNA-sequencing data Supplementary Figure 15. Survival in patients with high or low proliferation scores and 9p loss or 9p wild-type status Supplementary Figure 16. Validation of ENPP1 overexpression observed in RNA-Sequencing with multiplex immunofluorescence Supplementary Figure 17. Association between TME composition and expression of ENPP1 and SLC19A1 Supplementary Figure 18. Survival of ccRCC patients in TRACERx Renal and TCGA when combining aneuploidy and expression of SLC19A1 and ENPP1 Supplementary Figure 19. Association between overexpression of SLC19A1 and survival across multiple tumour types Supplementary Figure 20. Validation of TME composition inference from bulk RNA-sequencing using immunohistochemistry Supplementary Figure 21. TME ITH is pervasive and variable in the TRACERx Renal cohort but does not correlate with clinical outcomes Supplementary Figure 22. Changes in signatures associated with TME co-occurring with the acquisition of specific drivers and/or tumour evolution Supplementary Figure 23. Evaluation of changes in populations of the TME with occurrence of different driver alterations. Supplementary Figure 24. Similarity in TCR or BCR repertoire across different regions of the primary tumour does not associate with survival Supplementary Figure 25. BCR clones can be shared across distinct samples in TRACERx Renal Supplementary Figure 26. Shared TCR clones have higher clonality in tumour samples in TRACERx Renal Supplementary Figure 27. Changes in the BCR repertoire do not associate with clonal evolution in TRACERx Renal Supplementary Figure 28. Association between simimlarity of the TCR/BCR repertoire and genetic intra-tumour heterogeneity Supplementary Figure 29. UMAP representing intra and interpatient variation in HERV expression in the TRACERx Renal cohort Supplementary Figure 30. Association between HERV expression and corresponding copy-number Supplementary Figure 31. Representation of genomic coordinates for LTR elements overexpressed in VHL altered tumour samples Supplementary Figure 32. Association between HERV expression and abundance of different TME populations Supplementary Figure 33. Representation of genomic coordinates for LTR elements significantly associated with survival
