# Supplementary material for: Tracking Nongenetic Evolution from Primary to Metastatic ccRCC: TRACERx Renal
Source: Cancer Discov. 2025 Jan 9;15(3):530–52. doi: 10.1158/2159-8290.CD-24-0499 (PMC11873726; doi:10.1158/2159-8290.CD-24-0499)
Supplement: Supplementary Note 1 — Inputting information from previous TRACERx Renal studies [file cd-24-0499_supplementary_note_1_suppsd1.docx]

# Supplementary Note 1

Inputting information from previous TRACERx Renal studies

The TRACERx Renal consortium previously described the genomic evolution of 101 ccRCC patients where 1,206 primary tumor samples were sequenced with bespoke targeted sequencing panel (612x median coverage, range 105-1,520x) containing probes covering over 110 ccRCC driver genes (driver panels *Panel_v3*, *Panel_v5* and *Panel_v6* were used in this study and included 110, 119 and 130 driver genes, respectively, which were selected in the source publication by its recurrent mutations in TCGA and other studies). 107 and 81 of these primary tumor samples were additionally profiled by whole exome sequencing (WES) and whole genome sequencing (WGS) from 17 and 27 different patients, respectively. 38 of the 101 ccRCC patients in TRACERx Renal had at least one matched metastatic tumor sample successfully subjected to DNA targeted sequencing.

In this study, we leverage bulk RNA-sequencing of a subset of 243 samples from 79 patients to interrogate non-genetic ccRCC evolution. RNA subjected to RNA-Sequencing in this study was extracted from the same sample where we previously obtained the DNA sequenced in the previous publications [(1)](https://paperpile.com/c/vAUSFq/wy8U). Therefore, we could input previously described genetic and phylogenetic information from each patient and tumor sample in this study to complement RNA-sequencing data [(1,2)](https://paperpile.com/c/vAUSFq/wy8U+uBZm). Specifically, from the prior TRACERx Renal studies, we collect the following information: i) presence / absence of SCNAs and point mutations in driver genes (and promoter methylation determined by methylation-specific PCR in the case of *VHL*) [(1)](https://paperpile.com/c/vAUSFq/wy8U), ii) genome-wide copy-number profiles (including a previously estimated measure of aneuploidy burden, *weighted genome instability index, wGII*) [(1)](https://paperpile.com/c/vAUSFq/wy8U), iii) driver tree reconstruction for each tumor (including a previously estimated overall measure of genetic intratumor heterogeneity, *ITH*) [(1)](https://paperpile.com/c/vAUSFq/wy8U) and iv) tumor assignment to one of 7 different evolutionary subtypes [(1)](https://paperpile.com/c/vAUSFq/wy8U). We include below a concise description of the previously used methodology to derive this information for TRACERx Renal, which is more extensively detailed in the corresponding source publication(s) [(1)](https://paperpile.com/c/vAUSFq/wy8U).

### Identification of driver alterations and copy-number profiles

Driver mutations were identified using a comprehensive multi-step process involving both single nucleotide variants (SNVs) and small insertion/deletions (INDELs) derived from multi-region DNA sequencing (driver panel and whole exome sequencing). Paired-end reads (2x100bp) generated by HiSeq or NextSeq were aligned to the human genome reference (hg19) using BWA v0.7.15. Post-alignment processing included the use of Samtools v1.3.1 for intermediate file handling and Picard 1.81 for deduplication. SNV calling was conducted with Mutect v1.1.7, while INDELs were identified using VarScan v2.4.1 and validated with Scalpel v0.5.3. Specific settings for these tools are detailed in the source publication [(1)](https://paperpile.com/c/vAUSFq/wy8U).

The filtering criteria for SNVs included minimum variant allele frequencies, genomic location, strand read support, sequencing depth, and cancer cell fraction thresholds, filtering variants with a final CCF < 0.1 (see source publication for entire filtering criteria [(1)](https://paperpile.com/c/vAUSFq/wy8U)). Suspected artifact variants, based on inconsistent allelic frequencies, were manually reviewed and excluded if necessary. Variants were annotated using Annovar, and a mutation was considered deleterious if two out of i) SIFT, ii) PolyPhen2, and iii) MutationTaster predicted the mutation as deleterious. Mutations detected in high-confidence driver genes (*VHL, PBRM1, SETD2, PIK3CA, MTOR, PTEN, KDM5C, CSMD3, BAP1, TP53, TSC1, TSC2*) were defined as driver mutations.

SCNA (somatic copy number alteration) calling was performed using CNVkit v0.7.3, followed by ABSOLUTE v1.0.6 for purity, ploidy, and absolute copy number estimations. Copy number profiles were reviewed by three researchers to ensure accuracy, with driver SCNAs identified by overlapping with known driver regions, with gains and losses being called relative to overall sample wide estimated ploidy. The accuracy of the copy-number profiles obtained by targeted panel sequencing was demonstrated by a concordance of 87% between panel-derived and WGS-derived copy-number profiles in the subset of patients with both data modalities available. As part of the source publication, the proportion of the genome - across the 22 autosomes - with aberrant copy number was estimated as the weighted genome instability index (wGII), which we used as an estimate of aneuploidy burden in this study.

For the purpose of this study, we use driver mutations and SCNA calls as obtained in this source publication to i) identify a potential impact of copy-number alterations and the acquisition of epigenetic driver alterations to transcriptional intratumor heterogeneity (see section *“Correlates of transcriptional and TME I-TED”* in *Methods*), ii) determine gene expression changes associated with acquisition of 9p and 14q loss (see section *“Association of subclonal driver alterations and changes in gene expression”* in *Methods*), iii) interrogate the expression of the cGAS-STING pathway in tumor samples with varying aneuploidy burden (see section “*Differential expression analysis of cGAS-STING genes by aneuploidy”* in *Methods*), iv) identify how changes in the TME track with the acquisition of new driver alterations (see section *Per-patient changes in the TME* in Methods) and v) determine how the acquisition of main ccRCC drivers *VHL, PBRM1, SETD2* and *BAP1* influences overall HERV expression (see section *“Patterns of variation of HERV expression, link to TME and adaptive immune response”* in *Methods*).

### Driver tree reconstruction

##### Subclonal deconstruction of mutations

To estimate the clonality of mutations within a tumor region, the following formula was applied in prior TRACERx Renal studies [(1)](https://paperpile.com/c/vAUSFq/wy8U):

[
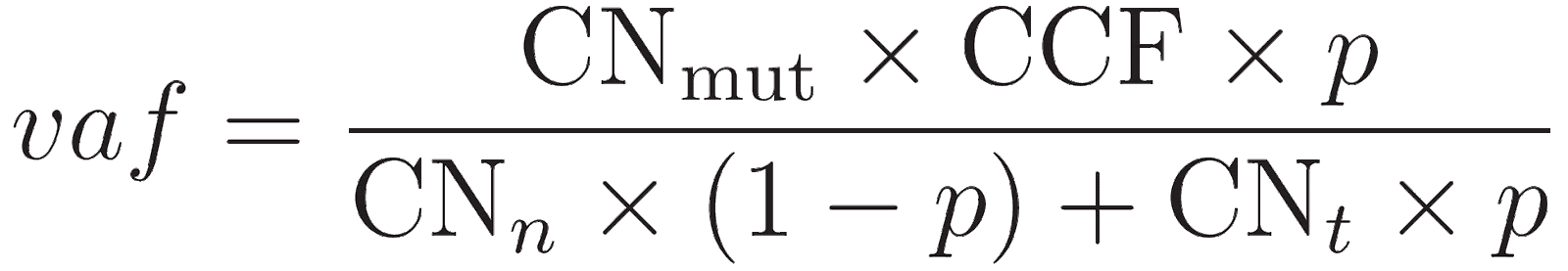
](http://www.sciweavers.org/tex2img.php?bc=Transparent&fc=Black&im=jpg&fs=100&ff=modern&edit=0&eq=vaf%3D%5Cfrac%7B%5Ctext%7BCN%7D_%7B%5Ctext%7Bmut%7D%7D%20%5Ctimes%20%5Ctext%7BCCF%7D%20%5Ctimes%20p%7D%7B%5Ctext%7BCN%7D_%7Bn%7D%20%5Ctimes%20(1%20-%20p)%20%2B%20%5Ctext%7BCN%7D_%7Bt%7D%20%5Ctimes%20p%7D%E2%80%8B#0)

where:

- **vaf** is the variant allele frequency,
- **p** is the estimated tumor purity,
- **CNt** and **CNn** are the tumor and normal locus-specific copy numbers (assumed to be 2 for autosomal chromosomes),
- **CCF** is the cancer cell fraction,
- **CNmut** is the number of chromosomal copies carrying the mutation, ranging from 1 to CNt.

We assigned possible CCF values from 0.01 to 1 and iteratively tested each possible CNmut to identify the best fit for the cancer cell fraction. Since subclonal reconstruction used driver genes and the accuracy of the estimated CCF is limited by the size of the panel, mutations with CCF>0.5 were defined as clonal mutations, mutations with CCF≤0.5 and CCF>0.1 were defined as subclonal and mutations with CCF < 0.1 were excluded. Further, a mutation was classified as clonal in a tumor only if it was determined as clonal in all the regions of the tumor. Long INDELs affecting >6 bp were considered clonal if present in all regions, despite potential VAF underestimation.

##### SCNA Clonality

To determine SCNA clonality, SCNAs present in all tumor regions were classified as clonal, and those present in some but not all regions were classified as subclonal.

##### Driver Tree Reconstruction

We constructed a matrix indicating the presence or absence of nonsynonymous and synonymous mutations, DNVs, INDELs, and arm-level SCNAs for each tumor. Valid clusters for driver events required at least two arm-level SCNAs or one non-synonymous mutation. These clusters were ordered into a clonal hierarchy using TRONCO, resulting in driver trees.

For patients with whole-exome sequencing data, clustering was performed using PyClone Dirichlet process clustering. Pre-clustering CCF estimates were used, setting major allele copy numbers to 2, minor allele copy numbers to 0, and purity to 0.5, which allowed clustering to simply group clonal and subclonal mutations based on their pre-clustering CCF estimates. PyClone ran for 10,000 iterations with a burn-in of 1,000, using default parameters except for var_prior set to 'BB' and ref_prior to 'normal'.

##### Limitations

While our Driver Panel phylogenies are based on fewer clonal markers compared to whole exome/genome data, several measures ensured the robustness of the derived phylogenetic trees in the source publication [(1)](https://paperpile.com/c/vAUSFq/wy8U):

1. Ultra-deep (>500x sequencing) coverage for stable CCF estimates.
2. A bespoke gene panel enriched for driver events.
3. Cross-capture validation with exome sequencing data confirming tree structures in over 10 cases.
4. Panel sequencing allowed extensive tumor sampling, with over 1,200 biopsies sequenced, enhancing the robustness of spatial sampling.

For the purpose of this study, we leveraged driver trees to i) correlate transcriptional distance, TME distance and BCR/TCR similarity to *clonal distance* in the driver tree (see section *“Association of clonal distance with matched transcriptional or microenvironmental distance”* in *Methods*), ii) determine primary tumor regions containing metastasis seeding and non-seeding (sub)clones (see section *“Comparison of transcriptional and microenvironmental distances between metastases and matched seeding and non-seeding primary regions”* in *Methods*), iii) identify transcriptional and TME changes tracking with progressive evolution from earlier to later clones within different patients (see sections *“Gene expression and TME assignment to individual tumor clones”* and *“Association of pathway expression with distance to the MRCA”* in *Methods*)

### Assignment to evolutionary trajectories

We previously described seven different evolutionary trajectories characterized by different patterns of driver acquisition, timing, mutual exclusivity and co-occurrence, which also displayed distinct levels of chromosomal complexity and varied clinical outcomes [(1)](https://paperpile.com/c/vAUSFq/wy8U).

The basis of the classification into evolutionary subtypes are the patterns of driver co-occurrence, ordering and mutual exclusivity we previously described [(1)](https://paperpile.com/c/vAUSFq/wy8U). In that publication, we leveraged this information to create a rule-based classification to assign different tumors to different evolutionary subtypes on the basis of the detected clonal and subclonal drivers. More specifically, we applied the following rules in hierarchical order: i) presence of more than or equal to 2 clonal alterations in *BAP1*, *PBRM1*, *SETD2* or *PTEN* meant assignment to ‘*‘multiple clonal driver’*’ group, ii) presence of only clonal/subclonal *BAP1* alterations along with *VHL* mutations - and no other ‘‘core’’ driver mutations meant assignment to the ‘*‘BAP1 driven’*’ group, iii) presence of clonal/subclonal alterations in *PBRM1* mutation followed by a *SETD2* mutation in the phylogenetic reconstruction meant assignment to the ‘*‘PBRM1-SETD2’*’ group, iv) presence of clonal/subclonal alterations in *PBRM1* followed by a PI3K pathway mutation in the phylogenetic tree meant assignment to the ‘*‘PBRM1-mTOR*’’ group, v) presence of clonal/subclonal alterations in *PBRM1* followed by driver SCNA event(s) meant assignment to the ‘*‘PBRM1-SCNA*’’ group, vi) absence of *VHL* mutation or methylation meant assignment to ‘*‘VHL wildtype*’’ group, vii) presence of *VHL* as the only ‘‘core’’ driver mutation meant assignment to the ‘‘*VHL monodriver’*’ group. The stability and validity of this rule based classification was demonstrated in the original publication using unsupervised clustering [(1)](https://paperpile.com/c/vAUSFq/wy8U).

These evolutionary subtypes therefore represent different routes to ccRCC progression. In this study, we aimed to understand how the TME shapes or is shaped by matched genetic co-evolution. To this end, we analyzed the TME composition, inferred by deconvolution of bulk RNA-Seq data (see section “*Comparison of TME composition between different evolutionary trajectories”* in *Methods*), of tumor samples assigned into each of the 7 different trajectories as described above.

## References

1. [Turajlic S, Xu H, Litchfield K, Rowan A, Horswell S, Chambers T, et al. Deterministic Evolutionary Trajectories Influence Primary Tumor Growth: TRACERx Renal. Cell. 2018;173:595–610.e11.](http://paperpile.com/b/vAUSFq/wy8U)

2. [Zhao Y, Fu X, Lopez JI, Rowan A, Au L, Fendler A, et al. Selection of metastasis competent subclones in the tumour interior. Nat Ecol Evol. 2021;5:1033–45.](http://paperpile.com/b/vAUSFq/uBZm)
