# Supplementary material for: Tracking Nongenetic Evolution from Primary to Metastatic ccRCC: TRACERx Renal
Source: Cancer Discov. 2025 Jan 9;15(3):530–52. doi: 10.1158/2159-8290.CD-24-0499 (PMC11873726; doi:10.1158/2159-8290.CD-24-0499)
Supplement: Supplementary Note 2 — Evaluation of the potential to reconstruct TCR repertiore from bulk RNA-Sequencing data [file cd-24-0499_supplementary_note_2_suppsd2.docx]

Supplementary Note 2

Evaluation of the potential to reconstruct the TCR repertoire from bulk RNA-Sequencing data

Targeted TCR sequencing (TCR-Seq) is currently the gold standard approach for evaluation of the TCR repertoire through means of diversity and clonality. However, TCR-Seq is a much less frequently performed sequencing protocol compared with RNA-Seq; thus, a significant amount of RNA-Seq data exists that are under-utilized and can be used to extract TCR data.

In this study, we used RNA-Seq data to profile the TCR repertoire among the cohort. We also performed an in-house evaluation of RNA-Seq-based TCR profiling methods using RNA-Seq data from our ADAPTeR cohort against sample-matched gold standard TCR-Seq as ground truth [(1)](https://paperpile.com/c/mnYCGF/YmGp). Additionally, we would also like to point the reader to a previous benchmarking study published by Peng et al. [(2)](https://paperpile.com/c/mnYCGF/pVBa) which evaluated RNA-Seq-based methods to profile TCR repertoires.

We assembled, to the best of our knowledge, the largest dataset for assessing the capabilities of RNA-Seq-based repertoire profiling methods in the context of ccRCC. The dataset concerns the ADAPTER study, which composed a total of 58 ccRCC tissue samples with matched RNA-Seq and TCR-Seq (TCRβ chain only) data. Due to the number of RNA-Seq-based repertoire profiling methods available, we narrowed them down to 2 of the best performers overall in the Peng et al. study [(2)](https://paperpile.com/c/mnYCGF/pVBa), which are MiXCR and TRUST4.

### **Fewer clonotypes were detected by RNA-Seq-based TCR profiling methods in comparison to gold standard TCR-Seq**

First, we investigated the capturing ability of RNA-Seq-based methods to characterize the TCRβ repertoire. This is based on the sum of confirmed TCRβ clonotype frequencies detected through TCR-Seq (henceforth termed as ground truth) where each distinct sequence of the complementarity-determining regions 3 (CDR3) was considered as one unique clonotype. Expectedly, the total number of unique clonotypes recalled using RNA-Seq-based methods were rather low, accounting for only 3–5% of the total unique clonotypes found using TCR-Seq (Figure S1A). When looking at each sample individually, RNA-Seq-based methods were able to recall up to 36.5% of unique clonotypes in certain samples (Figure S1B). Both MiXCR and TRUST4 have near similar performance, with TRUST4 being able to call more clonotypes (Figure S1A and S1B).

Upon deeper investigation, we see that up to 73% of clonotypes called in RNA-Seq-based methods were confirmed in ground truth (Figure S1C, pie charts).740 clonotypes were only found in RNA-Seq-based methods but not in ground truth. Additionally, both RNA-Seq-based methods individually detected clonotypes which exist in ground truth, but not in the other RNA-Seq-based method (MiXCR, n = 68; TRUST4, n = 563), as well as clonotypes which are uniquely called in that method only (MiXCR, n = 34; TRUST4, n = 541) (Figure S1C, upset plot). The higher number of unique clonotypes recalled in TRUST4 could be attributed to the fact that it is more capable of detecting lower frequency clonotypes compared to MiXCR (Figure S1D).


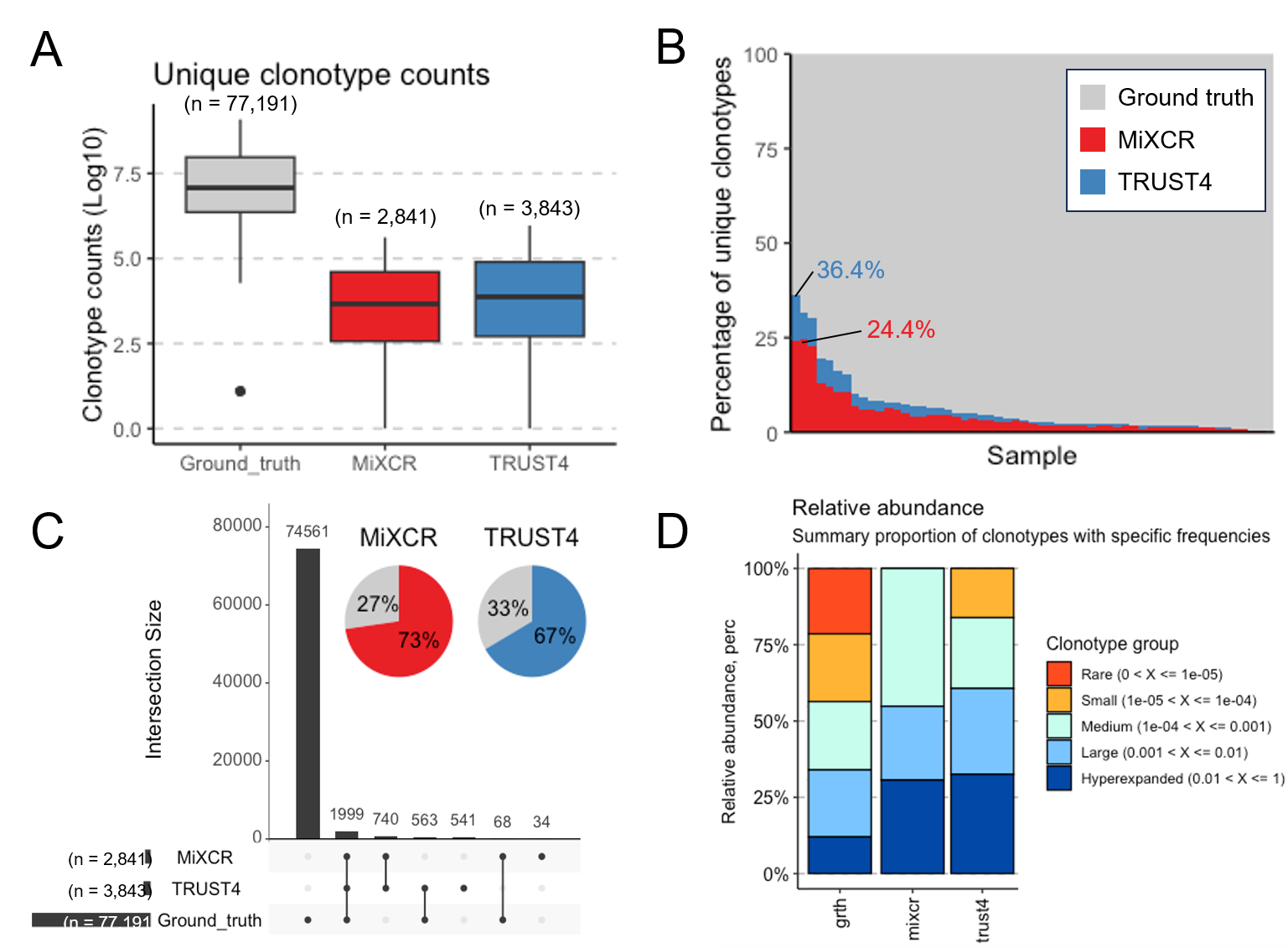


*Figure S1: Comparing RNA-Seq-based methods with ground truth. (A) Box plot shows the number of unique clonotype counts called by each method. (B) Plot shows the percentage of unique clonotypes called by each method in each individual sample. (C) Upset plot shows the number of overlapping clonotypes called by each method. Pie charts show the percentage of clonotypes called by RNA-Seq-based methods which are found in ground truth. (D) Bar chart shows the relative abundance of clonotypes by frequency. gtrh: ground truth bulk TCR-Sequencing in the ADAPTeR study* [*(1)*](https://paperpile.com/c/mnYCGF/YmGp)

When we looked at similarity metrics, both MiXCR and TRUST4 have similar performance when compared against each other but MiXCR had a slight edge when compared against ground truth (Figure 2).


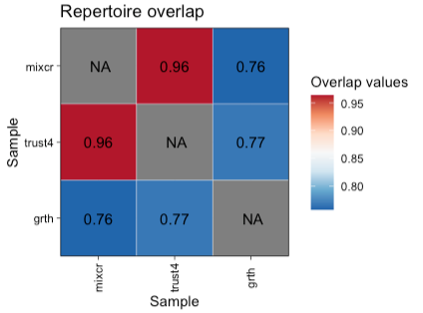


*Figure S2: Heatmap showing the TCR repertoire similarity between methods measured using cosine similarity, a measure of similarity between 2 vectors. The closer the values are to 1, the more similar the vectors are.*

### ***Repertoire diversity from RNA-Seq-based methods correlates well with ground truth data***


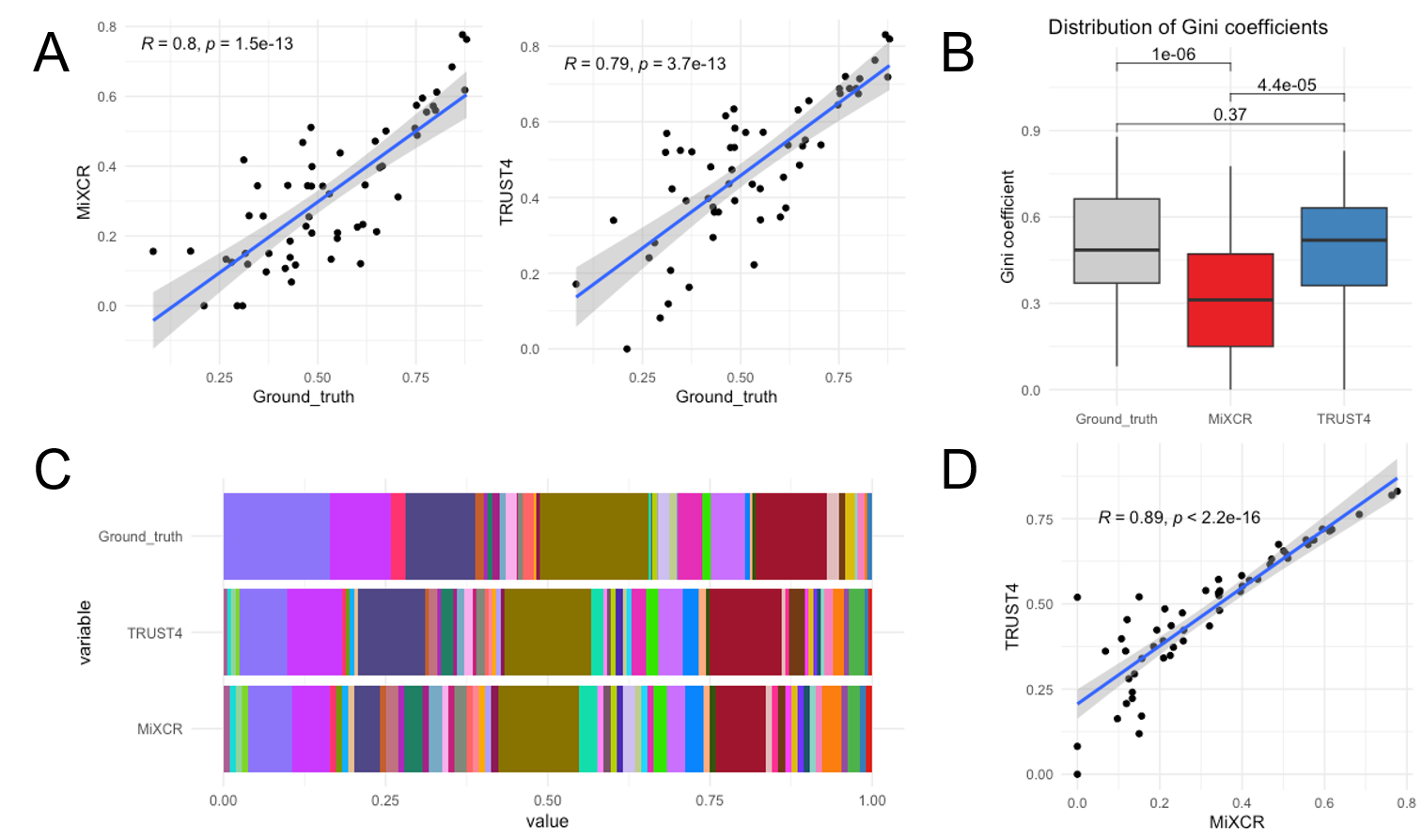


*Figure S3: Evaluation of TCR diversity. (A) Correlation between RNA-Seq-based method and ground truth by Pearson correlation coefficient. (B) Distribution of Gini coefficients for each method. Statistical significance was assessed using Wilicoxon rank sum test and corrected for multiple testing by Bonferroni correction. (C) Distribution of individual clonotypes called by each method. Each color denotes a unique clonotype. (D) Correlation between both RNA-Seq-based methods by Pearson correlation coefficient.*

We next evaluated the ability of RNA-Seq-based methods to estimate the diversity of TCR repertoire using the Gini coefficient. Diversity metrics are well correlated with the one in ground truth (Figure S3A). Notably however, MiXCR overestimated the repertoire diversity (Figure S3B); which when investigated further, we noticed that MiXCR relatively calls a smaller proportion of large, expanded clonotypes while the composition called by TRUST4 is roughly similar to the one in ground truth (Figure S3C). Nevertheless, the diversity estimates for both RNA-Seq-based methods correlates well with each other (Figure S3D).

We next aimed to ascertain the minimum clonotype frequency needed in order to capture the entire repertoire using RNA-Seq-based methods. Both MiXCR and TRUST4 required clonotypes to have at least a minimum frequency of 0.4% in the pool in order for the whole repertoire to be detected (Figure S4).

Based on this quality control, we consider that the accuracy of TCR clonality and TCR repertoire similarity between samples sufficiently approximates the results obtained for both metrics with bespoken bulk TCR sequencing to detect significant, large-effect size, differences in the TRACERx Renal study.


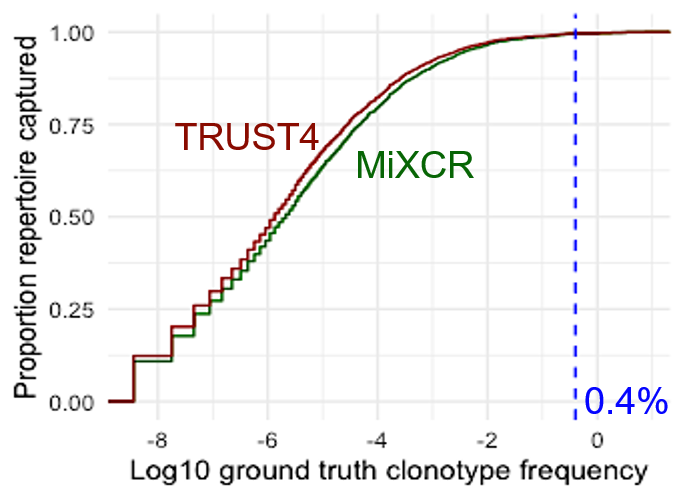


*Figure S4: Curve plot shows the relationship between the frequency of the ground truth clonotype and the proportion of the repertoire captured.*

## References

1. [Au L, Hatipoglu E, Robert de Massy M, Litchfield K, Beattie G, Rowan A, et al. Determinants of anti-PD-1 response and resistance in clear cell renal cell carcinoma. Cancer Cell. 2021;39:1497–518.e11.](http://paperpile.com/b/mnYCGF/YmGp)

2. [Peng K, Nowicki TS, Campbell K, Vahed M, Peng D, Meng Y, et al. Rigorous benchmarking of T-cell receptor repertoire profiling methods for cancer RNA sequencing. Briefings in Bioinformatics. 2023;24](http://paperpile.com/b/mnYCGF/pVBa):bbad220
