# Supplementary material for: Tracking Nongenetic Evolution from Primary to Metastatic ccRCC: TRACERx Renal
Source: Cancer Discov. 2025 Jan 9;15(3):530–52. doi: 10.1158/2159-8290.CD-24-0499 (PMC11873726; doi:10.1158/2159-8290.CD-24-0499)
Supplement: Supplementary Figures 1-33 — All Supplementary Figures are provided in PDF format with the corresponding legend after each figure. Supplementary Figure 1. Genetic and clinical composition of the TRACERx Renal cohort Supplementary Figure 2. Comparison between transcriptional inter and intratumour heterogeneity Supplementary Figure 3. Representation of I-TED to measure transcriptional intratumour heterogeneity and robustness analysis Supplementary Figure 4. Transcriptional ITH is not associated with poorer outcomes in ccRCC Supplementary Figure 5. Subclonal 9p loss is the subclonal somatic copy-number alteration with the greatest association with transcriptional ITH Supplementary Figure 6. Variance in transcriptional intratumour heterogeneity explained by major clinico-genomic covariates Supplementary Figure 7. Association betweeen transcriptional distance between matched tumour-normal pairs of samples and distance from the tumour sample to the most-recent common ancestor (MRCA) Supplementary Figure 8. Schematic representation of the transcriptional and clonal distance calculation in this study. Supplementary Figure 9. Association between transcriptional and clonal distance between primary-metastasis pairs of samples Supplementary Figure 10. Similarity between primary and matched metastases for different transcriptional signatures depending on the detection of a seeding clone in the primary tumour region Supplementary Figure 11. Schematic representation of the assignment of gene expression to clones Supplementary Figure 12. Heatmap with the results of differential expression between 9p or 14q loss samples and matched 9p and 14q wild-type samples Supplementary Figure 13. Association between loss of 9p and expression of IFN I cluster genes. Supplementary Figure 14. Evaluation of changes in gene expression between 9p wild-type and 9p loss clones in published single-cell RNA-sequencing data Supplementary Figure 15. Survival in patients with high or low proliferation scores and 9p loss or 9p wild-type [file cd-24-0499_supplementary_figures_1-33_suppsf1.pdf]

Supplementary Figures

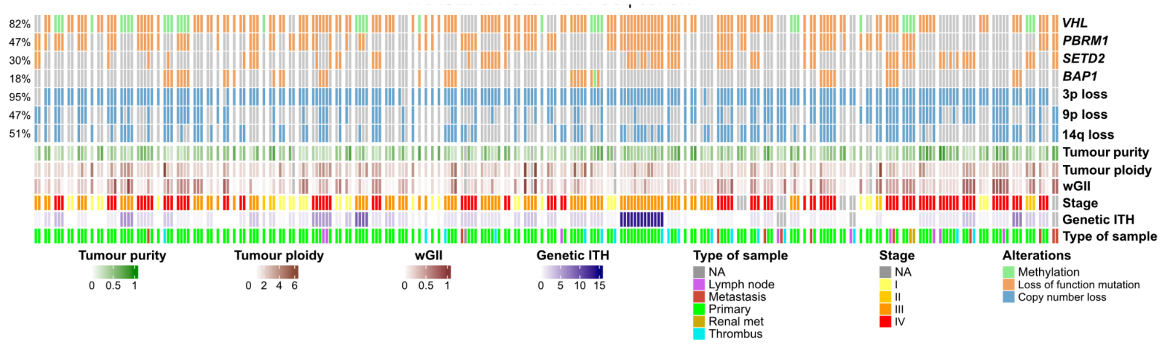

Supplementary Figure 1. TRACERx Renal RNA-Seq cohort. *Overview of genetic and clinical information in 231 tumor samples from 79 TRACERx Renal patients. Columns represent individual tumor samples and are grouped by patient. Light orange indicates loss-of-function mutations (either SNV, DNV or INDEL); light green indicates methylation; blue indicates copy-number loss. Percentages of samples with a driver alteration are highlighted to the left of each row. wGII: weighted genome instability index, a measure of aneuploidy; ITH: intratumor heterogeneity.*

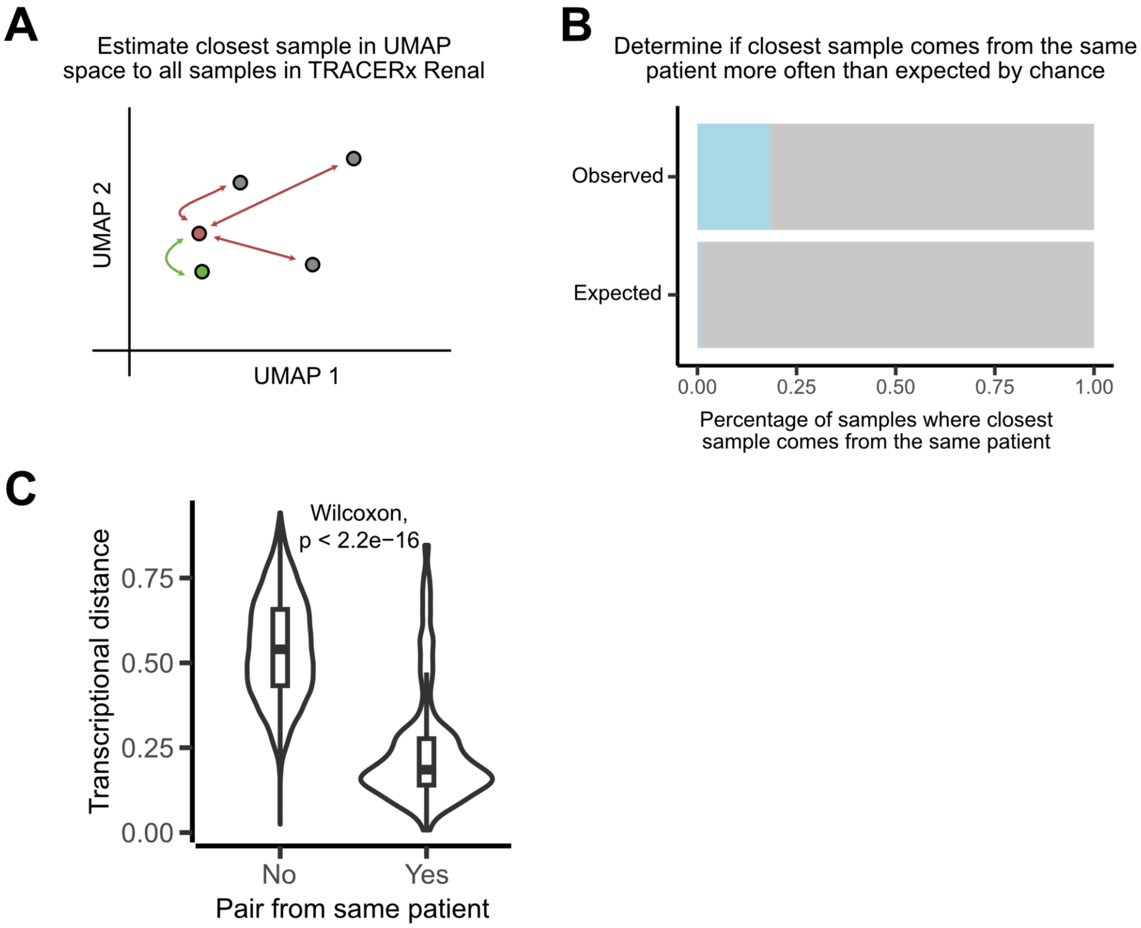

Supplementary Figure 2. Transcriptional inter-patient heterogeneity exceeds intra-patient transcriptional heterogeneity. A) *Schematic representation of the identification of the sample with the most similar transcriptional programme. The closest sample in UMAP space is considered the sample with the most similar transcriptional programme. B) Frequency of samples where the closest sample comes from the same patient (blue) or from another patient (gray) (p-value < 0.001, chi-squared test). Expectation is calculated as the frequency of pairs of samples in the TRACERx Renal cohort that are from the same patient out of all possible pairs. C) Transcriptional distance between pairs of samples from the same patient (representing transcriptional intra-tumor heterogeneity) and different patients (representing transcriptional intertumor heterogeneity) (x-axis), estimated as described in Methods section "Comparison of Transcriptional Intratumor and Intertumor Heterogeneity.".*

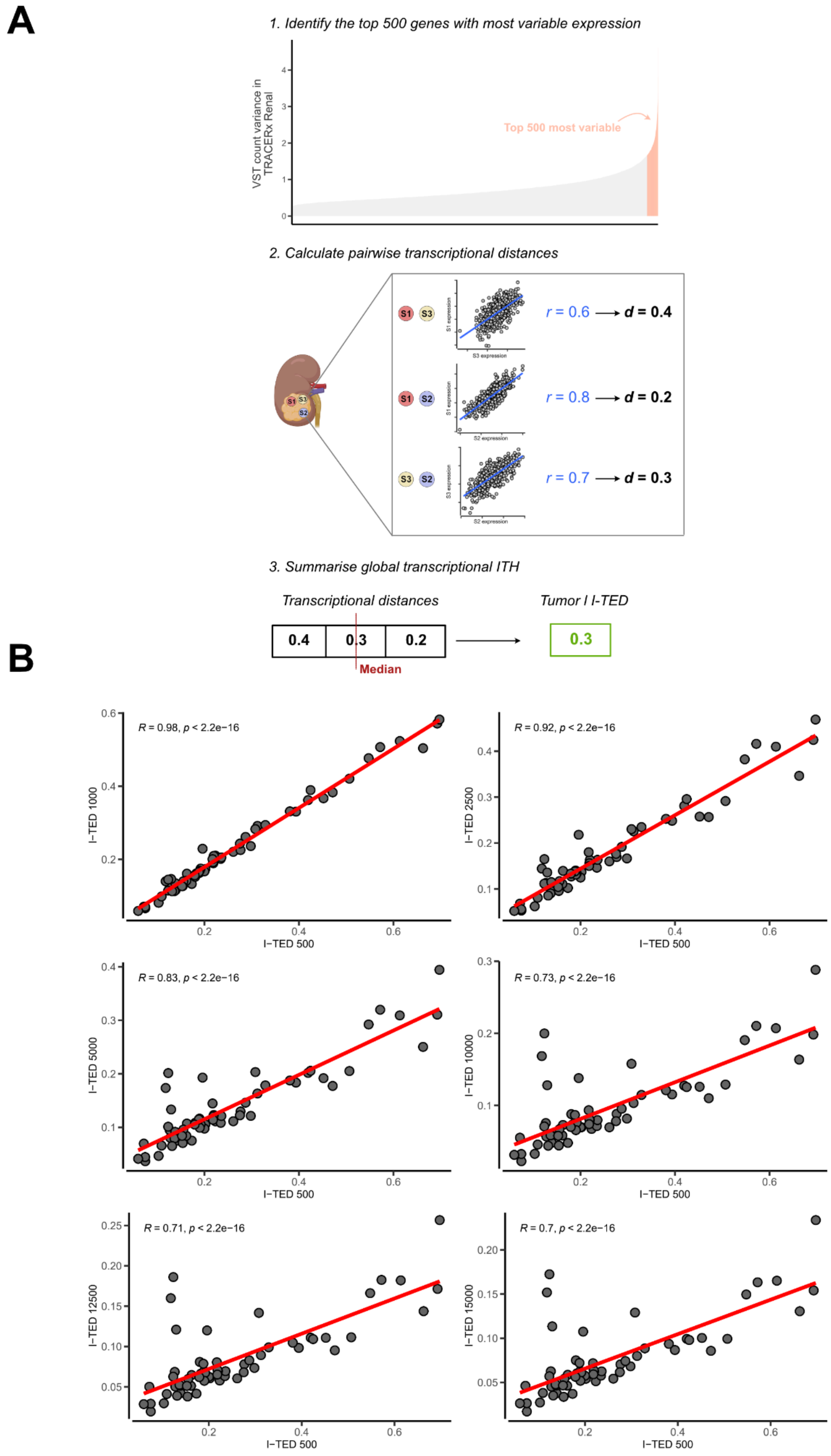

Supplementary Figure 3. I-TED overview. *A) Schematic representation of I-TED calculation within a patient. I-TED, proposed by Martinez-Ruiz et al(6). I-TED summarizes per-patient transcriptional diversity into a single-metric. Estimation of transcriptional distances uses only the expression of the*

500 genes with the highest variance in VST counts, which are identified in the first step (step 1). For all pairs of samples from the same patient in the cohort, transcriptional distance is estimated as 1-r, where r is Pearson's correlation between the VST counts of the top 500 genes in the two samples in the pair (step 2). Finally, I-TED is calculated for each patient as the median of all pairwise transcriptional distances (step 3). B) Robustness of I-TED estimates to the application of increasingly higher subset of genes. I-TED was originally described to be used with the top 500 most variable genes in a cohort (by Martinez-Ruiz et al(6)). We defined scores obtained with this subset of genes as I-TED 500, used throughout this manuscript. These were compared to I-TED estimates obtained when using progressively larger sets of top variable genes in the TRACERx Renal cohort: the top 1,000 (I-TED 1000), top 2,500 (I-TED 2,500), top 5,000 (I-TED 5,000), top 10,000 (I-TED 10,000), top 12,500 (I-TED 12,500) and top 15,000 (I-TED 15,000) most variably expressed genes. Pearson's correlation and p-value of the correlation is indicated at the top of each plot; the red line represents the linear regression between I-TED values obtained with different numbers of genes.

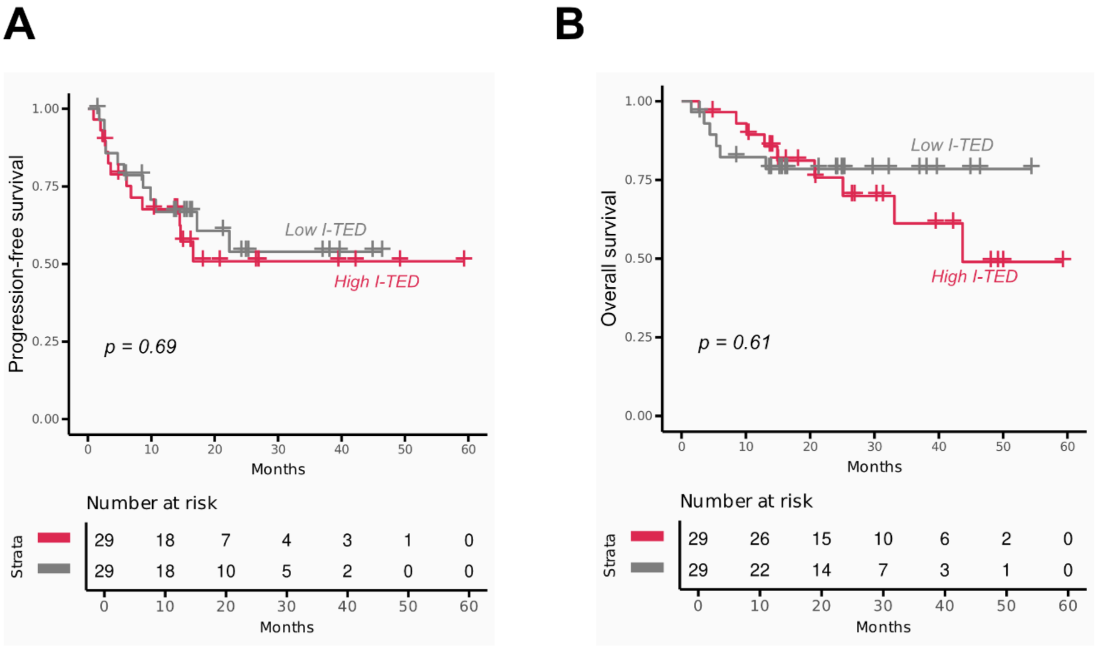

Supplementary Figure 4. Transcriptional ITH is not significantly associated with poorer clinical outcomes in the TRACERx Renal cohort. A) Progression-free survival and B) overall survival *Kaplan-Meier curves stratified by I-TED values above (red) or equal and below median (gray). p-value is obtained by a log-rank test.*

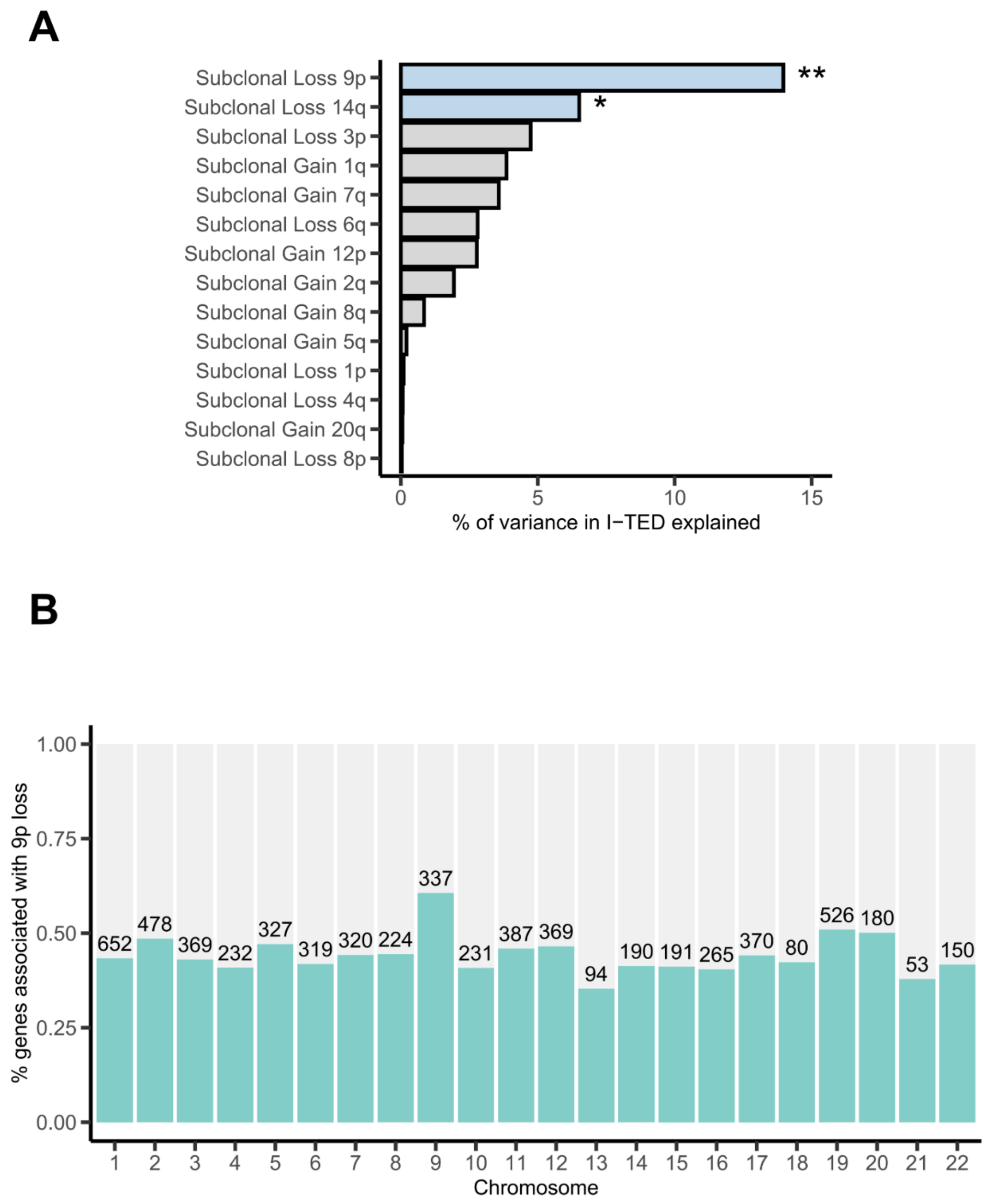

Supplementary Figure 5. Subclonal 9p loss correlates with more extensive genome-wide transcriptional changes than other subclonal SCNAs. A) *Subclonal 9p loss is the driver CNA that explains the highest proportion of primary I-TED score variation across 14 CNA drivers previously identified in TRACERx Renal (2). A multivariable linear regression model of I-TED is fit with subclonality in each of the CNAs indicated in the y-axis as a covariate, together with purity ITH, proportion of genome with subclonal SCNAs, tumor stage, number of regions sampled, tumor size, genetic ITH and presence of subclonal mutation(s) in ccRCC epigenetic drivers (ARID1A, KDM5C, SETD2, BAP1 or PBRM1). p-value of the association between subclonality of a CNA driver and I-TED is indicated with asterisks (**: p-value < 0.01, *: p-value < 0.05). B) Proportion of genes across chromosomes where within-patient expression changes are significantly correlated with concomitant loss of chromosome 9p (green). Total number of genes significantly associated with acquisition of 9p loss per chromosome are indicated on top of each bar.*

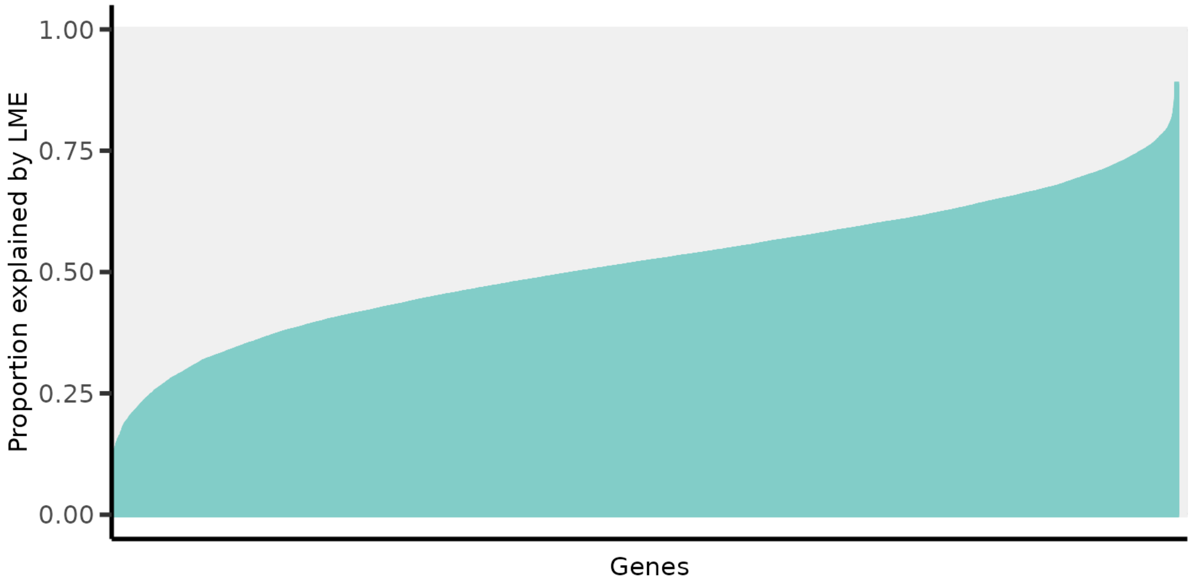

Supplementary Figure 6. Tumor purity, gene dosage, subclonal 9p loss, and mutation of epigenetic drivers underpin less than half the variance of transcriptional variation in TRACERx Renal. *Proportion of the expression differences between primary-primary pairs of samples from the same patient (green), for each of 14,260 genes (x-axis), explained by a linear-mixed effects model including as covariates changes in gene copy-number, differences in tumor purity and differences in the status of 9p loss and mutations in epigenetic driver genes. The linear-mixed effects model corrects for the potential inclusion of multiple pairs of samples from the same patient and whole-genome doubling status.*

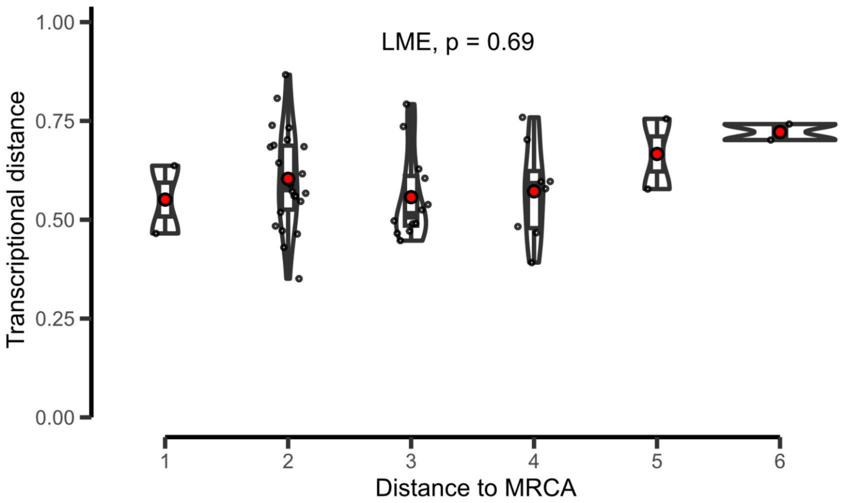

Supplementary Figure 7. Primary tumor samples show magnitude of transcriptional changes independent of distance to MRCA. *Transcriptional distances between 48 primary-normal pairs with increasing distances to the MRCA, a proxy for the clonal distance to the adjacent kidney normal sample. Distance to MRCA is defined as the number of (sub)clonal expansions occurring in between the MRCA of the phylogenetic tree and the clone identified in a given sample. Red points indicate the mean transcriptional distance between pairs of samples in each subgroup. p value is estimated using linear mixed effects model (LME) to correct for the inclusion of multiple pairs of samples from the same patient.*

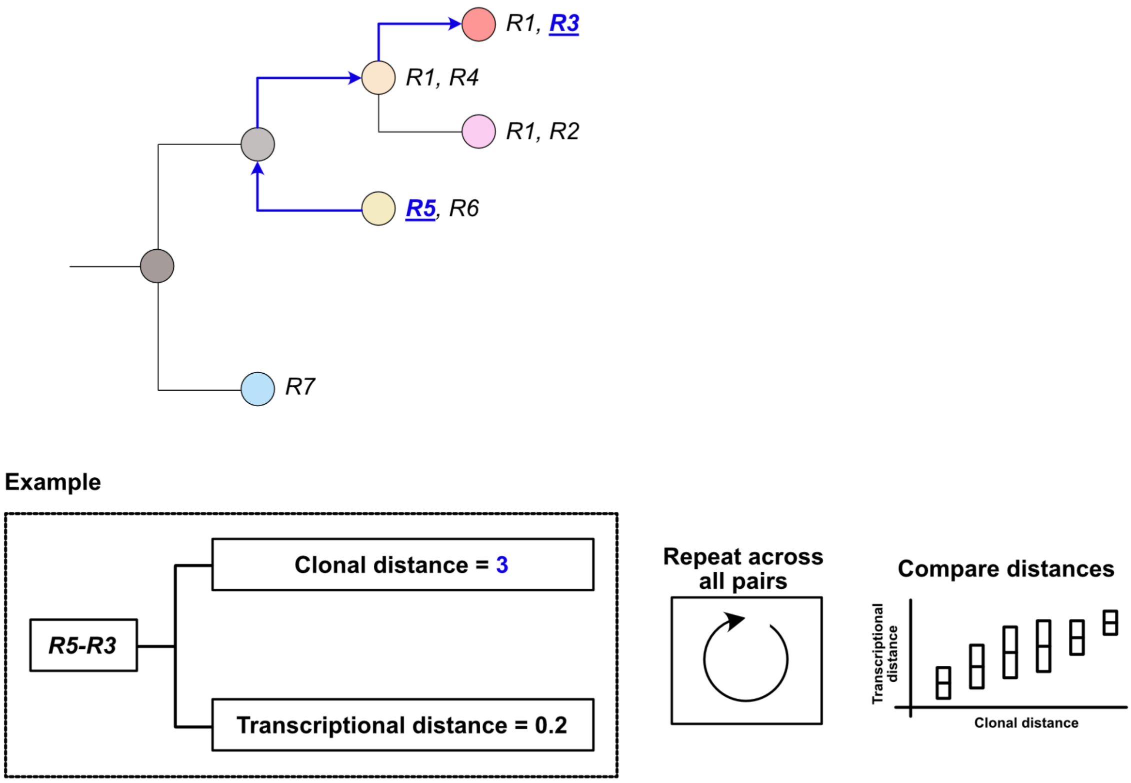

Supplementary Figure 8. Comparing transcriptional distances to clonal distances. *Representative tumor phylogeny and distance calculations between two samples (R5 and R3 in this example). Clonal distance is calculated as the minimum number of edges (highlighted in blue) connecting a clone in a tumor sample (R5) to a clone in the other tumor sample (R3). In essence, this represents the number of (sub)clonal expansions leading to the divergence of the tumor clones identified in two distinct samples. Transcriptional distances are meanwhile calculated from matched bulk RNA-Sequencing data as described in section Quantification of transcriptional and tumor microenvironment (TME) distance in Methods. Repeated calculation of these distances across pairs from the same patients allows to compare transcriptional and clonal distances in the entire cohort.*

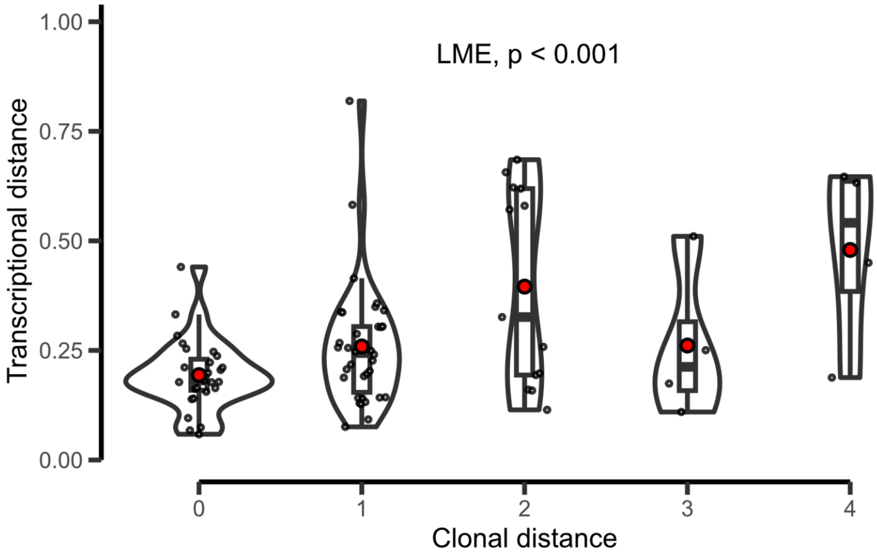

Supplementary Figure 9. Metastases retain gene expression changes acquired during transcriptional evolution. *Transcriptional distances between 82 primary-metastasis pairs with increasing clonal distances. Red points indicate the mean transcriptional distance between pairs of samples with the same clonal distance. p value estimated using linear mixed effects model (LME) to correct for the inclusion of multiple pairs from the same patient and differences in tumor purity.*

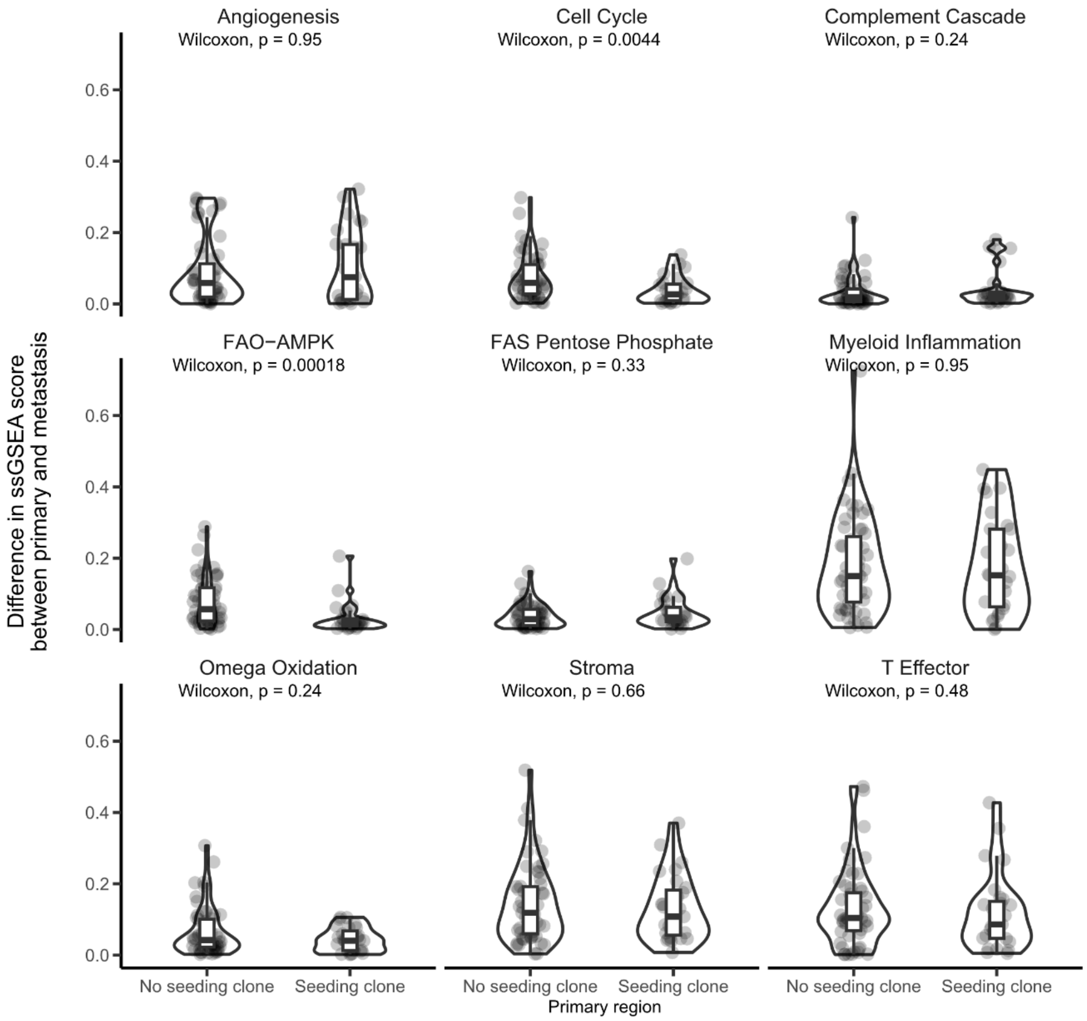

Supplementary Figure 10. Primary regions containing seeding subclones might best represent transcriptional signatures of metastases. *Difference in ssGSEA for the 9 signatures describing molecular subsets of ccRCC in Motzer et al(10) between matched metastasis and primary regions harboring the metastasis seeding subclone (27 seeding primary-metastasis pairs) or not (55 non-seeding primary-metastasis pairs). Weaker resemblance is observed in signatures describing the TME status (angiogenesis, complement cascade, myeloid inflammation, stroma and T effector).*

A)

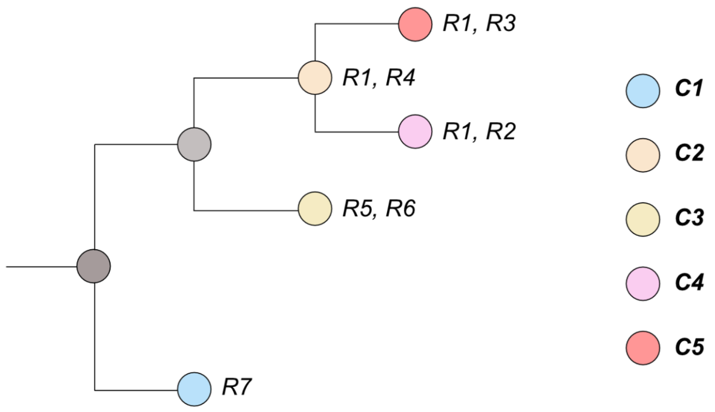

B)

	R1	R2	R3	R4	R5	R6	R7
**Gene X**	2	1	0.8	1	0.7	0.9	1.5
**Gene Y**	1	2	0.5	0.3	0.2	1	2

	= R7	= R4 ~~R1~~	= (R5 + R6) / 2	= R2 ~~R1~~	= R3 ~~R1~~
	**C1**	**C2**	**C3**	**C4**	**C5**
**Gene X**	1.5	1	0.8	1	0.8
**Gene Y**	2	0.3	0.6	2	0.5

Supplementary Figure 11. Clone gene expression assignment. *A) Representative tumor phylogeny. Labels adjacent to each clone indicate samples wherein the clone was detected. Non-sampled clones are colored in grey. B) Transforming gene-sample expression matrix to gene-clone expression matrix. The gene expression values for each clone are the average gene expression values across all monoclonal regions wherein the clone is detected. Polyclonal regions are ignored (R1).*

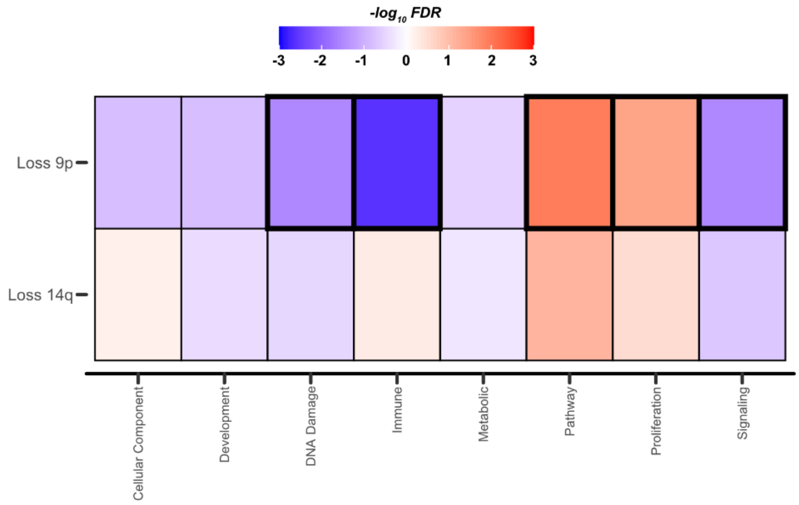

Supplementary Figure 12. Differential expression of hallmark signatures collapsed into functional groups by 9p and 14q status. *Association of transcriptional changes in 50 different hallmark signatures with subclonal 9p and 14q loss in ccRCC, collapsed into 8 different functional groups, as previously described by Martinez-Ruiz et al(6). FDR (False Discovery Rate) was calculated by correcting with the Benjamini-Hochberg method p-values obtained via paired Wilcoxon tests between wildtype and mutant regions (subclonal 9p loss: 20 patients with 44 wild-type and 27 mutant samples; subclonal 14q loss: 26 patients with 45 wild-type and 41 mutant samples). Negative and positive associations are coloured in blue and red, respectively. Highlighted squares indicate significant associations (FDR < 0.05)*

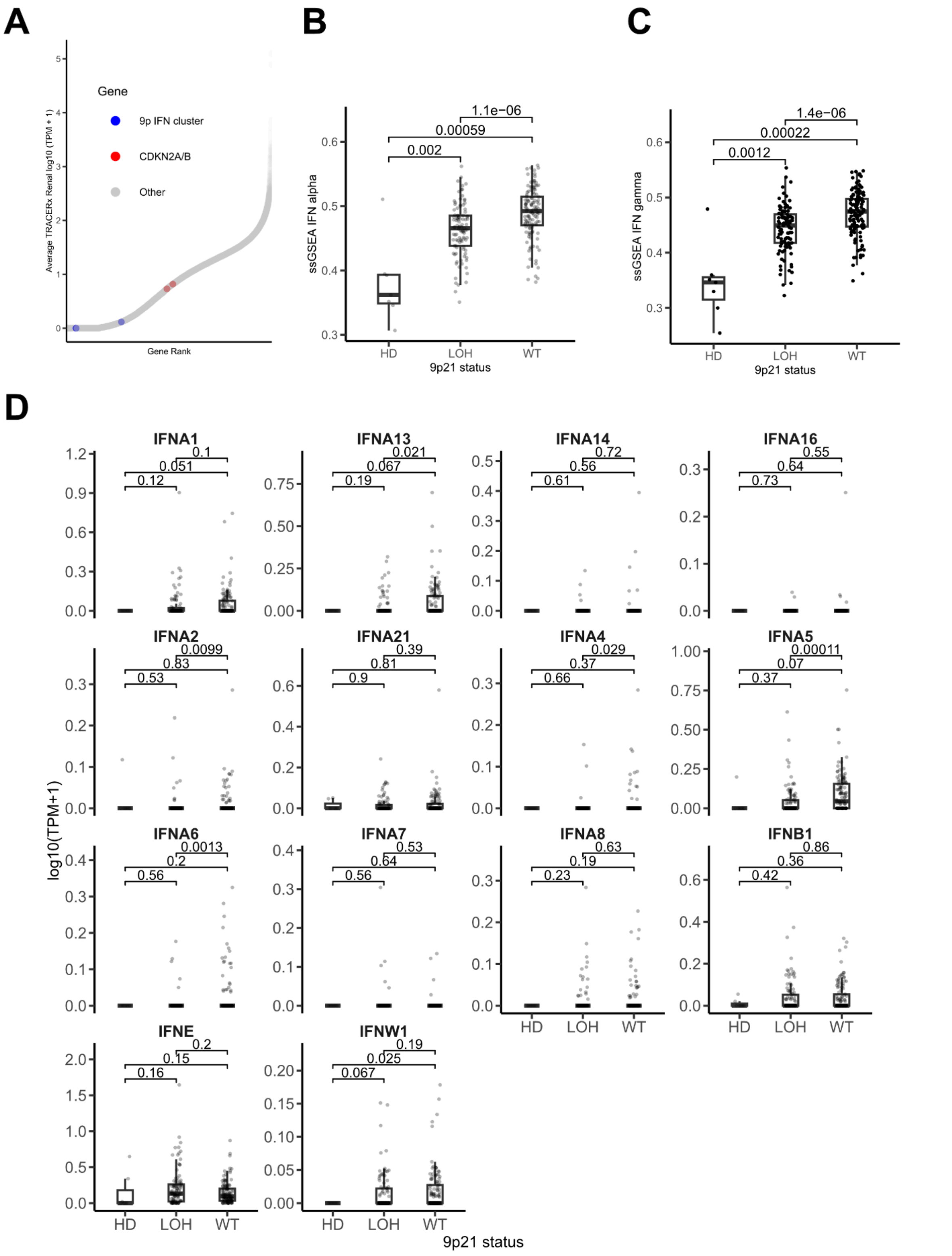

Supplementary Figure 13. 9p loss correlates with lower expression of the IFN I 9p21 gene cluster. *A) IFN I genes (IFNB1, IFNW1, IFNA21, IFNA4, IFNA16, IFNA7, IFNA14, IFNA5, IFNA6, IFNA13, IFNA2, IFNA8, IFNA1, IFNE) show low average expression compared to other genes in ccRCC, both in different chromosome regions (gray) or within the 9p21 locus (CDKN2A or CDKN2B, red), complicating sensitive differential expression analysis. B, C) ssGSEA scores for hallmark 50 MSigDB B) interferon alpha and C) gamma signatures across TRACERx Renal samples with different*

9p21 status: homozygous deletion (HD, 7 samples from 4 patients), heterozygous deletion (LOH: 101 samples from 49 patients) or no loss (WT, 122 samples from 53 patients) at the 9p21 locus. p-values are obtained via Wilcoxon test D) Expression of 9p21 locus IFN I genes (y-axis), quantified by log10 (TPM+1), across 231 TRACERx Renal samples with different copy-number status: homozygous deletions (HD, 7 samples from 4 patients), heterozygous deletion (LOH: 101 samples from 49 patients) or no loss (WT, 122 samples from 53 patients) at the 9p21 locus. p-values obtained via Wilcoxon test

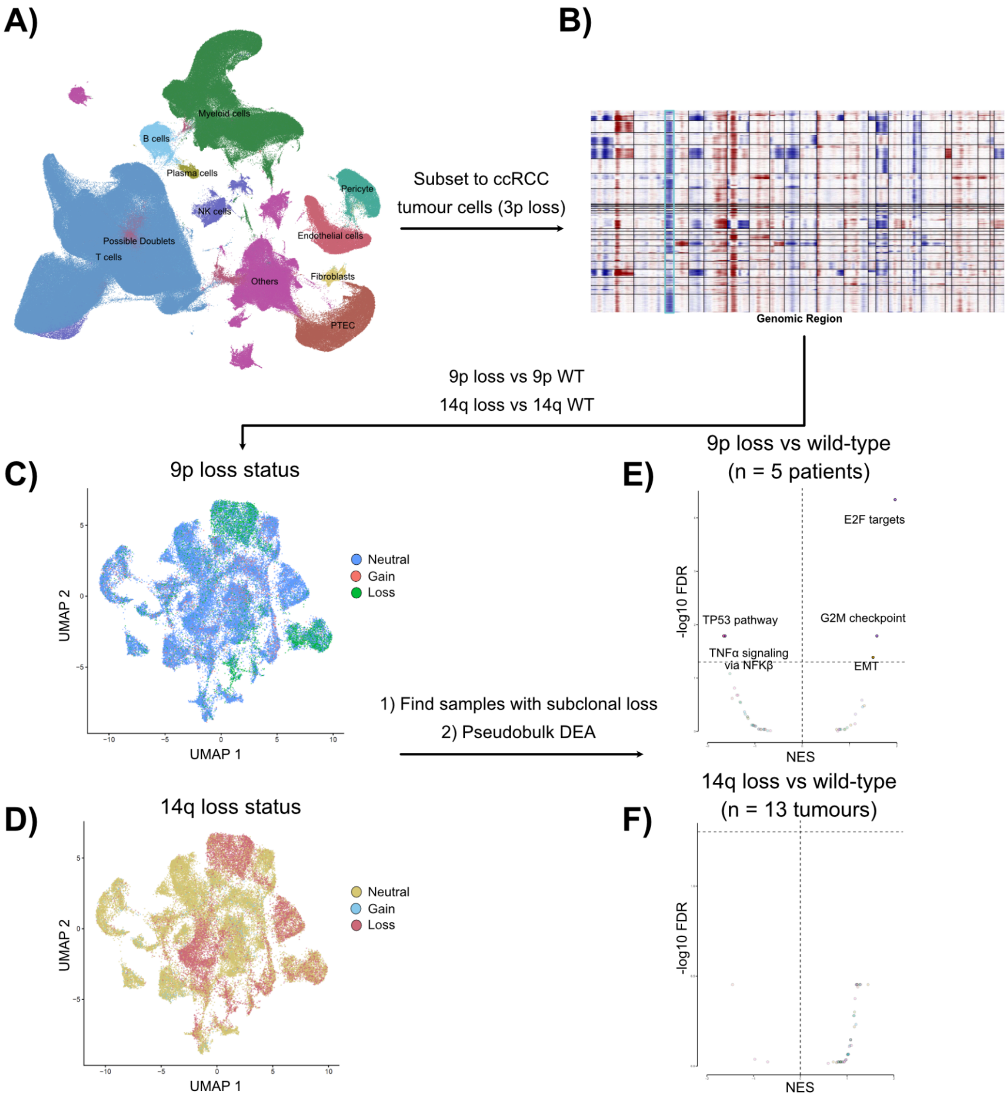

Supplementary Figure 14. 9p loss acquisition is associated with the induction of cell proliferation pathways at single-cell resolution. *A) UMAP representation of 954,023 cells from 8 different studies (1–8). Each point represents a single-cell and colors indicate different cell types. B) InferCNV identifies a population of ccRCC tumor cells based on the pathognomic identification of 3p loss (highlighted in light blue square). Blue color indicates deletion; red color indicates copy number gain. C, D) UMAP representation of 3p loss ccRCC tumor cells colored by C) 9p loss status and D) 14q loss status. E, F) Volcano plot of gene-set enrichment analysis (GSEA) results on the 50 MSigDB hallmark signatures from pseudobulk differential expression analysis of E) 9p loss against wild-type cells or F) 14q loss against wild-type cells. Pseudobulk differential expression analysis was run only on patients with subclonal loss of 9p and 14q, respectively. Subclonal loss was defined as presence of loss in between 10% and 90% of all sequenced cells in a sample, representing at least a total of 10 cells. Horizontal dashed line corresponds to a FDR equal to 0.05. FDR: False discovery rate, NES: Normalized enrichment score.*

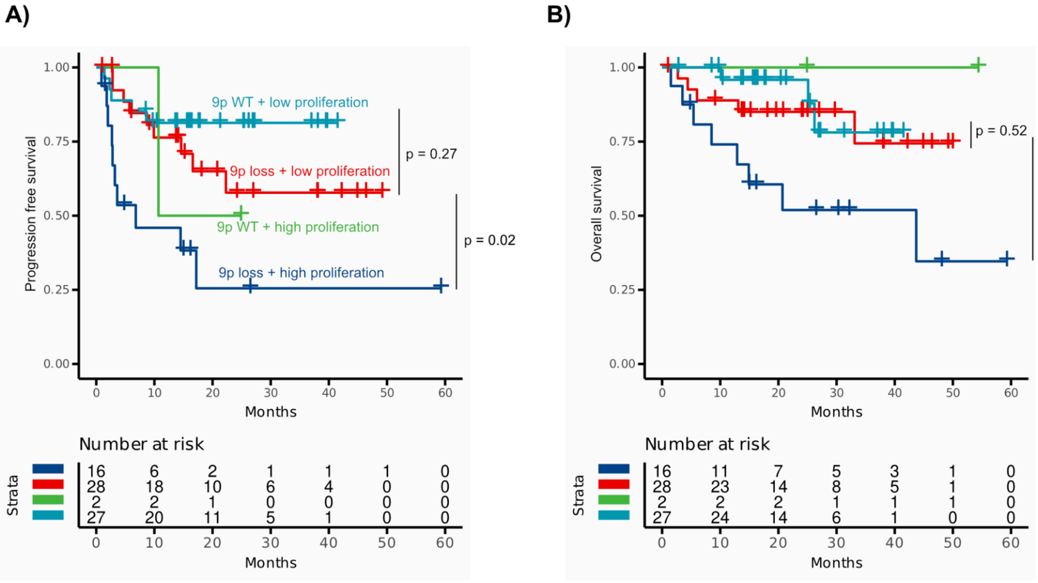

Supplementary Figure 15. Proliferation adds additional prognostic information to 9p loss in the TRACERx Renal cohort. *Kaplan-Meier curves of A) progression-free and B) overall survival stratified by high (top 25%) or low (bottom 75%) expression of proliferation pathways and presence of 9p loss in primary tumor TRACERx Renal samples. For patients with more than one sample in TRACERx Renal, maximum proliferation score was considered. p-value is obtained by a log-rank test.*

A

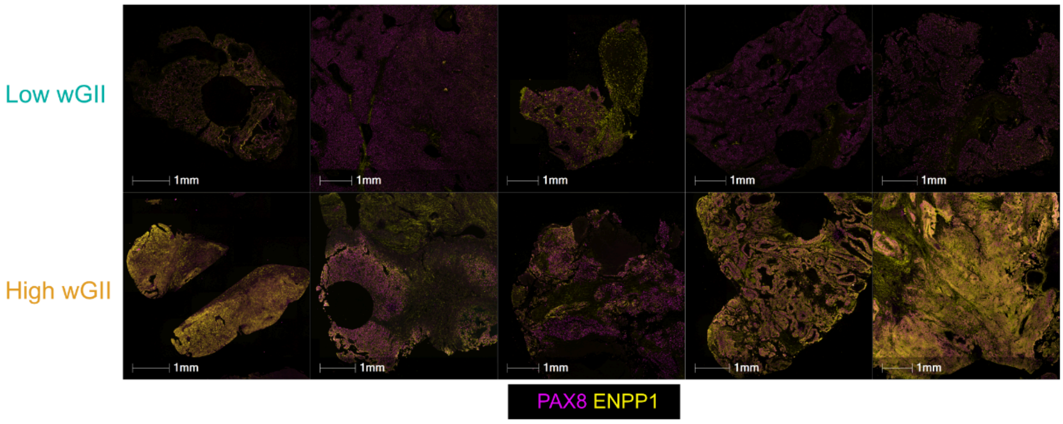

B

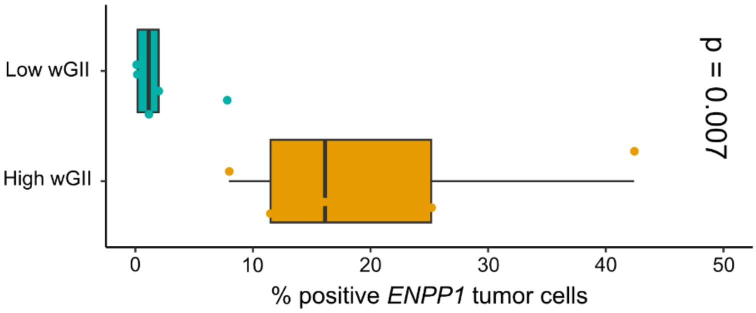

Supplementary Figure 16. Multiplex immunofluorescence quantification of ENPP1 positive cells in ccRCC cells. A) *Multiplex immunofluorescence images of TRACERx Renal tumor samples with low wGII (n = 5, top row) and high wGII (n = 5, bottom row). PAX8, a marker of ccRCC cells, is marked in purple; ENPP1 is in yellow. B) Percentage of ccRCC cells (PAX8 positive) expressing ENPP1 in multiplex immunofluorescence of high wGII (n = 5) and low wGII tumor samples (n = 5).*

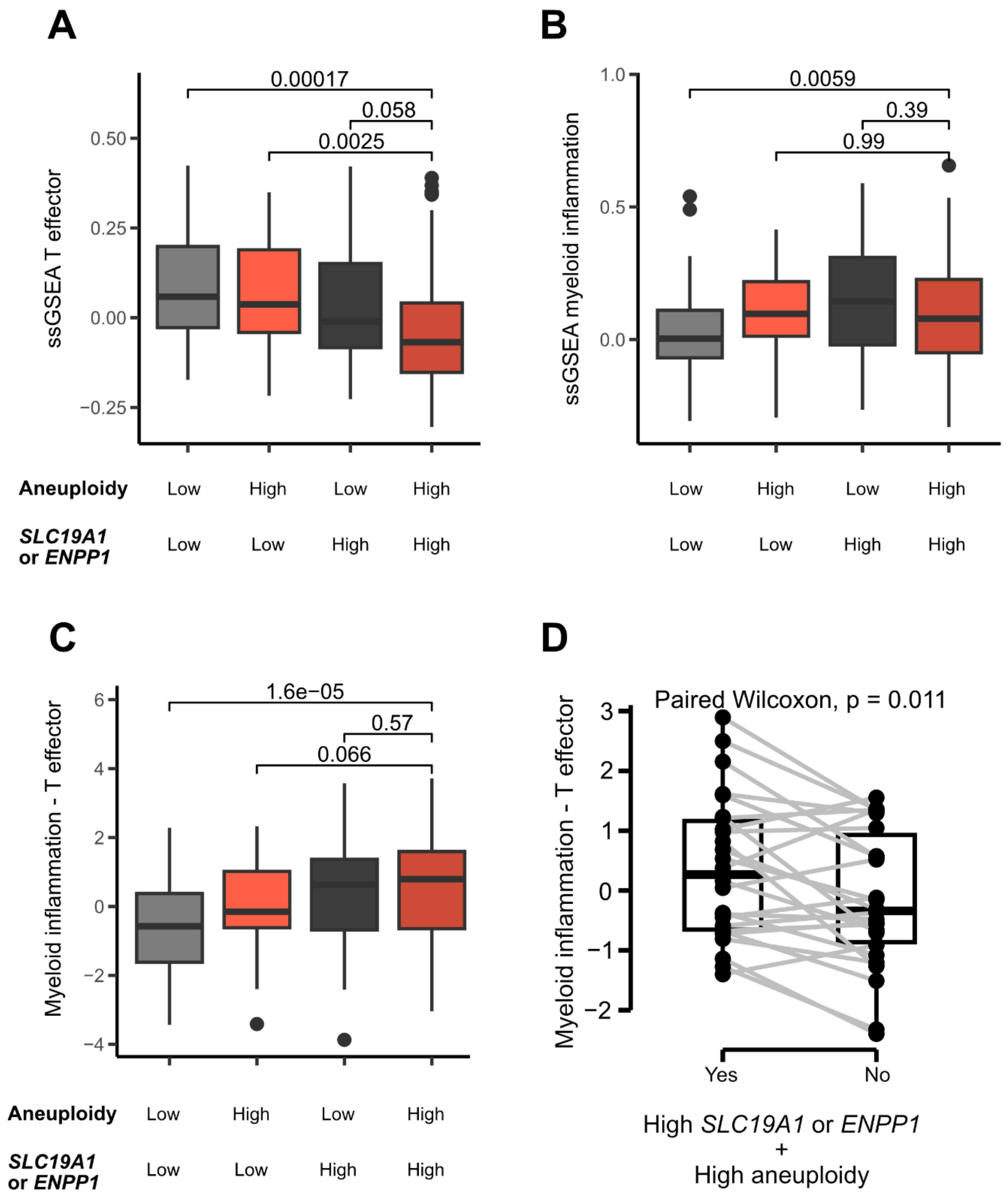

Supplementary Figure 17. Overexpression of SLC19A1 and/or ENPP1 (putative cGAS-STING repressors) correlates with an immunosuppressive TME. *A,B, C) ssGSEA scores for A) T effector and B) myeloid inflammation transcriptional signatures from Motzer et al(10), or C) the difference between Z-scores for T effector and myeloid inflammation signatures. High aneuploidy is defined as wGII scores above or below the median in the TRACERx Renal cohort. SLC19A1 or ENPP1 high is defined as values among the top 25% in the TRACERx Renal cohort for any of both genes. For patients with more than one sample in TRACERx Renal, maximum wGII and SLC19A1 and ENPP1 expression values were considered. High aneuploidy, high SLC19A1 or ENPP1: n = 61 samples; high aneuploidy, low SLC19A1 or ENPP1: n = 52 samples; low aneuploidy, high SLC19A1 or ENPP1: 39 samples; low aneuploidy, low SLC19A1 or ENPP1: 78 samples. Values indicated in the plot are Wilcoxon test p-values. D) Paired analysis between high SLC19A1 or ENPP1 and high aneuploidy (putative cGAS-STING repressed tumor regions) and either low aneuploidy or low SLC19A1 or ENPP1 expression samples within the same patient. Each point represents the average difference*

between Z-scores of ssGSEA scores for T effector and myeloid inflammation signatures in samples categorized in each group. This analysis is only performed in patients with at least one tumor sample categorized into each category (26 total patients)

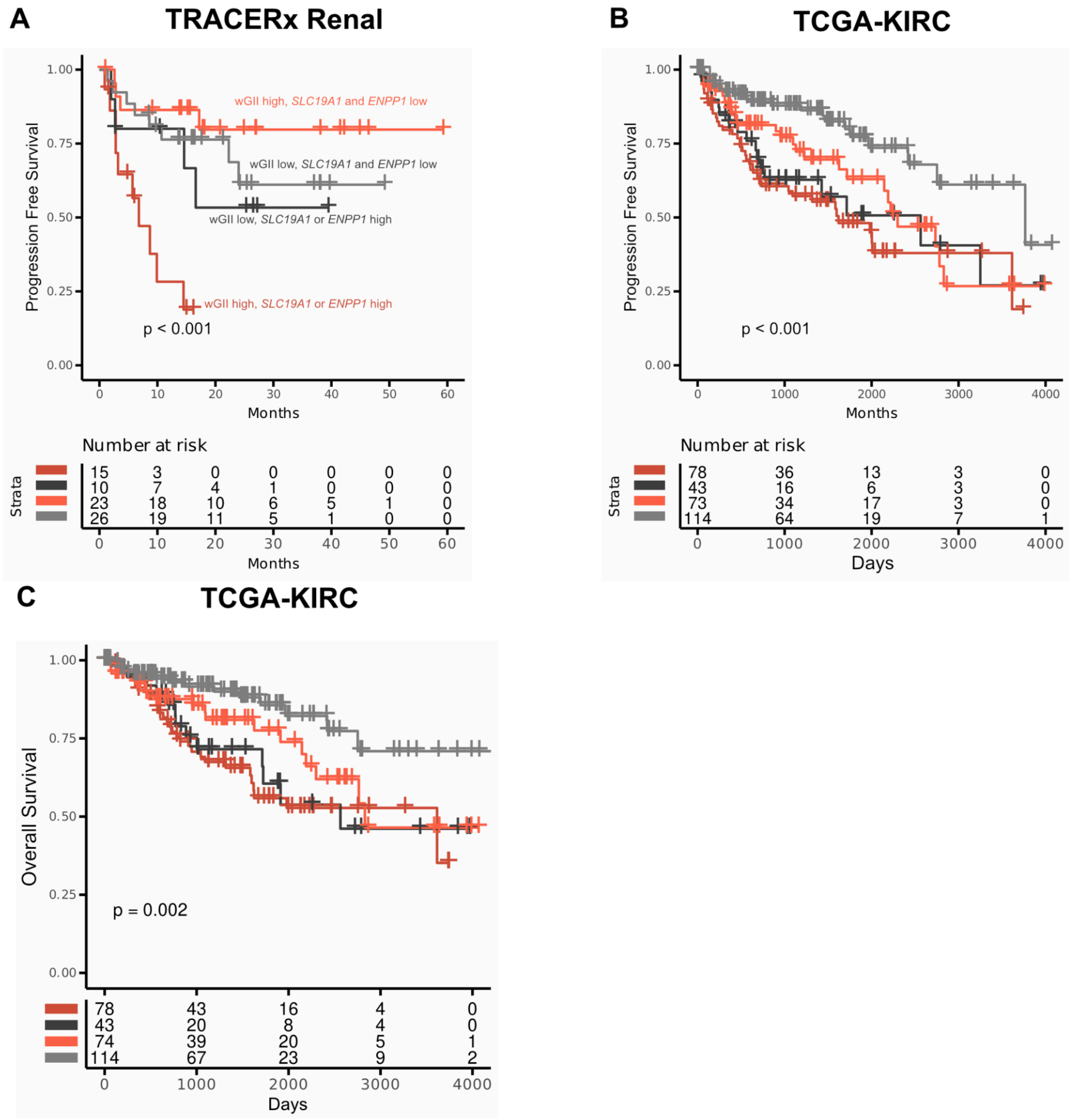

Supplementary Figure 18. Combination of aneuploidy (wGII) and expression of putative cGAS-STING repressors SLC19A1 and ENPP1 stratifies patients into groups with different progression-free survival. *A,B & C) Kaplan-Meier progression-free survival (A,B) and overall survival (C) curves stratified by high (top 25%) or low (bottom 75%) expression of SLC19A1 or ENPP1 and wGII in TRACERx Renal (A) and TCGA-KIRC (B, C). p-value is obtained by a log-rank test. For patients with more than one sample in TRACERx Renal, maximum wGII and SLC19A1 and ENPP1 expression values were considered. Colors in A), B) and C) refer to the same group of patients (red: wGII high and SLC19A1 or ENPP1 high; orange: wGII high and SLC19A1 and ENPP1 low; black: wGII low and SLC19A1 or ENPP1 high; gray: wGII low and SLC19A1 and ENPP1 low).*

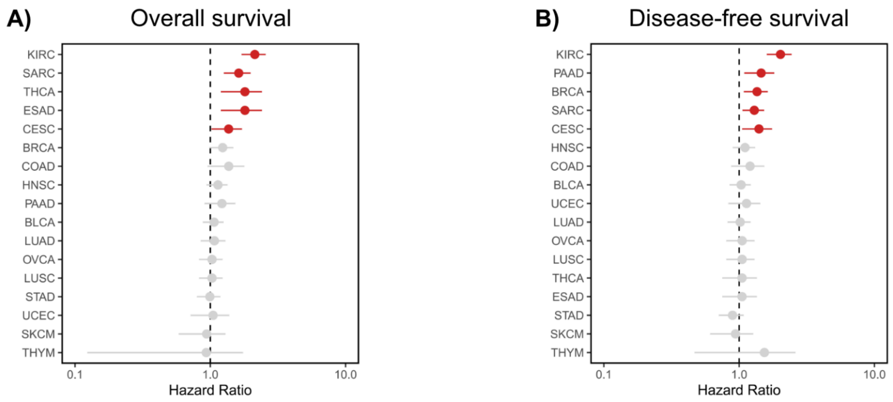

Supplementary Figure 19. SLC19A1 is associated with poorer clinical outcomes across tumor types. *Hazard ratio for A) death and B) disease progression of TCGA patients stratified by SLC19A1 overall expression. Data points represent HR ± 95% CI; red points indicate $p < 0.05$. Tumor types previously analyzed by Li et al, 2021 are indicated in the x-axis (BLCA, bladder urothelial carcinoma; BRCA, breast cancer; CESC, cervical squamous cell carcinoma and endocervical adenocarcinoma; COAD, colon adenocarcinoma; ESAD, esophageal adenocarcinoma; HSCN, Head and Neck squamous cell carcinoma; KIRC, kidney renal clear cell carcinoma; LUAD, Lung adenocarcinoma; LUSC, Lung squamous cell carcinoma; OVCA, ovarian serous cystadenocarcinoma; PAAD, pancreatic adenocarcinoma; SARC, sarcoma; SKCM, skin cutaneous melanoma; STAD, stomach adenocarcinoma; THCA, thyroid carcinoma; THYM, thymoma; UCEC, uterine corpus endometrial carcinoma).*

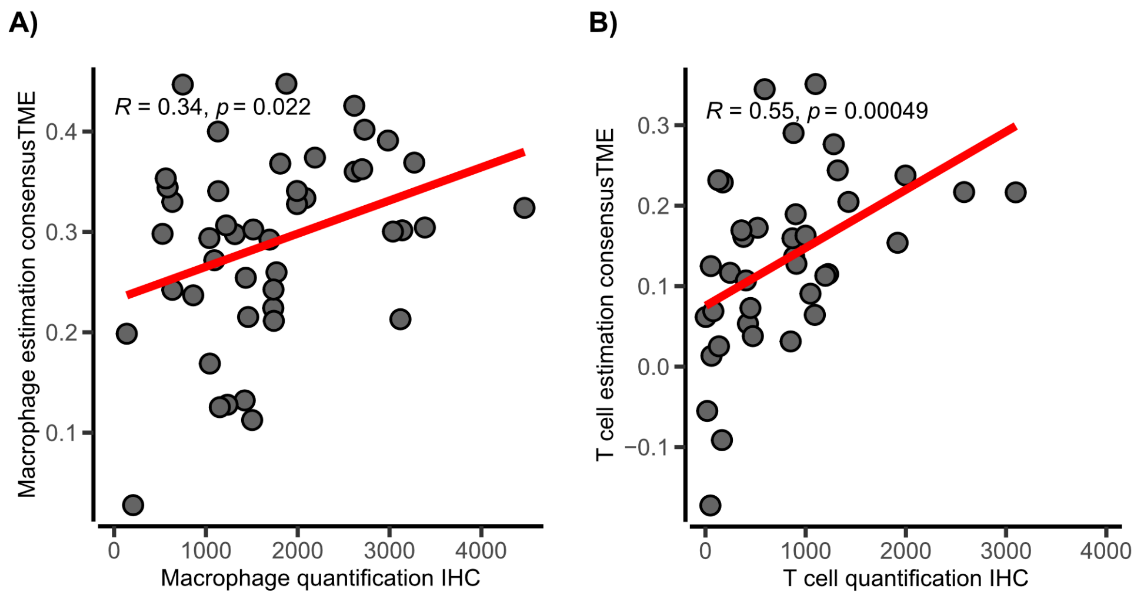

Supplementary Figure 20. Immunohistochemistry (IHC) validation of immune population estimates by consensusTME from TRACERx Renal bulk RNA-Seq data. *Scatter plots show the Pearson's correlation between A) IHC and consensusTME estimates of macrophage abundance (IHC measures CD68 marker) (n = 47), and B) IHC and consensusTME estimates of T cell abundance (IHC measures CD3 marker) (n = 39).*

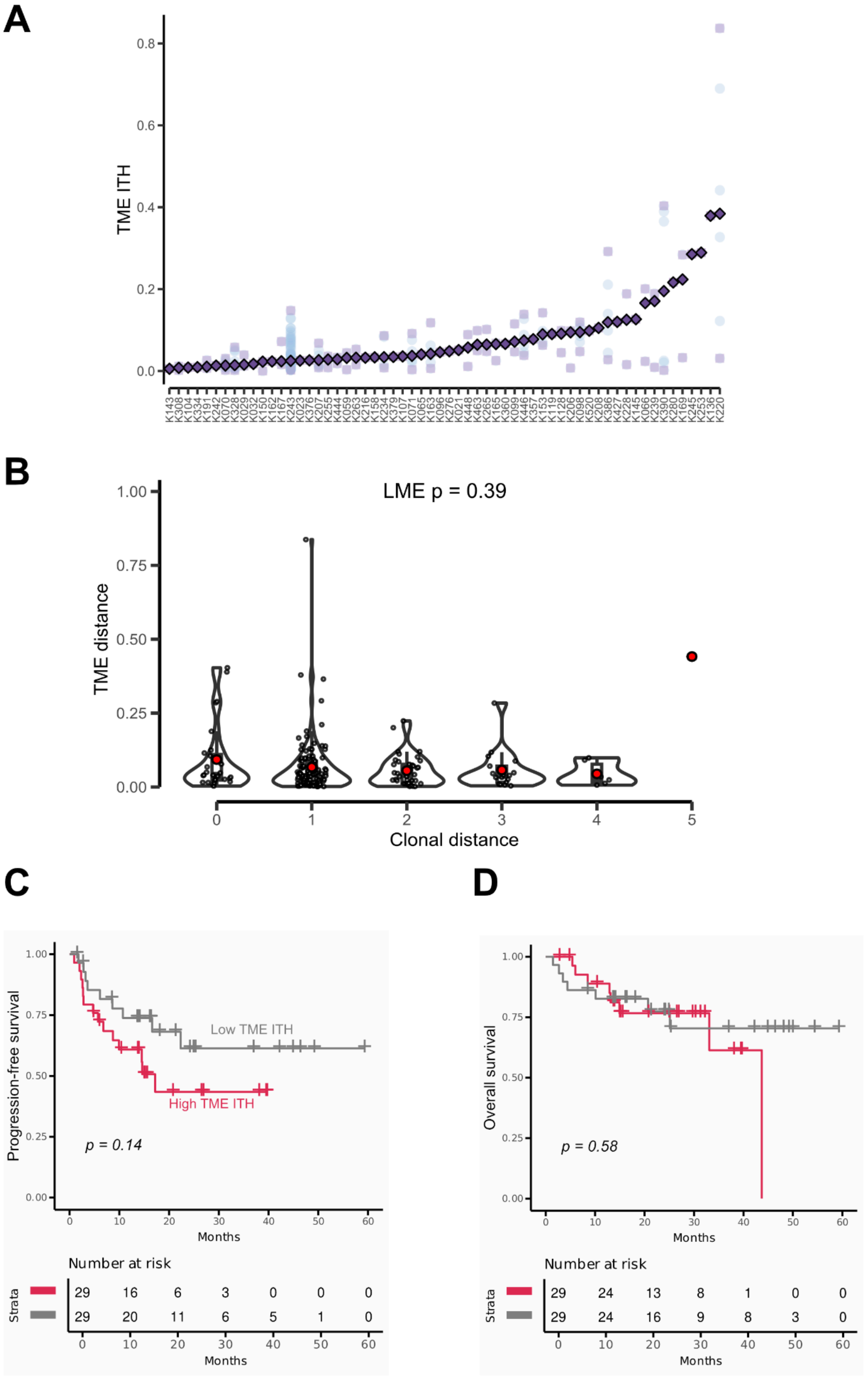

Supplementary Figure 21. TME ITH is pervasive in ccRCC and is not associated with poorer clinical outcomes. *A) Primary tumor TME ITH (I-TED applied to cell abundance counts, see Methods) across 60 TRACERx Renal patients with at least two regions sampled. Pale purple squares represent pairs with minimum and maximum TME distances; blue points represent the TME distance between the rest of sample pairs, if available. B) TME distances between 285 pairs of primary tumor*

samples with increasing clonal distances, defined as the distance between clones located in two different monoclonal regions. Red points indicate the mean TME distance between pairs of samples with the same clonal distance. C,D) C) Progression-free survival and D) overall survival *Kaplan-Meier curves stratified by TME ITH values above (red) or equal and below median (gray). p-value is obtained by a log-rank test.*

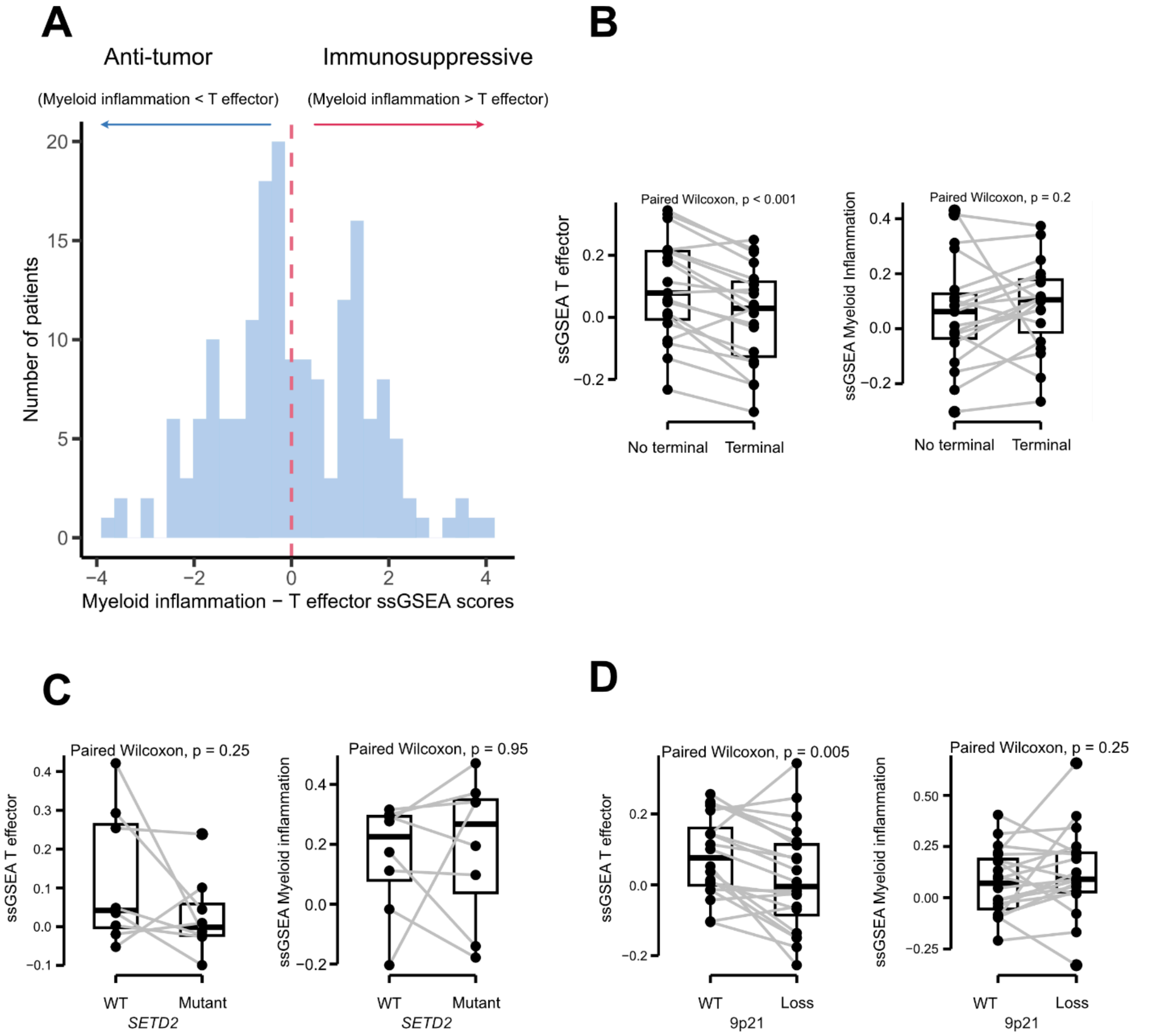

Supplementary Figure 22. Relative depletion of T effector signature underpins TME transitions from antitumor to immunosuppressive microenvironments. *A) Distribution of Z-score of myeloid inflammation signature minus Z-score of T cell effector signature across TRACERx Renal samples. Both signatures were first described by Motzer et al(10). B,C,D) Paired comparison of ssGSEA T effector and myeloid inflammation signatures in B) samples containing terminal and non-terminal clones within the patient phylogenetic tree (19 patients with at least one sample of each type), C) SETD2 mutant and SETD2 wild-type samples (8 patients with at least one sample of each type) and D) 9p loss and 9p wild-type samples (20 patients with at least one sample of each type). Each point represents the mean expression in all samples of each type within a patient. p-value estimated by Wilcoxon paired test.*

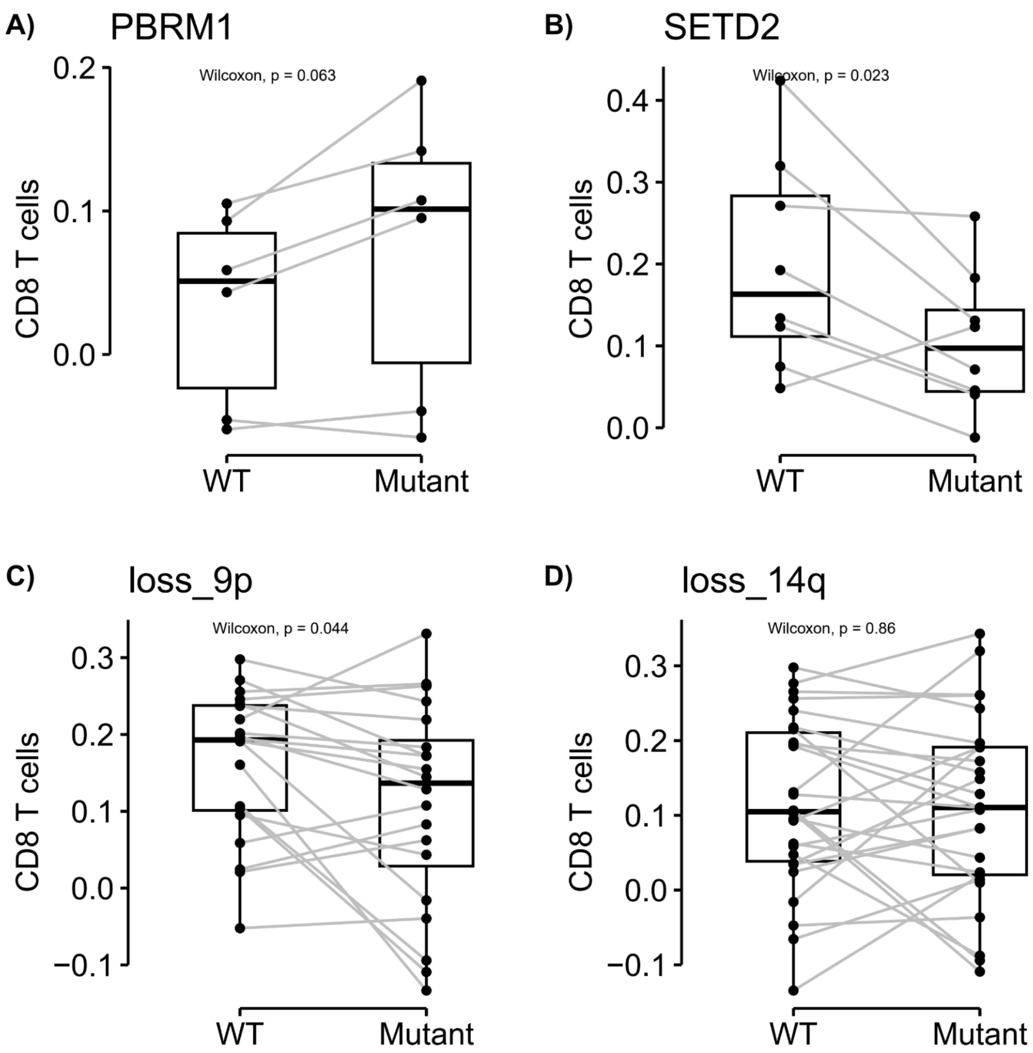

Supplementary Figure 23. Acquisition of subclonal canonical ccRCC drivers correlates with differences in CD8+ T cell abundance in TRACERx Renal. *CD8+ T cell abundance estimated by consensusTME in wild-type (WT) and mutant (Mutant) regions for A) PRBM1 (n = 6 patients), B) SETD2 (n = 8 patients), C) 9p loss (n = 20 patients), and D) 14q loss (n = 26 patients). Only patients with at least one wild-type and mutant primary tumor region are included in each analysis. Each point represents the mean CD8+ T cell abundance across all samples of each type within a patient. p value estimated by Wilcoxon paired test.*

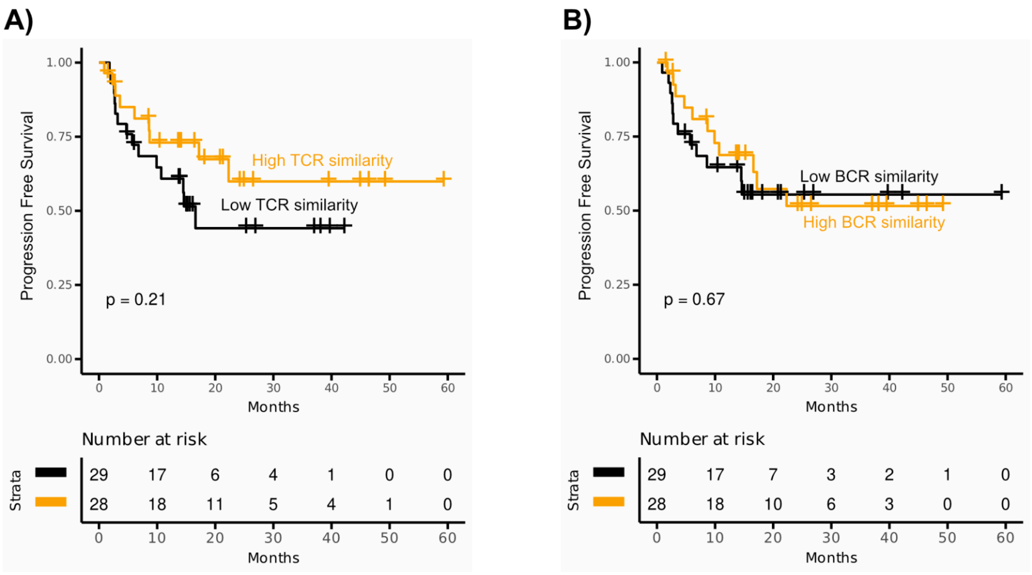

Supplementary Figure 24. Overall survival by per-patient global BCR and TCR similarity in TRACERx Renal. *Kaplan-Meier progression-free survival curves stratified by A) per-patient TCR similarity and B) per-patient BCR similarity. In both cases, “high” is defined as above median and “low” as below or equal to the median. p value obtained by log-rank test.*

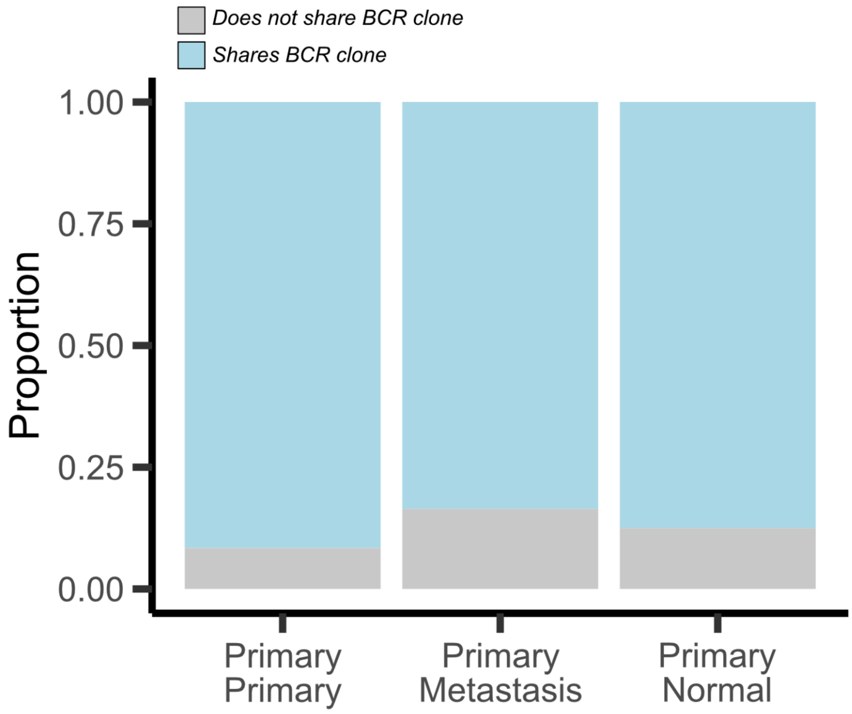

Supplementary Figure 25. BCR clones are shared across distant samples in TRACERx Renal. *Percentage of primary-primary, primary-metastasis, and primary-normal pairs from the same patient that share (blue) and do not share (gray) at least 1 BCR clone.*

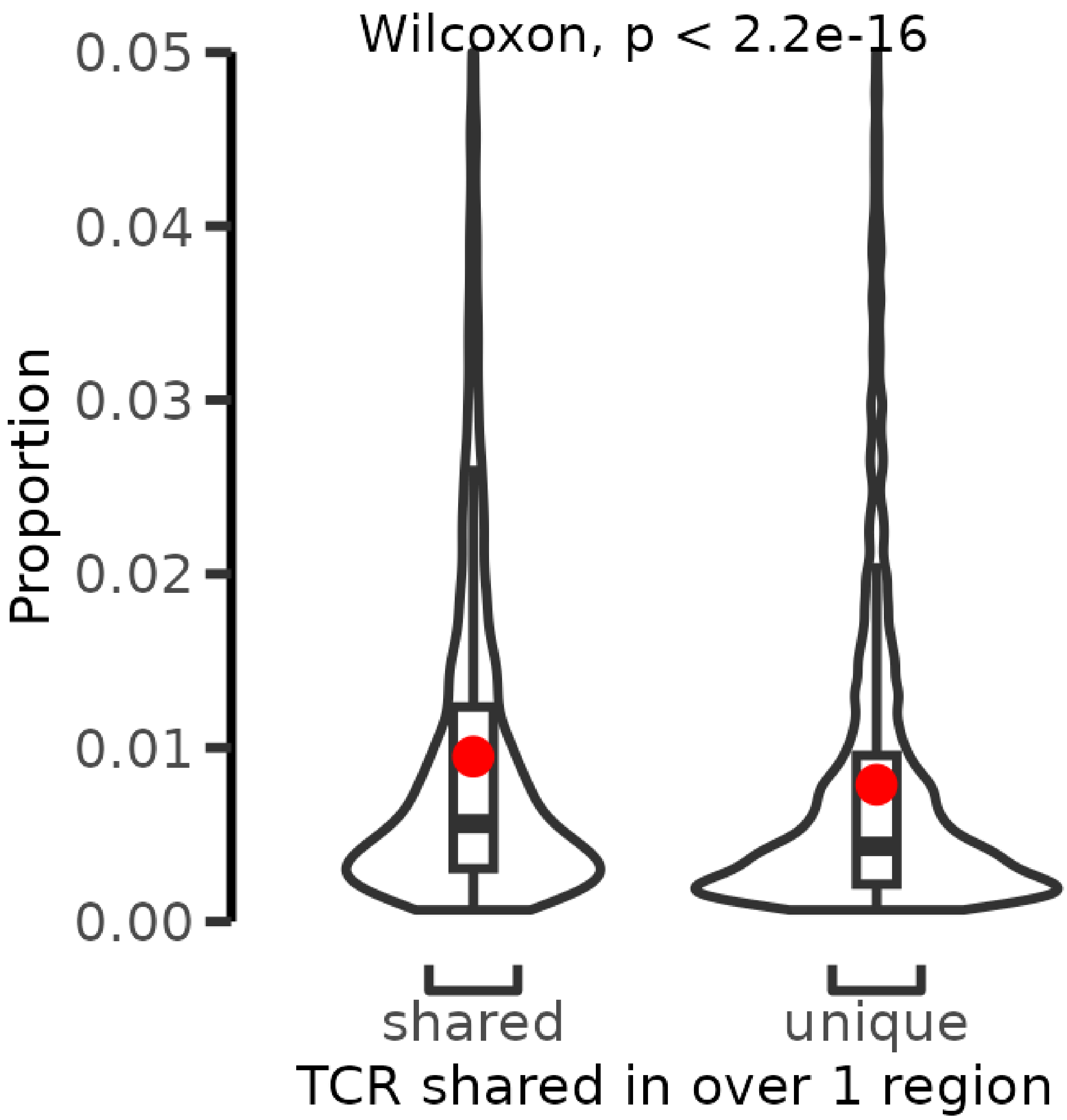

Supplementary Figure 26. Shared TCR clones have higher clonality in TRACERx Renal. *Mean clonality (y-axis) of shared (identified in more than 1 patient tumor region) and unique (private to a single patient tumor region) TCR clones. Red points indicate the mean TCR clonality for each type of TCR clones.*

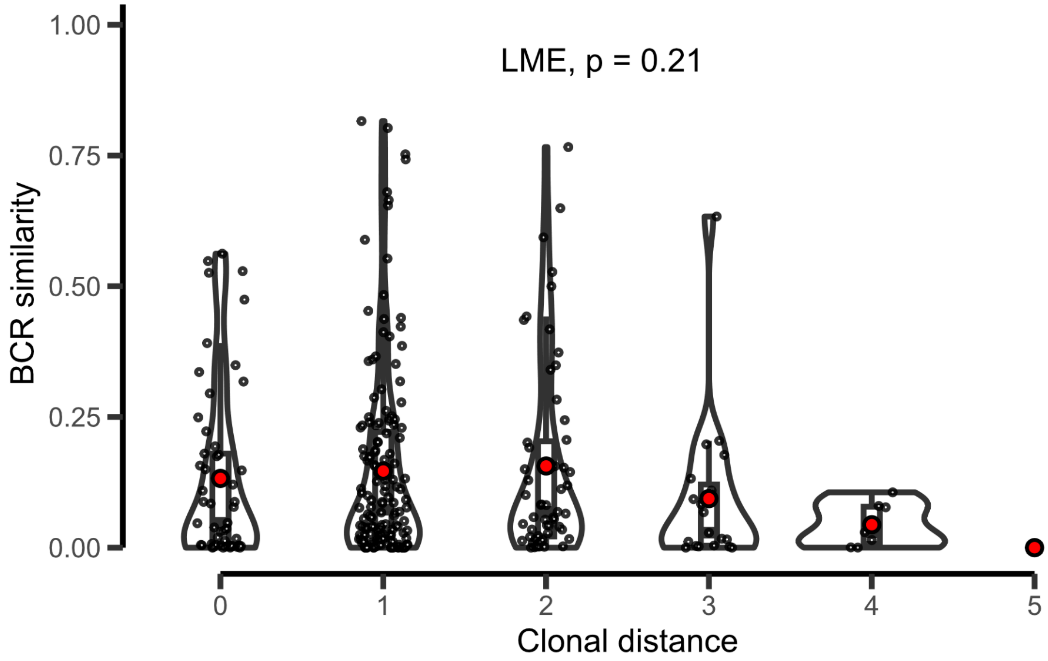

Supplementary Figure 27. Changes in the BCR repertoire do not track with tumor clonal evolution in TRACERx Renal. *BCR similarity in tumor pairs with increasing clonal distances, defined as the distance between clones located in two different monoclonal regions. Red points indicate the mean BCR similarity between pairs of samples with the same clonal distance. p value estimated using linear mixed effects model (LME) to correct for the inclusion of multiple pairs from the same patient.*

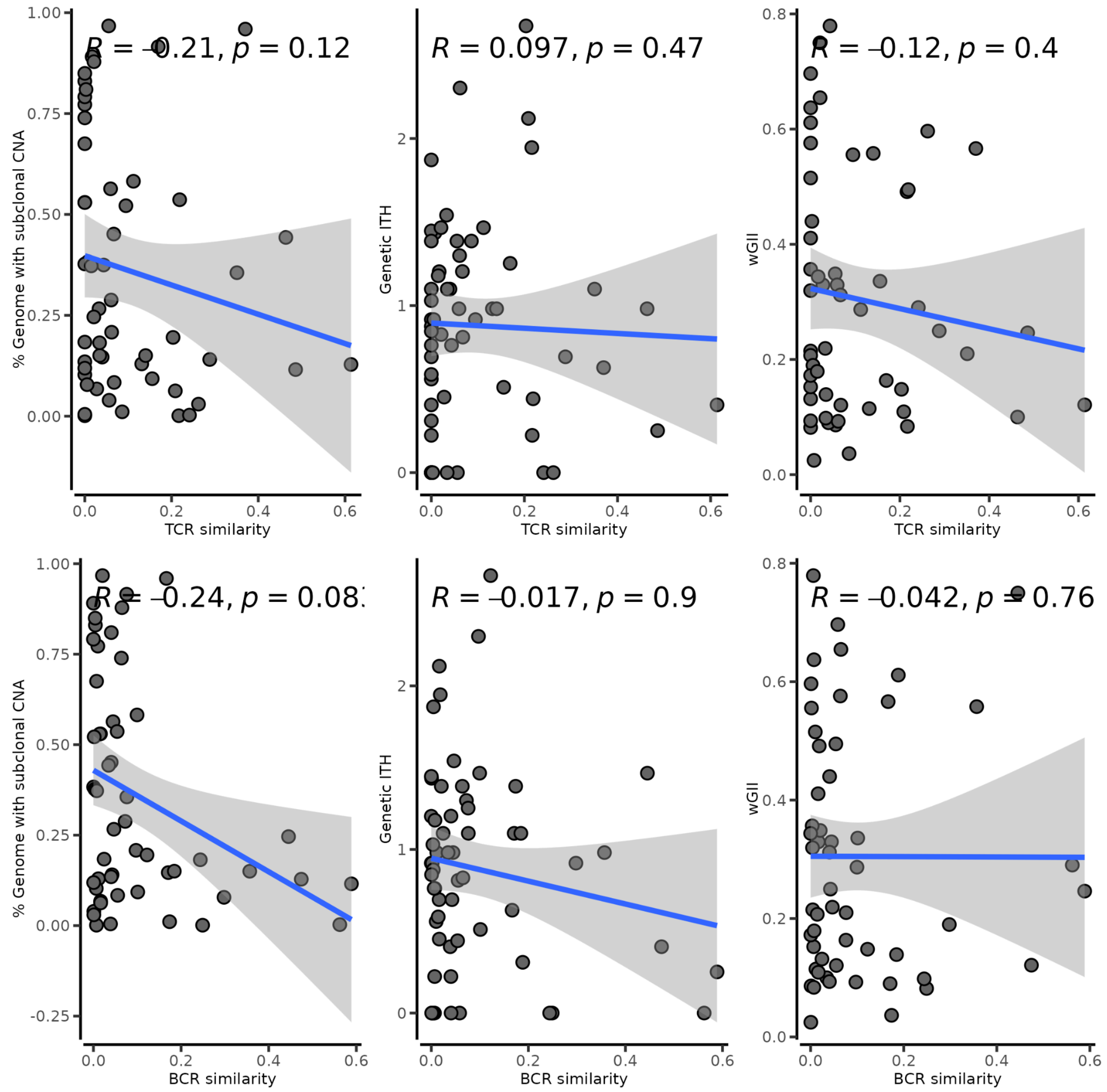

Supplementary Figure 28. Changes in the BCR and TCR repertoire are not exclusively underpinned by intra-tumor genetic heterogeneity. Scatter plots indicate the correlation between per-patient TCR similarity (top row) and BCR similarity (bottom row) and genetic ITH, copy-number heterogeneity (CNH) and aneuploidy burden (weighted genome instability index, wGII). TCR and BCR similarity are calculated in 60 patients with more than one available primary tumor sample, applying the Morisita-Horn index to the TCR or BCR repertoire of the sequenced primary tumor samples for a given patient. Genetic ITH was estimated previously by *Turajlic et al(2)* and is represented in a log-transformed scale. CNH is calculated as the fraction of the genome displaying different copy-number values across tumor samples. wGII is presented as an overall, per-patient estimate, by taking the median wGII observed in sequenced primary tumor samples from a patient.

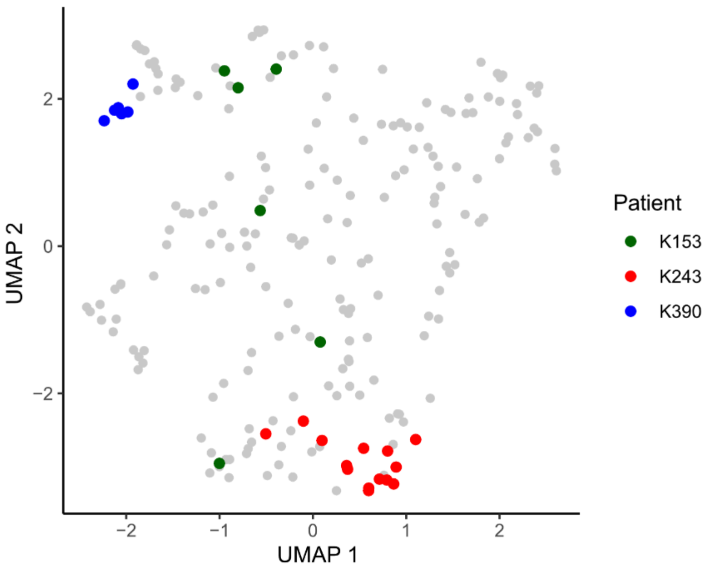

Supplementary Figure 29. Patterns of intra-patient and inter-patient HERV expression heterogeneity in TRACERx Renal. *Uniform manifold approximation and projection (UMAP) visualizing the variation in HERV expression within and across tumor samples. Samples from patients K390, K243, and K153 are colored to highlight varied levels of HERV expression intra-patient variation.*

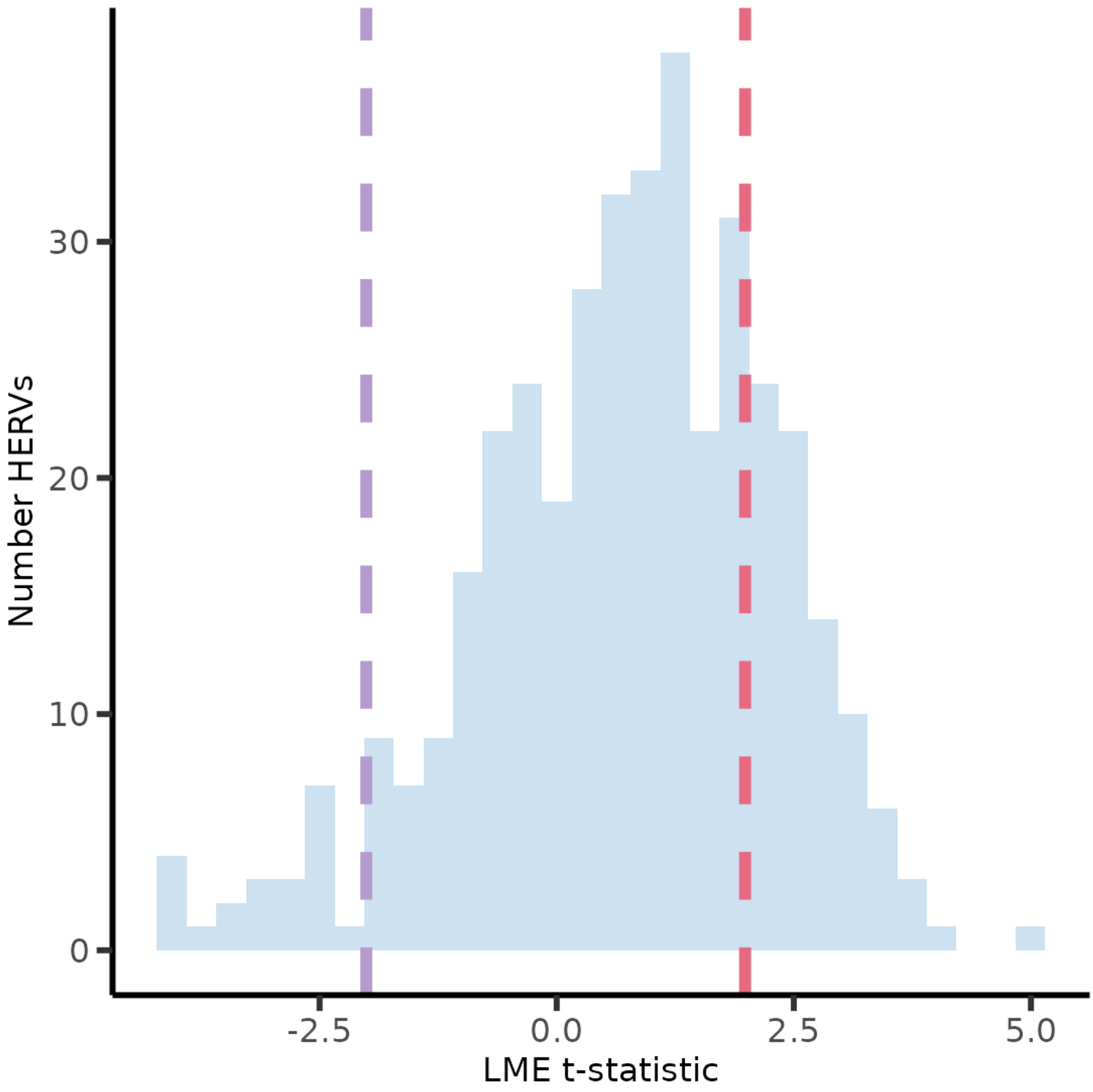

Supplementary Figure 30. Association between HERV/LTR expression and copy-number of its genomic locus. Results of a mixed-effects model linear regression between HERV/LTR expression and matched copy-number status across all TRACERx Renal samples. *VHL* mutational/methylation status and tumor purity were included as fixed covariates in the model, while controlling for multiple inclusion of samples from the same patient (see *Methods*). Strength of the association between copy-number and HERV/LTR expression is represented in the x-axis. Red and purple lines indicate the thresholds upon which we observe significant positive and negative associations, respectively, between HERV/LTR copy-number and HERV/LTR expression.

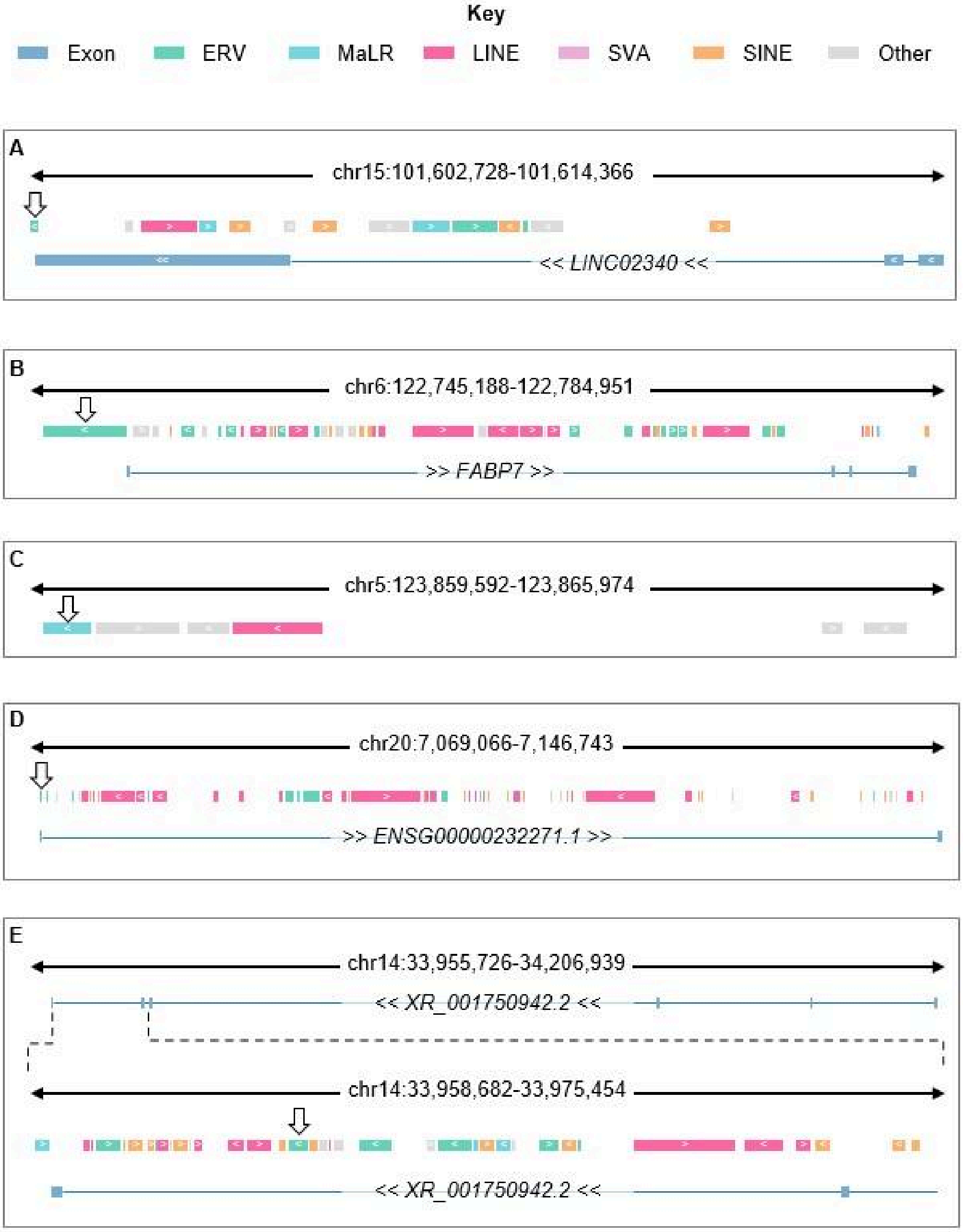

Supplementary Figure 31. **LTR-containing elements significantly differentially expressed in VHL loss of function and VHL wildtype tumor samples are embedded within transcripts.** The genomic context of the LTR-containing elements is shown, with the element of interest indicated by an arrow. A) trans22d47719a5c336d7, B) trans34d544f8cd0f290b, C) trans4bb9fd377e12cfda, D) trans4d254a36cd5f15bd, E) transefa254304db02f03.

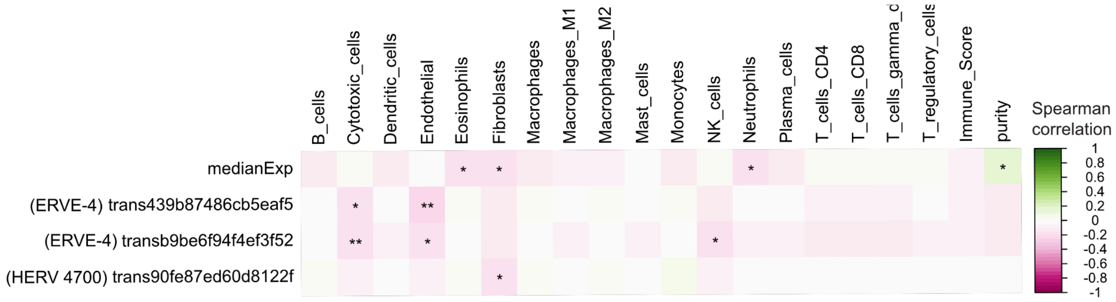

Supplementary Figure 32. Overall and individual HERVs expression correlates weakly with granulocyte abundance, but not with cytotoxic cell content. *medianExp stands for median expression across all identified HERVs. 3 transcripts (associated with HERV 4700, ERVE-4, and ERVE-4) are individually highlighted given previously reported associations with anti-tumor T cell responses in ccRCC (9–12). Asterisks indicate correlations which are significant (FDR < 0.05).*

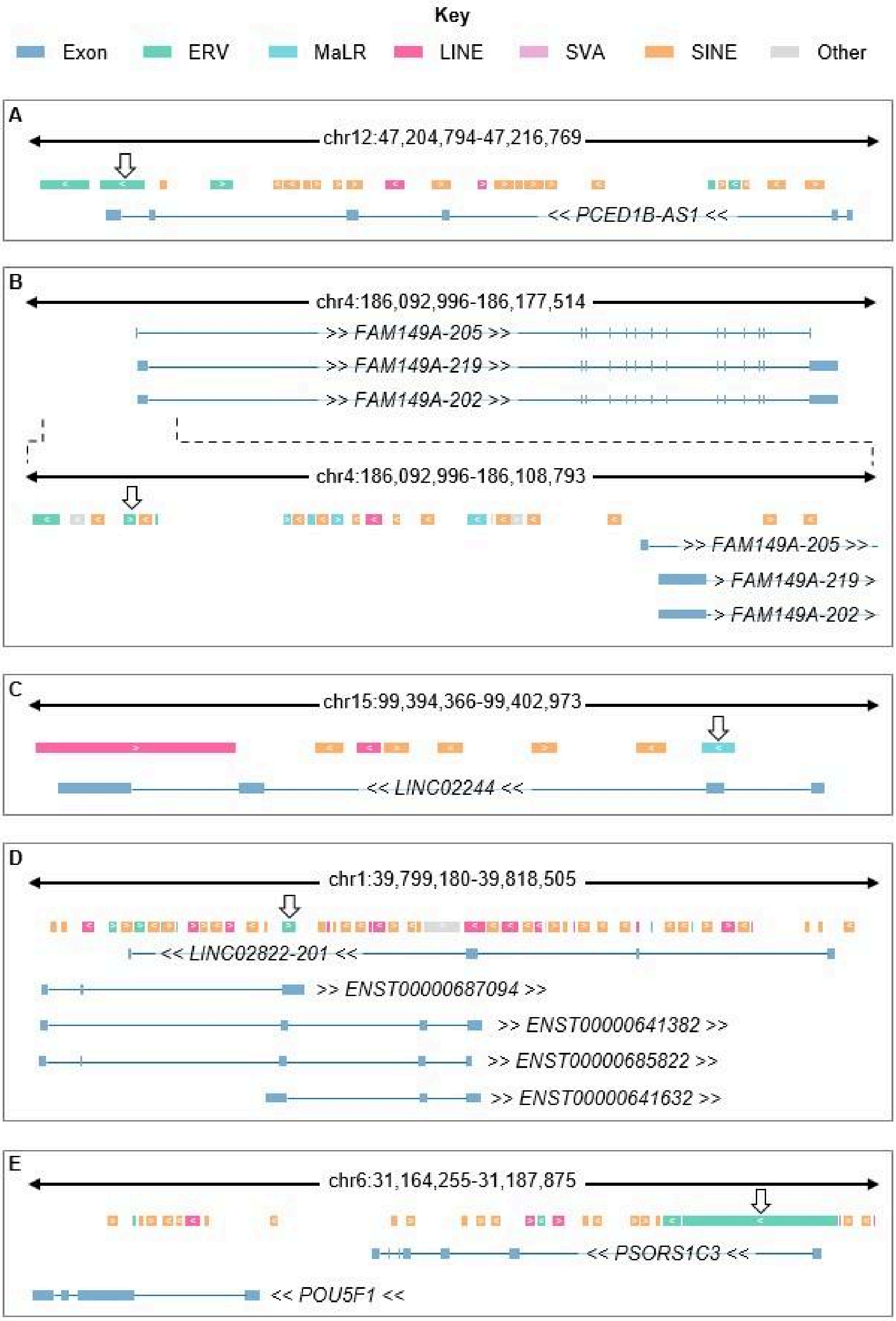

***Supplementary Figure 33.* LTR-containing elements significantly associated with patient survival are embedded within transcripts.** The genomic context of the top 5 LTR-containing elements associated with survival in the TRACERx Renal cohort is shown, with the element of interest indicated by an arrow. A) trans9e3ed2599e6162ea, B) transe3b540fc6c7e9c45, C) trans20145ef1c2c8603b, D) trans82d0b1a4ffe011d1, E) trans8c3c855ce694777a.
